# Combinatorial multiomic analysis from a pedigree of *Sox10^Dom^* Hirschsprung mice identifies multiple high confidence candidate modifiers of Enteric Nervous System development

**DOI:** 10.1371/journal.pcbi.1014424

**Published:** 2026-07-06

**Authors:** Joseph T. Benthal, Justin A. Avila, Jeffrey R. Smith, E. Michelle Southard-Smith

**Affiliations:** 1 Program in Human Genetics, Vanderbilt University, Nashville, Tennessee, United States of America; 2 Vanderbilt Brain Institute, Vanderbilt University, Nashville, Tennessee, United States of America; 3 Stowers Institute for Medical Research, Kansas City, Missouri, United States of America; 4 Genetic Medicine, Vanderbilt University School of Medicine, Nashville, Tennessee, United States of America; Burnet Institute, AUSTRALIA

## Abstract

Hirschsprung disease (HSCR) is characterized by absence of enteric ganglia (aganglionosis) along variable lengths of the distal intestine. This disorder results from deficient colonization of fetal intestine by enteric neural crest-derived cells (ENCDCs). HSCR exhibits complex, multifactorial inheritance with penetrance and severity varying widely even within families. *SOX10* is among causal genes that predispose to aganglionosis. Yet, how gene interactions influence severity of HSCR aganglionosis is not understood. Prior mapping of aganglionosis modifiers was achieved in a standard F_1_-intercross utilizing the *Sox10*^*Dom*^ HSCR mouse model. Here we deploy a novel strategy of genotyping an extended pedigree pedigree of *Sox10*^*Dom*^ mice on a mixed genetic background. GWAS in this pedigree points to novel aganglionosis modifier intervals with replication and refinement of prior modifier regions. Complementary omics analysis of the developing Enteric Nervous System (ENS) enabled identification of multiple high-priority candidate genes within these modifier intervals based on gene expression, chromatin accessibility, and presence of conserved SOX10 binding motifs. We implemented a prioritization pipeline for ranking potential modifiers that generated candidate lists including several well-known for effects on ENS development as well as multiple novel genes. Among the novel genes, *Dach1* ranked as a top priority candidate gene for modifying migration of ENCDCs and thus influencing aganglionosis severity. The results identify genome intervals with intrinsic genes that are logical candidates for modifying *Sox10*^*Dom*^ aganglionosis severity. We also note that several human orthologs to aganglionosis modifier candidate genes are within linkage disequilibrium blocks containing genetic variants associated with human gut motility disorders, which offers opportunity for gaining biological insight into human HSCR severity.

## Author summary

Hirschsprung disease is a complex genetic neurodevelopmental disorder that produces absence of neurons in the distal bowel. The length of gut lacking neurons, called “aganglionosis”, in HSCR patients can vary widely even between affected siblings. Multiple genes are Mendelian causative for HSCR, but little is known about the gene interactions responsible for the notable variation in aganglionosis severity. In this study, we use a mouse model of HSCR to identify genomic regions, “modifiers”, associated with length of aganglionosis. Genes active within these regions are then identified in RNA-sequencing and open chromatin data from progenitor cells, which form the enteric nervous system. These omics approaches identify both known and novel genes that can affect enteric neuron development and may underly HSCR severity in patients. *Dach1,* already known for effects on neuronal progenitor proliferation and migration in other aspects of the nervous system, emerged as a top priority gene. The findings greatly expand the gene network that influences HSCR aganglionosis.

## Introduction

The ENS is essential for normal gastrointestinal (GI) motility and analyses of mouse models have identified genes that are essential for ENS development. ENS neurons and glia that make up the myenteric and submucosal ganglia along the entire length of the intestine are formed by ENCDCs that colonize the fetal gut during development [1]. Gut colonization begins at 9.5 days post coitus (dpc) in mice as neural crest cells invade the foregut and then migrate along the full length of the gut by 14.5dpc [1]. The wavefront of migrating enteric neural crest-derived cells (ENCDCs) leaves behind cells that differentiate into neurons and glia. Disruption of initial ENCDC migration can produce GI motility disorders such as HSCR or Waardenburg-Shah syndrome while disrupted differentiation of ENCDCs is thought to contribute to chronic intestinal pseudo-obstruction [2,3]. The characteristic phenotype for HSCR is aganglionosis—lack of enteric ganglia—of the distal colon at varying lengths, caused by deficient colonization of ENCDCs [1]. Multiple mouse models mimic human HSCR, including *Sox10*^*Dom*^ [4]. The *Sox10*^*Dom*^ HSCR model also recapitulates the variability of aganglionosis length that remains a poorly understood characteristic of human HSCR [5]. Mouse models on controlled genetic backgrounds offer opportunity to identify contributing genes to the variability of HSCR aganglionosis.

Genetic mapping studies in HSCR mouse models to find genes contributing to aganglionosis severity have had limited success due to large genomic intervals from standard crosses and lack of ENCDC expression data for candidate gene prioritization. Initial work to define how genetic background affects aganglionosis was performed by Cantrell et al. who compared lengths of intestinal aganglionosis in congenic C3HeB/FeJ and C57BL/6J.*Sox10*^*Dom*^ strains (2004). C57BL/6J.*Sox10*^*Dom*^ mice exhibit notably more severe aganglionosis than C3HeB/FeJ.*Sox10*^*Dom*^ mice [6]. Owens et al. mapped five broad genomic modifier intervals (ranging from 8 to 30 cM) associated with aganglionosis via genome-wide linkage scan of *Sox10*^*Dom/+*^ F_2_ progeny derived from C3HeB/FeJ and C57BL/6J congenic lines (2005). Further evidence that gene interactions can alter extent of aganglionosis was reported by Maka et al. [7], who found that loss of *Sox8* increases extent of aganglionosis in crosses with *Sox10*^*lacZ*^ knockout mice (2005). Multiple gene defects contributing to the severity of HSCR aganglionosis were also demonstrated for *Ret*^*+/-*^;*Ednrb*^*S/S*^ mutants [8]. Despite the genetic evidence of genome regions that modify aganglionosis phenotype, the ability to identify potential candidate genes within the regions that produce these effects has been limited by lack of gene expression and chromatin accessibility data for the developing fetal gut and specifically the migrating ENCDCs that form mature neurons and glia of the ENS.

There has been recent success in bulk and single cell sequencing of ENS progenitor populations that offers new avenues to prioritize candidate genes within modifier intervals. Stavely and colleagues recently utilized bulk RNA-seq of ENCDCs at the migrating wavefront and the cells behind the wavefront to identify differentially regulated genes at 11.5dpc [9]. In addition, Zhao and colleagues performed scRNA-seq on entire mouse gut over the time course of ENCDC migration (2022). This dataset allows detection of genes that are expressed either within the ENCDCs or in the surrounding gut environment, an important aspect, since interactions between progenitors and the fetal gut mesenchyme are known to influence extent of migration. Such datasets offer unique opportunities to prioritize candidate genes that might be functional among cells that contribute to aganglionosis phenotype.

Prior genetics analyses of human HSCR have focused primarily on Mendelian causes. Relatively limited efforts have pursued the characteristic variability of HSCR disease penetrance and severity. Multiple case-control GWAS have identified variants associated with human HSCR [10–13]. These studies identified common genetic variation at *RET*, *NRG1*, and *SEMA3C/D* loci that are significantly associated with HSCR. A study investigating additional phenotypic anomalies that can accompany HSCR implicated copy number variation at the *SOX2*, *MAPK10*, *ZFHX1B*, *PHOX2B*, and *SEMA3A* loci; severity of aganglionosis in these patients was not considered [14]. To date, seven human studies identified variants or genes that influence the penetrance of HSCR [10,14–19]. To our knowledge, no human study has undertaken quantitative analysis to identify genetic variation modifying the extent of HSCR aganglionosis length, which is responsible for clinical severity. In contrast, mouse models of HSCR enable precise measure of aganglionosis length on controlled genetic backgrounds to dissect genetics underlying severity.

Given the ability to control genetic background in mice and combine transcriptomic resources to prioritize candidate genes, we sought to refine *Sox10*^*Dom/+*^ modifier intervals and utilize omics datasets to identify candidate genes that could influence the length of aganglonosis. We investigated a 10-generation pedigree of 830 heterozygous affected *Sox10*^*Dom*^ mice that yielded improved resolution, capitalizing upon the increased number of meiotic recombination events relative to standard F_2_ intercrosses. The pedigree analysis accomplished this by reintroducing the greater-effect alleles of the B6 genetic background at each generation [6]. The *Sox10*^*Dom*^ line was maintained by crosses of affected heterozygous males with wild type (WT) F_1_ B6C3Fe-*a*/*a* females. Pedigree individuals were genotyped with a linkage mapping SNP set appropriate for its genetic resolution. Genome-wide association in this pedigree improved the resolution of known loci, and identified novel loci that modify aganglionosis length, including some with sex-biased effects. Modifiers intervals were still very large containing hundreds to thousands of genes. Thus, candidate genes from each modifier interval were prioritized based on gene expression among ENCDCs and fetal gut during ENCDC migration, chromatin accessibility in fetal enteric neuronal progenitors, and conserved SOX10 transcription factor binding motifs. From the many genes within modifier intervals, 19 met multiple genetic and omics criteria. Among these we observed both novel and known genes involved in ENS development including *Ednrb*, *Nrg1*, *Col1a1*, and *Phox2b*. In the pedigree, *Phox2b* and *Mcm3* appeared to be sex-biased modifiers. A top candidate gene identified through the omics analyses was *Dach1*, which has not previously been associated with aganglionosis, yet is expressed in migrating ENCDC, is flanked by SOX10 conserved binding motifs, and resides within accessible chromatin in ENS neuronal progenitors. The modifier genes identified from this analysis are likely to influence ENCDC migration or differentiation in the developing gut.

## Results

### Genome-wide SNP analysis identifies *Sox10*^*Dom*^ aganglionosis modifier intervals

**Fig 1:**
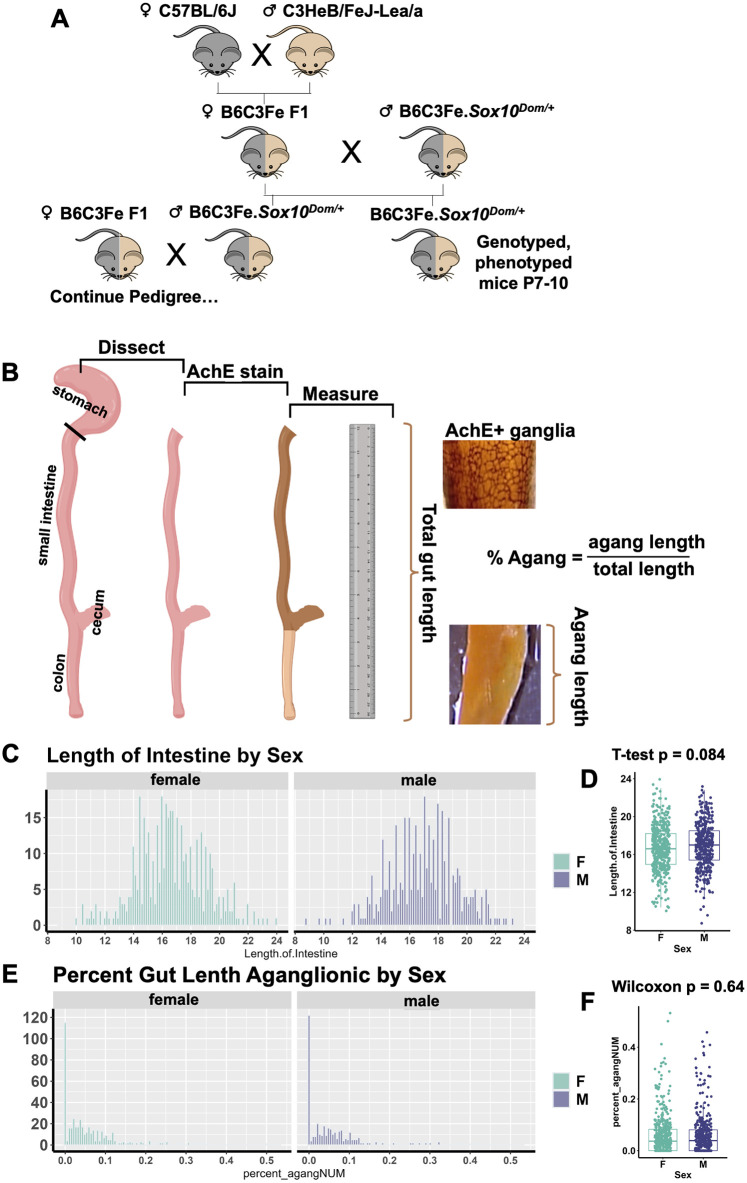
Phenotype distribution of *Sox10*^*Dom*^ pedigree mice exhibits no significant separation of phenotype by sex. **(A)** Schematic of the breeding strategy used for the *Sox10*^*Dom*^ mouse extended pedigree. **(B)** Schematic illustrating quantitation of aganglionosis extent as a proportion of total gut length affected by aganglionosis in *Sox10*^*Dom*^ pups. Collection of gut from the pyloric sphincter to the anus, followed by Acetylcholinesterase wholemount staining, with measurement of total gut length and the region of aganglionosis allowed calculation of the percent gut length affected by aganglionosis. **(C,D)** Length of intestine split by sex displayed on histograms and a box plot shows normal distribution and no significant difference between males and females. **(E,F)** Length of intestinal aganglionosis split by sex on histograms and a box plot shows a non-normal, zero-skewed distribution with no significant difference between males and females. Panel B was partially generated in Biorender (Southard-Smith, M. (2026) https://BioRender.com/7osgcym).

While maintaining the B6C3Fe-a/a.*Sox10*^*Dom/+*^ strain, we observed notable phenotype variability among *Sox10*^*Dom/+*^ pups produced by iterative outcrosses to B6C3Fe-*a*/*a* wildtype females with some pups dying from severe aganglionosis at postnatal day (P)7 while others survived beyond a year of age. To understand genetic variation associated with severity of aganglionosis, we quantitatively assessed aganglionosis for 830 P7-10 B6C3Fe-a/a.*Sox10*^*Dom/+*^ pups. *Sox10*^*Dom/+*^ pups were distinguished from their WT littermates via characteristic white ventral spotting, white feet, and confirmed by direct genotyping of the mutation [6,20]. Extent of aganglionosis was assessed by whole-mount acetylcholinesterase staining collected over multiple pedigree generations (Fig 1A and 1B and S1 Table) using established methods [6,20]. Because *Sox10*^*Dom*^ pedigree maintenance relied on breeding *Sox10*^*Dom*^ males to WT F_1_ B6C3Fe-*a*/*a* female mice, alleles for greater severity/aganglionosis were reintroduced at each generation. Reduced severity/protective alleles were selected for by mating *Sox10*^*Dom*^ males that survive to sexual maturity. A standard mouse linkage mapping panel, including 1449 SNPs of which 876 were informative in the pedigree, was used to genotype each *Sox10*^*Dom/+*^ pup (S2 Table). No significant difference between males and females were detected in length of intestine or length of aganglionosis (length of intestine t-test p = 0.084; length of aganglionosis Wilcoxon p = 0.64; Fig 1B–1E). Rather than being normally distributed, we observed that the aganglionosis phenotype in *Sox10*^*Dom/+*^ pups was zero-inflated due to animals that lacked detectable aganglionosis (Fig 1D), which replicates prior reports of aganglionosis distribution in this Hirschsprung model [6,20].

**Fig 2:**
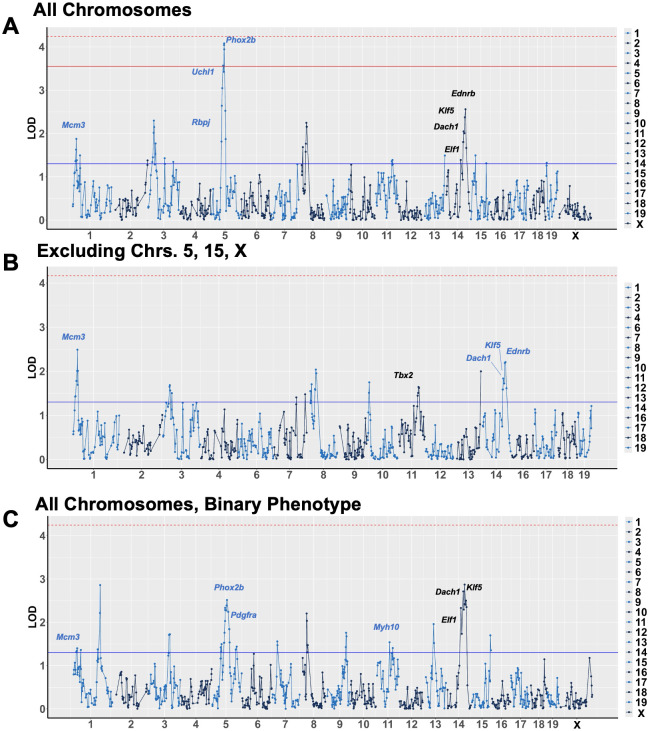
GEMMA genome-wide scans using phenotype variation and a binary phenotype of aganglionosis in the *Sox10*^*Dom*^ pedigree identify genome regions associated with aganglionosis. **(A)** Manhattan plot visualizing association analysis of quantitative aganglionic length with inclusion of all chromosomes. **(B)** Analysis of quantitative aganglionic length as in A, with exclusion of chromosomes 5 (harboring *Phox2B*), 15 (harboring *Sox10*), and X (with potential to accentuate alternative loci). **(C)** Alternative analysis comparing *Sox10*^*Dom*^ mutation carriers that are unaffected to carriers with any affected aganglionic length (binary analysis). Alternating blue and black traces represent the plotted LOD values for distinct chromosomes with corresponding chromosome number indicated below each trace on the x-axis. Blue lines on Manhattan plots indicate marginal significance (unadjusted P-Wald<0.05), dotted red lines indicate Bonferroni-adjusted significance (adjusted P-Wald<0.05), and the solid red line indicates false discovery rate significance (adjusted P-Wald<0.05). Gene positions shown are approximate and are candidate modifiers derived from omics evidence summarized in Table 6 after the prioritization process and are not meant to indicate genome wide significant SNPs at these genes.

To identify genomic intervals associated with aganglionic length in the B6C3Fea.*Sox10*^*Dom*^ pedigree, we tested association between SNPs distinguishing genomic intervals originating from the parental C3HeB/FeJLe-a/a (C3Fea) or C57BL/6J (B6) strains with the variable aganglionosis phenotype. Our approach employed Genome-wide Efficient Mixed Model Analysis (GEMMA), which mitigates false positives that could otherwise arise due to relatedness of inbred mouse strains [21]. Measured agangionic length was evaluated as a proportion of total intestinal length to avoid potential artifacts due to pup size. Sex was included as a covariate (443 females, 387 males). We prioritized consideration of associated loci that A) were significant after multiple testing correction, B) had a logarithm of the odds (LOD) score of ≥3, or C) replicated observations of an independent F_1_-intercross study [20]. Even so, we also discuss additional nominally significant loci due the pleiotropic nature of the *Sox10*^*Dom*^ mutation as shown by Owens et al. [20]. In the main GEMMA analysis, an interval on chromosome 5 (LOD score = 3.9) was significantly associated with aganglionic length after false discovery (FDR) rate correction (Figs 2A and S1A and S3 Table). The greatest significance was observed at rs13478309 (chr5:6714120 of mm10, p-Wald = 8.310292e-05); the B6 allele G was associated with increased aganglionosis. This SNP is ~ 48 kb from the transcription start site of *Phox2b* (mm10; [22]). This finding is consistent with the well-known role of *Phox2b* in ENS development [23]. While detection of significant association near *Phox2b* serves as an internal control, we sought to identify other novel candidate modifier genes with the potential to influence aganglionosis extent. Other nominally significant loci were present on multiple chromosomes illustrated in Fig 2A. Four of these are novel by comparison to the prior F_1_-intercross analysis [20]. One of these nominally significant loci was detected on chromosome 15 and does not overlap with *Sox10* (Fig 2A and S3 Table).

To uncover additive effects of variants on other chromosomes and to account for the zero-inflated aganglionosis phenotype distribution, we evaluated two alternative models. First, given *Phox2b*’s known role in ENS development, the known issues that can arise when performing analyses on the X chromosome, and the previously established “leave one chromosome out” method, we opted to perform our analysis excluding the SNPs on chromosomes 5, X, and 15, where *Phox2B* and *Sox10* reside, respectively [23,25–27]. This approach can augment the statistical signal of loci on alternative chromosomes. Using this approach, we observed additional nominally significant modifier loci on chromosomes 7 and 10 (Figs 2B and S1B and S4 Table). Second, many *Sox10*^*Dom*^ mice in our pedigree do not have detectable aganglionosis, which could obscure SNPs associated with length versus the presence of aganglionosis (Fig 1C). To account for this, we converted aganglionosis measurements to a pseudo case-control binary phenotype. We then compared *Sox10*^*Dom*^ mice who exhibited any aganglionosis to mutation carriers without any detectable aganglionosis. Nominally significant genetic association with absence or presence of aganglionosis was observed at the previously detected loci as well as additional regions (chromosomes 1, 3, 5, 7, 9, 13, and 15) (Figs 2C and S1C and S5 Table). Third, to assess SNP effects on only the variance in aganglionosis without effects of those with no detectable deficits, we performed GEMMA analysis using only mice with measurable aganglionosis (S2 Fig and S6–S8 Tables). This approach produced an approximately 30 percent decrease of animal numbers available for the analysis, and therefore we did not pursue this aspect of the analysis further.

These pedigree-based association scan results expanded upon the previously published F_1_-intercross loci modifying *Sox10*^*Dom*^ aganglionosis [20]. The previously observed modifiers on chromosomes 5, 8, 11, and 14 were again detected (Table 1; [20]). Additionally, the pedigree analysis detected multiple novel modifiers on chromosomes 1, 2, 3, 13, and 19 that were nominally significant (Table 1). The chromosome 3 loci observed in this pedigree-based analysis were distinct from that of the intercross (Table 1; [20]). Directions of effect (DOE) for loci replicating in the prior F_2_-intercross study were concordant (S3A, S3B and S3F–S3H Fig, S1 and S2 Tables, see [20] Fig 4). However, several of the loci appeared to have sex-biased effects on aganglionosis in the pedigree, which had not previously been observed in the F_1_-intercross (S3A–S3E and S3I–S3N Fig, S1 and S2 Tables). Given that Hirschsprung disease exhibits a sex bias with males more frequently affected than females [28,29], the potential sex-bias effect on aganglionosis was investigated further in sex-stratified analysis.

**Table 1:** *Sox10*^*Dom*^ aganglionosis modifiers compared to Owens et al.

Chromosome	Owens et al. [20] Modifier Intervals	All Chrs.	Excluding Chrs. 5, 15, X	All Chrs., Binary Phenotype
1	N/A	16278641-20784226; 22740997-22740997; 38996207	11269352-22740997	16278641-20784226; 38996207; 123000666; 134210713-135155923
2	N/A	180126255	N/A	N/A
3	128812206-159812206	23824919-33313826; 38889885-40032479; 85418436; 128292534	30328765-33313826; 40032479	102689164-107129193
5	54586806-70742211	53657080-74395262*	Excluded	45356296-49346495; 61506436-89687694; 119888129-124809848
7	N/A	N/A	96102709; 132998156	28155257-28832210
8	14759018-34759018	20923450; 34853346-40656172	15279371; 17579118-20923450; 34853346-40656172	34853346-40656172
9	N/A	N/A	N/A	101984792-105491662
10	N/A	N/A	9609273-10825090	N/A
11	56299961-89903556	84732367; 88435691	84732367-90536534	68770516; 84732367
13	N/A	116250111	116250111	54952779-57253189
14	86322314-105322314	79020581; 91070578-106773805	91070578-106773805	74709292-102615050
15	N/A	28321743; 82219300	Excluded	97111470-99794398
19	N/A	6211818	N/A	N/A

Comparisons are made between the *Sox10*^*Dom*^ aganglionosis modifier intervals detected in three GEMMA association runs depicted in Fig 2 and the modifier interval from the prior F_1_-intercross study [20]. The intervals from [20] were derived from aligning their supplemental table SNP genotyping sequences and aligning them to the mm10 genome, then taking the starting and ending points from that [20]. The modifiers from this study include all continuous SNPs that reach marginal significance (p-Wald < 0.05). These include isolated SNPs that reach marginal significance. Modifiers on the same chromosome are separated by a semicolon. Asterisk (*) indicates modifier interval whose peak SNP’s adjusted p-value passes the multiple testing correction threshold.

**Fig 3:**
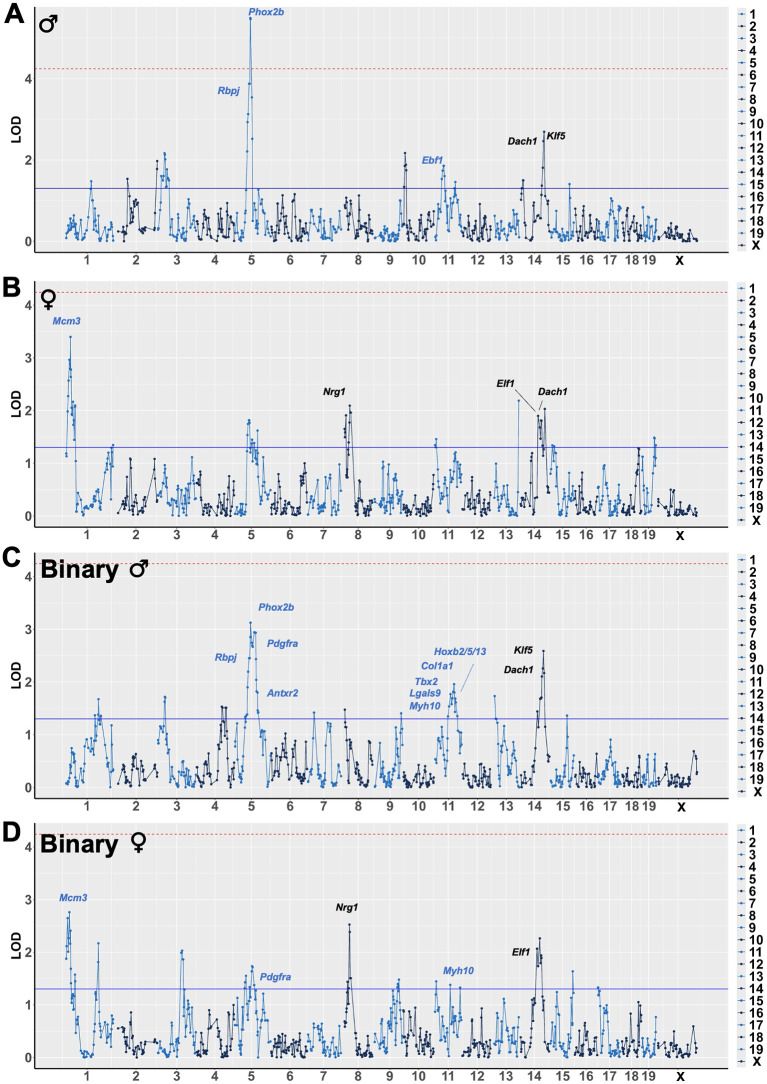
Sex-specific GEMMA genome-wide association for aganglionosis modifiers in the *Sox10*^*Dom*^ pedigree. Manhattan plots visualizing GEMMA genome-wide scan results of male- (**A**) and female-specific (**B**) association analysis of quantitative aganglionosis length. Manhattan plots visualizing GEMMA genome-wide scan results of male- (**C**) and female-specific (**D**) analysis comparing *Sox10*^*Dom*^ mice that are affected by any length of aganglionosis to those mice that are unaffected (binary analysis). Blue lines on Manhattan plots indicate marginal significance (unadjusted P-Wald<0.05) and dotted red lines indicate Bonferroni-adjusted significance (adjusted P-Wald<0.05). Gene positions shown are approximate and are candidate modifiers derived from omics evidence summarized in Table 6 after the prioritization process and are not meant to indicate genome wide significant SNPs at these genes.

**Table 6:** Cumulative omics evidence supporting priority candidate genes within *Sox10*^*Dom*^ aganglionosis modifier intervals.

Candi-date Gene	Modifier Prioritization Score	Gene Differentially Expressed in ENCDCs | Gut Environment	Fetal Gut Environment to ENCDC Estimated Signaling (CellChat)	Number of Conserved SOX10 TFBM	Wavefront Differentially Expressed	Differentially Accessible Chromatin Regions (Prog+NB)	TFBM Enriched	Total Omics Evidence
***Dach1***	1.5	No | Yes		2	Wavefront	38	Yes	5
***Col1a1***	1.5	No | Yes	Gastric Myofibroblasts		Wavefront	4		4
***Pdgfra***	2.5	No | Yes		1	Wavefront	5		4
***Tbx2***	1.5	Yes | Yes		2		4	Yes	4
***Antxr2***	2.5	No | Yes		1		13		3
***Ebf1***	1.5	No | Yes				7	Yes	3
***Ednrb***	1.5	Yes | Yes	Several			16		3
***Elf1***	1.5	No | Yes				1	Yes	3
***Hoxb13***	1.5	No | Yes				2	Yes	3
***Hoxb2***	1.5	No | Yes				1	Yes	3
***Hoxb5***	1.5	Yes | No			Lagging		Yes	3
***Klf5***	1.5	No | Yes				28	Yes	3
***Lgals9***	1.5	No | Yes	Macrophages			7		3
***Mcm3***	1	No | Yes		1		5		3
***Myh10***	1.5	No | Yes			Wavefront	8		3
***Nrg1***	1.5	No | Yes	Telocytes			38		3
***Phox2b***	3.5	Yes | No				17	Yes	3
***Rbpj***	3.5	No | Yes				15	Yes	3
***Uchl1***	3.5	Yes | Yes			Lagging	18		3

Columns “Differential expression based on cell type and developmental stage in either ENCDCs or Gut environment” and “Fetal Gut Cell Type to Fetal ENCDC Signaling” utilized scRNA-seq data (Figs 5, S5 and S6). Column “Number of Conserved SOX10 TFBM” is how many conserved SOX10 TFBMs are intronic to or are near a given gene (Table 5). The fourth column “Wavefront Differentially Expressed” refers to whether a gene was differentially expressed at the migrating wavefront of ENCDCs (Fig 6). “Differentially Accessible Chromatin Regions (Prog+NB)” indicates how many differentially accessible chromatin peaks in ENCDC progenitors or neuroblasts are near a given candidate gene (Fig 7). “TFBM Enriched” indicates whether a candidate gene’s protein’s DNA binding motif was enriched in accessible chromatin (Fig 7). The column “Total Evidence” is a count of how many of the prior columns in which a candidate gene appears. The last column “Modifier Prioritization Score” refers to a candidate gene within a prioritized modifier interval that meets the following criteria: 1) passes multiple testing correction, 2) LOD score of ≥3, and 3) is replicated from the prior F_1_-intercross study (assigned an extra 0.5 point; S25 Table). Those genes that are in modifier intervals that meet all of those criteria are given a score of 3.5. Note: *Plagl1* has 3 modalities supporting it as candidate modifier gene. However, *Plagl1* is an imprinted gene in mice, so it was removed.

### Assessing potential sex-bias among modifiers of *Sox10*^*Dom*^ aganglionosis

Because some of the SNP variants in our pedigree associations exhibited marginally significant sex-biased DOE (S3A–S3E and S3I–S3N Fig, see methods for shorthand terms used for each genome-wide analysis, S1 and S2 Tables), we conducted sex-stratified genome-wide analyses for both quantitative and binary aganglionosis phenotypes using GEMMA [21]. The male-specific genome-wide scan replicated the *Phox2b* on chromosome 5, which was again genome-wide significant (Figs 3A, S1D and S9 Table). This locus was notably attenuated in the female-specific scan. Several other loci were nominally significant in the male-specific scan, including on chromosome 14 near *Ednrb*, a critical gene for ENS development (Fig 3A and S9 Table). The female-specific quantitative scan only yielded nominally significant loci that did not remain significant upon multiple testing correction (Figs 3B, S1E and S10 Table).

The sex-specific binary association analysis only identified nominally significant loci. Male-specific binary phenotype analysis loci mirrored those of the quantitative analysis, but were less significant (Figs 3C, S1G and S1F and S11 Table). Female-specific binary phenotype analysis also yielded only nominally significant variants (Fig 3D and S12 Table). Altogether, these findings suggest that some *Sox10*^*Dom*^ modifiers of aganglionosis severity may exert sex-biased effects (Tables 2 and 3).

**Table 2:** Sex-biased *Sox10*^*Dom*^ aganglionosis modifier locations.

Chromosome	Female	Binary Female	Male	Binary Male
1	9023832-39973796; 195308492	3197400-22740997; 38996207-39973796; 134210713-135155923	105501703	120607495; 134210713-135155923; 144112659-145181359
2	N/A	N/A	58189569; 179522759-180126255	N/A
3	N/A	99031461-107129193	16450289-48327701	30328765-33313826
4	N/A	N/A	N/A	105978113-108710855; 121058843-124195952
5	55224059-64189655; 72797039-74395262; 82505407-83042043; 96638827	44303618-49346495; 64189655; 72797039-83042043	53657080-74395262 *	45356296-46079471; 49346495-99300806
7	N/A	N/A	N/A	28155257-28832210
8	15279371-20923450; 31865138-40656172	27175290; 31865138-40656172	N/A	15279371
9	N/A	103625468-106284925; 109876984	N/A	117918763
10	N/A	N/A	7003953-15800614	N/A
11	6412206-11171143	11171143; 68770516; 109394797	33051317-44208120; 84732367; 88435691	58567550-96604864
13	116250111	N/A	N/A	16340817-20906467
14	79020581-97201128; 106773805	74709292-93124495	10794256-17362990; 93124495-102615050	74709292; 86216087-102615050
15	13219494; 20942406	97111470	82219300	72211956
17	N/A	3388912-4044735	N/A	N/A
19	53438118-55161641; 60081646	N/A	N/A	N/A

This table compares *Sox10*^*Dom*^ aganglionosis modifier genomic intervals from male and female specific GEMMA association runs with those sex-specific GEMMA runs using a pseudo case-control binary aganglionosis phenotype. All modifier locations are position in genome build mm10. The modifiers from this study include all continuous SNPs that reach marginal significance (p-Wald < 0.05). These include isolated SNPs that reach marginal significance. Modifiers on the same chromosome are separated by a semicolon. Asterisk (*) indicates modifier interval whose peak SNP’s adjusted p-value passes the multiple testing correction threshold.

**Table 3:** Top 2 GEMMA peak SNPs per *Sox10*^*Dom*^ aganglionosis modifier interval definition.

GWScan	SNP	Position	Risk Allele	Nearest gene (distance in bp)	Candidate Gene (distance in bp)
AllChrom, Male	rs4225248*	chr5:66662116	A (B6)	*Phox2b* (432283)	*Phox2b* (432283)
NoChrs5,15,X, AllChrom, Male	CEL-14_94845626	chr14:102615050	T (B6)	*Kctd12* (361531)	*Ednrb* (1199575)
NoChrs5,15,X, Female	rs6404446	chr1:20784226	A (C3Fe)	*Il17f* (0)	*Mcm3* (18742)
BinAllChrom	rs13482311	chr14:93124495	T (B6)	*Pcdh9* (0)	*Pcdh9* (0)
BinAllChrom	rs8250053	chr1:135155923	T (C3Fe)	*Arl8a* (0)	*Arl8a* (0)
Female	rs13482028	chr13:116250111	G (C3Fe)	*Isl1* (48170)	*Isl1* (48170)
BinMale	rs3692362	chr14:99776069	C (B6)	*Klf12* (94563)	*Klf12* (94563)
BinMale	rs13478309 & gnf05.061.650	chr5:67147119 & chr5:67348023	G (B6) & C (B6)	*Phox2b* (47818 & 248722)	*Phox2b* (47818 & 248722)
BinFemale	CEL-8_33812776	chr8:34853346	A (B6)	*Tnks* (0)	*Dusp4* (33452)
BinFemale	rs3671256	chr1:16278641	T (C3Fe)	*Stau2* (0)	*Ube2w* (262149)

This table lists the top two peak SNPs per GEMMA association run, of which some are shared between runs. If two SNPs are listed as a peak, these have equal p-values. All modifier SNP positions are in genome build mm10. Nearest genes and potential candidate genes for each peak SNP are listed in the last two columns. Asterisk indicates peak SNP’s adjusted p-value passes the multiple testing correction threshold. Other SNPs are marginally significant and do not pass multiple testing correction threshold.

**Fig 4:**
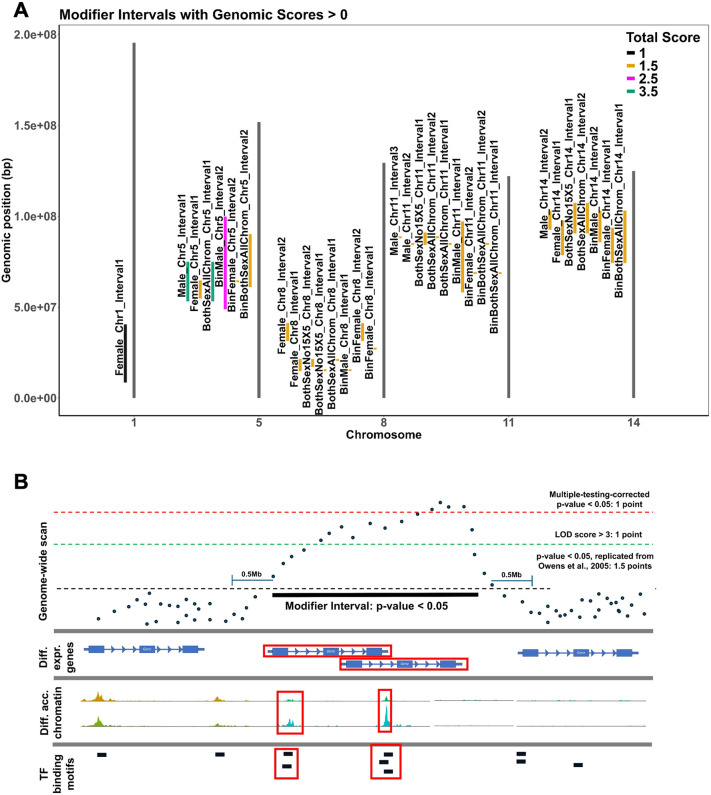
Modifier interval positions and gene level prioritization strategy. **(A)** Schematic displaying overlap and relative position of modifier intervals from each of the GEMMA association studies for aganglionosis severity. Thick gray lines represent the length of each chromosome in basepairs. Relative position and size of modifier intervals derived from each GEMMA analyses that have greater than 0 genomic scores are displayed as colored vertical lines to the left of each chromosome bar. Colors represent assigned modifier interval scores based on the following criteria to minimize false positives: 1 point assigned to peak SNPs of modifier intervals significant after multiple testing correction; 1 point assigned to peak SNPs of modifier intervals with LOD score of ≥3; and 1.5 points assigned to intervals replicated from the prior F_1_-intercross study. **(B)** Schematic illustrating the process of filtering for high priority candidate genes within modifier intervals. Top section depicts significance of SNPs in a GEMMA genome scan with detection of modifier intervals based on genetic scoring criteria. Second level depicts evaluation of genes for expression in relevant tissue (enteric nervous system in this study). Third level depicts overlay of differential chromatin from snATAC-seq. Fourth level depicts relative position for presence or enrichment of TF binding motifs. This strategy was applied to filter for those genes within the modifier intervals that are logical high priority candidates for further study.

In aggregate, the genomic intervals identified from the separate GEMMA association analyses in the *Sox10*^*Dom*^ pedigree (all chromosomes, leave one out, and sex-specific) shared overlap in genome location and localized to chromosomes 1, 5, 8, 11, and 14 (Fig 4A and S13 Table). The span of these intervals in the genome is quite large with some genome intervals exceeding 10Mb. To identify relevant candidate genes within modifier intervals, we implemented a multipronged omics approach to aid in filtering for genes most likely to be affecting ENS development (Fig 4B).

### Prioritization of candidate genes based on expression in the fetal mouse intestine

**Fig 5:**
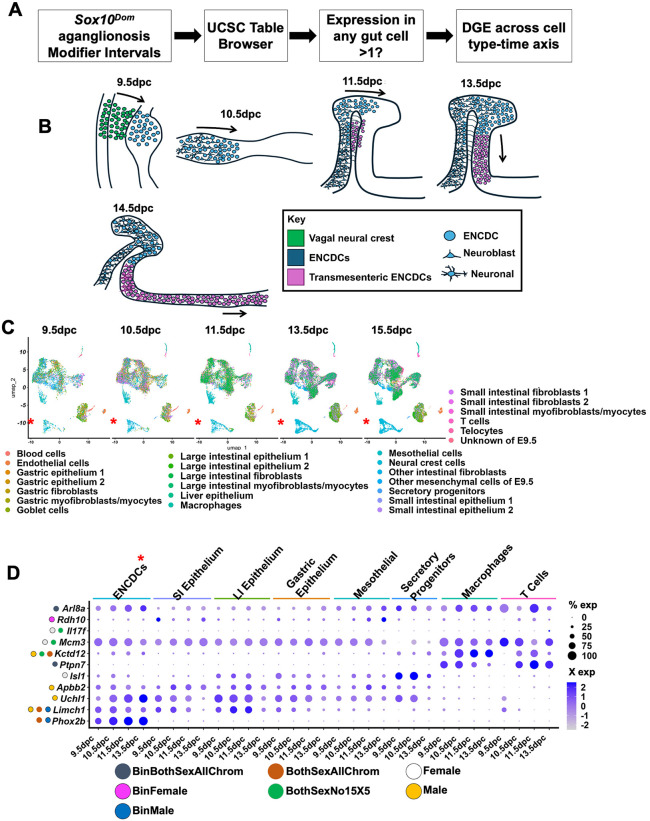
Differential gene expression in the developing gut within aganglionosis modifier intervals filters a first subset of candidate genes. **(A)** Pipeline for prioritizing *Sox10*^*Dom*^ aganglionosis modifier interval genes by differential gene expression. **(B)** Diagram depicting migration of enteric neural crest-derived cells down the length of the fetal mouse gut. **(C)** UMAP of reprocessed scRNA-seq data from [24] split by developmental timepoint and colored by their cell type definitions. The red asterisk indicates the location of the enteric neural crest-derived cells (ENCDCs). **(D)** Dot plot showing expression of differentially expressed genes near the top two LOD peaks per GEMMA GWAS (out of the nearest 10 genes per top 2 LOD peaks, those that are differentially expressed). Cell types have been consolidated to those that are known to be relevant for migrating enteric neural crest-derived cells and those with shared names (numbered) are combined to one identifier. Dots to the left of each gene correspond to which GEMMA genome-wide scan each gene originates.

To identify relevant candidate genes potentially underlying the variability of *Sox10*^*Dom*^ aganglionosis, we first assessed which genes within modifier intervals are expressed in ENS precursor cells and the fetal gut environment during ENCDC migratory stages. Genes within modifier intervals (listed in S13 Table) were identified from the UCSC Table Browser by inputting positions of modifier intervals as elaborated further in Methods. This extraction identified a total of 6216 unique genes (Table 4) after GEMMA runs were consolidated. We first leveraged scRNA-seq data from total mouse fetal gut collected over development from 9.5 to 15.5dpc that profiled ENCDCs and surrounding gut mesenchyme (Fig 5A–5C; [24]). After reprocessing this scRNA-seq data, we filtered for genes within modifier intervals that have log-normalized pseudobulk expression greater than 1 (Tables 4 and S14). Differential gene expression analysis for the expressed genes by age and cell type determined which genes are expressed in specific cell types and timepoints in the developing mouse gut (Tables 4 and S15). We detected differential expression of *Phox2b* and *Ednrb* (Figs 5C and S4 and S15 Table), genes that are highly expressed in ENCDCs at all timepoints and which are well known genes that influence ENS development [6,30–33]. Identification of genes expressed in fetal gut reduced the total number of candidate modifier genes to 732 (Table 4).

**Table 4:** Number of genes within each modifier interval subsetting those that are expressed and differentially expressed in scRNA-seq of the fetal gut.

		Modifier Interval Sets						
		BothSexAllChrom	BothSexNo15X5	BinBothSexAllChrom	Male	Female	BinMale	BinFemale
**Gene Prioritization in modifier intervals by scRNA-seq of fetal gut**	**UCSC mm10 Genome**	1212	875	2040	1439	1379	2889	1607
	**scRNA-seq: Log-Normalized Expression > 1**	119	72	253	127	105	383	161
	**scRNA-seq: Differentially Expressed (Cell Type + Developmental Timepoint)**	110	64	239	113	99	362	155

This table displays several levels of prioritization of genes within the *Sox10*^*Dom*^ aganglionosis modifier intervals. First row - genes present within modifier intervals identified on the USCS table browser of build mm10. Second row whether the genes from row 1 are expressed above a log-normalized expression threshold of 1 in pseudo-bulk of cell types at specific developmental time points. Third whether expressed genes exhibit differentially expression across either cell type or developmental stages. Columns indicate the GEMMA association run that produced the modifier intervals containing these genes.

We similarly evaluated expression of genes located nearest the top two female associated SNPs from the sex-specific GWAS. The top female associated SNP had a LOD score of >3, while the second most significant female SNP had a higher LOD score than other modifiers that were replicated from the F_2_ study [20]. The closest genes to these SNPs include *Mcm3*, *Il17f*, and *Isl1*, of which *Mcm3* and *Isl1* are expressed in ENCDCs (Figs 5C and S4).

Identification of ENCDC expressed genes allowed us to assess whether pairs of genes (ligands – receptors) that could participate in cell-cell communication during migratory stages are present in modifier intervals. Communication between ENCDCs and the surrounding gut mesenchyme is an essential aspect for normal colonization of the developing fetal gut [34]. To evaluate the potential of candidate genes in modifier intervals to either signal from the gut mesenchyme to ENCDCs or affect communication from ENCDCs to the surrounding gut microenvironment during bowel colonization, we applied CellChat. CellChat is a computational tool that quantifies communication between two cell groups using a reference database of over 3300 ligand receptor interactions and reliance on random permutation to estimate ligand-receptor communication among cell groups in scRNA-seq datasets [35,36]. Utilizing the wildtype scRNA-seq dataset [24], CellChat analysis identified predicted ligand-receptor communication between ENCDCs and other cell types in the wildtype scRNA-seq dataset for five genes present in modifier intervals (S5A Fig and S16 Table). This analysis points to biological relevance of *Ppia*, *Col1a1*, *Lgals9*, *Ednrb*, and *Nrg1* for communication between ENCDCs and other gut cells during migration along the bowel. These genes are within nominally significant modifier intervals that are not significant after multiple testing correction. However, these genes do fall within nominally significant intervals that were detected in the prior F_1_-intercross study [20]. CellChat-identified modifier interval genes and the associated binding partner show at least one of each are expressed in ENCDCs in the Zhou scRNA-seq dataset (S5B Fig). Several of the genes present within modifier intervals that CellChat identifies include genes that are either associated with ENS development (*Ednrb*) or are known risk genes for HSCR like *Nrg1* [37]. Our analysis found that while single genes from a ligand-receptor pair were present within modifier intervals no instances were observed where both genes for a ligand and receptor pair resided in modifier intervals. Taken together, the predicted cell communication pathways detected for these modifier interval genes adds another prioritization filter based on the potential to influence ENCDC-fetal gut environment communication during bowel colonization.

### Differential expression of modifier interval genes in the migrating wavefront of enteric neural crest-derived cells further prioritizes candidate genes

**Fig 6:**
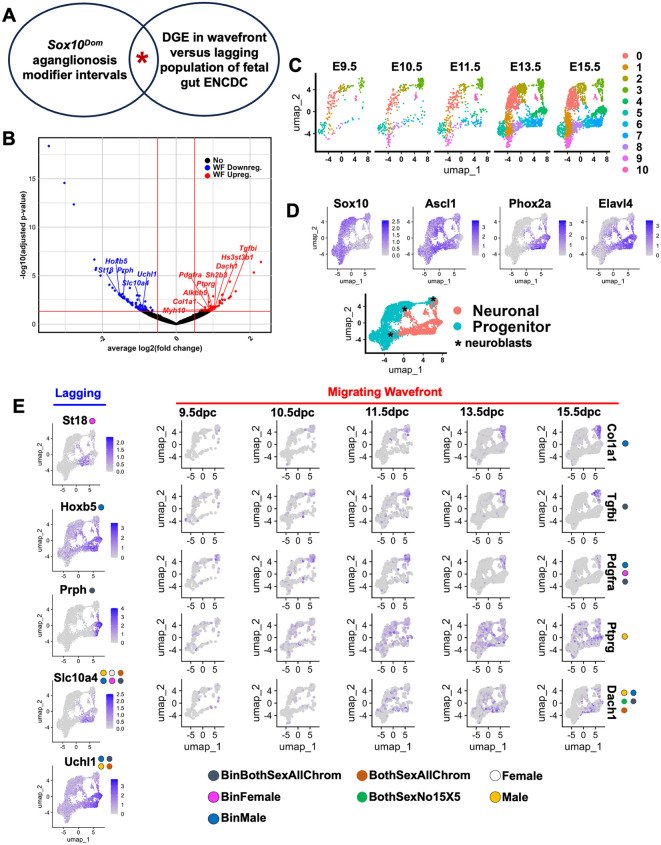
Overlap between differentially expressed genes at the enteric neural crest-derived migrating wavefront and genes within modifier intervals identifies migratory candidate genes. **(A)** Venn diagram visualizing pipeline for prioritizing *Sox10*^*Dom*^ aganglionosis modifier interval genes by differential gene expression at the migrating wavefront. Red asterisk indicates overlap between modifier intervals and upregulated genes in the migrating wavefront. **(B)** Volcano plot of all differentially expressed genes of the migrating wavefront enteric neural crest-derived cells at 11.5dpc versus the lagging/stationary cells. Red and blue dots indicate genes upregulated and downregulated in the wavefront, respectively. Genes labeled are those within modifier intervals. **(C)** UMAP of scRNA-seq from [24] enteric neural crest-derived cells (ENCDCs) based on source data annotations isolated from the main dataset and split by timepoint. **(D)** Expression of *Sox10*, labels progenitors (cyan) while *Elavl4* and *Phox2a* expression labels neuronal cells (salmon). Asterisks mark transitional neuroblast populations that express *Aschl* and *Sox10*. **(E)** Feature plots of [24] ENCDCs colored by expression of genes differentially downregulated (left) and genes differentially upregulated (right) in the migrating wavefront. UMAPs visualizing expression genes differentially downregulated in the wavefront are not split by time point while UMAPs for genes differentially upregulated are split by time. Dots next to gene names indicate from which modifier interval set each gene falls within.

Colonization of the fetal intestine by ENCDCs relies heavily on migratory capacity of the leading progenitor cells at the advancing edge of the migrating wavefront [38–42]. At 11.5dpc, ENCDCs are transitioning around and across the midgut fold to colonize the hindgut. Prior bulk-RNA seq profiling of leading-edge cells compared to the residual cells further back in this migrating population found these leading wavefront cells exhibit distinct transcriptional features [9]. Because migration is an essential process for hindgut colonization and these leading-edge progenitors exhibit a promigratory expression profile that may be relevant for modifying the migration defects of *Sox10*^*Dom*^ mutants, we filtered for genes within modifier intervals that were differentially expressed in the ENCDC wavefront versus lagging cells (Fig 6A). We overlapped those differentially expressed genes with the genes in our modifier intervals to identify any genes that exhibited differential expression either up- or down-regulated in the leading-edge cells versus cells further back in the migrating population. Of the 732 candidate modifier genes, five genes were downregulated (*St18*, *Hoxb5*, *Prph*, *Slc10a4*, and *Uchl1*) and 9 genes were upregulated (*Myh10*, *Col1a1*, *Alkbh5*, *Pdgfra*, *Ptprg*, *Sh2b3*, *Dach1*, *Hs3st3b1*, and *Tgfbi*) in the migrating wavefront (Fig 6B and S17 Table). Several of these genes including *Hoxb5*, *Pdgfra*, *Ptprg*, *Alkbh5*, *Dach1*, and *Tgfbi* have been implicated in various aspects of neural crest development [43–50].

To validate expression in a separate dataset and determine if these differentially expressed genes at the migrating ENCDC wavefront are expressed in specific groups of cells within the ENCDC population, we subset the whole gut developmental scRNA-seq dataset to focus just on ENCDCs (Fig 6B and 6C). Expression of *Sox10* and *Phox2a* identify progenitors and neuronal cells, respectively (Fig 6D). We expected modifier candidate genes upregulated in the migrating wavefront of the ENCDCs might be expressed in progenitor cells which are highly migratory, while downregulated genes would be expressed in more mature neuronal ENCDC cells (Fig 6B and 6D). Downregulated genes in the ENCDC wavefront like *Uchl1* and *Prph* are most highly expressed in more mature, neuronal populations (Fig 6E—**Lagging**). However, wavefront upregulated genes are expressed in either an intermediate population (*Col1a1*, *Tgfbi*, *Pdgfra*) which also expresses *Ascl1*, a known marked of enteric neuroblasts, or increase in expression over time (*Ptprg*, *Dach1*; Fig 6E —**Migrating Wavefront**). Mapping expression for these candidate genes in the scRNA-seq across early and later migratory stages provides insight into ENCDC cell type and temporal-specific expression.

### *Evolutionarily conserved* SOX10 *binding sites within Sox10*^*Dom*^ *modifier intervals overlap or are near genes differentially expressed in the migrating wavefront*

To further distill relevant candidate genes within *Sox10*^*Dom*^ aganglionosis modifier intervals potentially regulated by SOX10 DNA binding, we leveraged a dataset of dimeric SOX10 binding motifs conserved across chick, mouse, and human genomes [51]. We located 24 conserved SOX10 binding motifs within modifier intervals (Tables 5 and S18). We located the closest gene to each conserved SOX10 binding motif, yielding 17 unique genes, some of which are nearby multiple SOX10 binding motifs (Table 5). Most of these binding motifs were intronic to the annotated gene with only one motif positioned outside the nearest gene (~1.3kb away from *Sox2*; Table 5). Each 1Mb region surrounding the modifier interval-contained SOX10 binding motifs was manually examined on the mm10 genome to determine whether there were nearby genes that were already identified via other data modalities or already known to affect ENS development [22]. This added 6 genes to this list (Table 5). Several of these genes have already been identified in this work by alternate means, including *Mcm3*, *St18*, *Pdgfra*, *Ptprg*, and *Dach1* (Table 5). Also identified are several genes that have two or more intronic or close conserved SOX10 binding motifs including *Dach1*, *Bcas3* (close to *Tbx2*), *Tenm2*, *Ranbp17* (close to *Tlx3*), and *Mecom* with four intronic motifs (Table 5). Lastly, other genes not previously identified through other omics were near or overlap with conserved SOX10 binding motifs within modifier intervals, including *Bai3* (*Adgrb3*), *Cyp7b1*, *Nlg1*, *Slit2*, *Ppargc1a*, *Prdm8* (close to *Antxr2*), *Fam204a*, and *Sox2* (Table 5). Several of these genes are expressed in the ENCDCs (Figs 6 and S6; [24]). Altogether, these 24 conserved SOX10 binding motifs within modifier intervals highlight potential targets of SOX10 binding that could influence the aganglionosis phenotype caused by the *Sox10*^*Dom*^ mutation.

**Table 5:** Conserved SOX10 TF binding sites within *Sox10*^*Dom*^ aganglionosis modifier intervals.

SOX10 Binding Motifs within modifier intervals	Nearest gene to Conserved SOX10 Binding Motif within Modifier Intervals (Distance in bp)	Modifier Interval(s) Containing SOX10 Binding Motifs	Candidate Gene Near Conserved SOX10 Binding Motif within Modifier Intervals (Distance in bp)
chr1:6657718–6657735	*St18* (0)	BinFemale_Chrom1_Interval1	*St18* (0)
chr1:20107020–20107039	*Pkhd1* (0)	Female_Chrom1_Interval1 BinFemale_Chrom1_Interval1 BinBothSexAllChrom_Chrom1_Interval1 BothSexAllChrom_Chrom1_Interval1 BothSexNo15X5_Chrom1_Interval1	*Il17f* or *Mcm3* (670107 or 695929)
chr1:25112307–25112324	*Bai3/Adgrb3* (0)	Female_Chrom1_Interval1	*Bai3/Adgrb3* (0)
chr3:18205260–18205275	*Cyp7b1* (0)	Male_Chrom3_Interval1	*Cyp7b1* (0)
chr3:25512805–25512820	*Nlgn1* (0)	Male_Chrom3_Interval1 BothSexAllChrom_Chrom3_Interval1	*Nlgn1* (0)
chr3:30107333–30107347 chr3:30349666–30349684 chr3:30392542–30392558 chr3:30452770–30452789	*Mecom* (0)	Male_Chrom3_Interval1 BinMale_Chrom3_Interval1 BothSexAllChrom_Chrom3_Interval1 BothSexNo15X5_Chrom3_Interval1	*Mecom* (0)
chr3:34648638–34648655	*Sox2* (1349)	Male_Chrom3_Interval1	*Sox2* (0)
chr5:48179984–48179999	*Slit2* (0)	BinFemale_Chrom5_Interval1 BinBothSexAllChrom_Chrom5_Interval1	*Slit2* (0)
chr5:51536938–51536953	*Ppargc1a* (0)	BinMale_Chrom5_Interval2	*Ppargc1a* (0)
chr5:75011021–75011037	*Chic2* (0)	BinFemale_Chrom5_Interval3 BinMale_Chrom5_Interval2 BinBothSexAllChrom_Chrom5_Interval2	*Pdgfra* (141269)
chr5:98180845–98180859	*Prdm8* (0)	BinMale_Chrom5_Interval2	*Prdm8* or *Antxr2* (0 or 149883)
chr11:33343496–33343515 chr11:33400347–33400362	*Ranbp17* (0)	Male_Chrom11_Interval1	*Tlx3* (139907, 196758)
chr11:36031715–36031730 chr11:36561317–36561335	*Tenm2* (0)	Male_Chrom11_Interval1	*Tenm2* (0)
chr11:85431088–85431103 chr11:85713434–85713453	*Bcas3* (0)	BinMale_Chrom11_Interval1 BothSexNo15X5_Chrom11_Interval1	*Bcas3* or *Tbx2* (0 or 401448, 119098)
chr14:12476082–12476096	*Cadps* (0)	Male_Chrom14_Interval1	*Ptprg* (234041)
chr14:97840269–97840283 chr14:97891443–97891462	*Dach1* (0)	Male_Chrom14_Interval2 BinMale_Chrom14_Interval2 BinBothSexAllChrom_Chrom14_Interval1 BothSexAllChrom_Chrom14_Interval2 BothSexNo15X5_Chrom14_Interval1	*Dach1* (0)
chr19:60205701–60205717	*Fam204a* (0)	Female_Chrom19_Interval2	*Fam204a* (0)

The first column lists conserved SOX10 binding motifs grouped by both chromosome as well as the nearest gene, which is listed in the second column. All dimeric binding motif positions are in genome build mm10. The third column indicates which modifier intervals contain the conserved SOX10 binding motifs. The fourth column lists near genes that either have appeared already in prior omics datasets from this study or are relevant for ENS development.

### Chromatin accessibility within enteric neuronal progenitors highlights putative regulatory regions within aganglionosis modifier intervals

**Fig 7:**
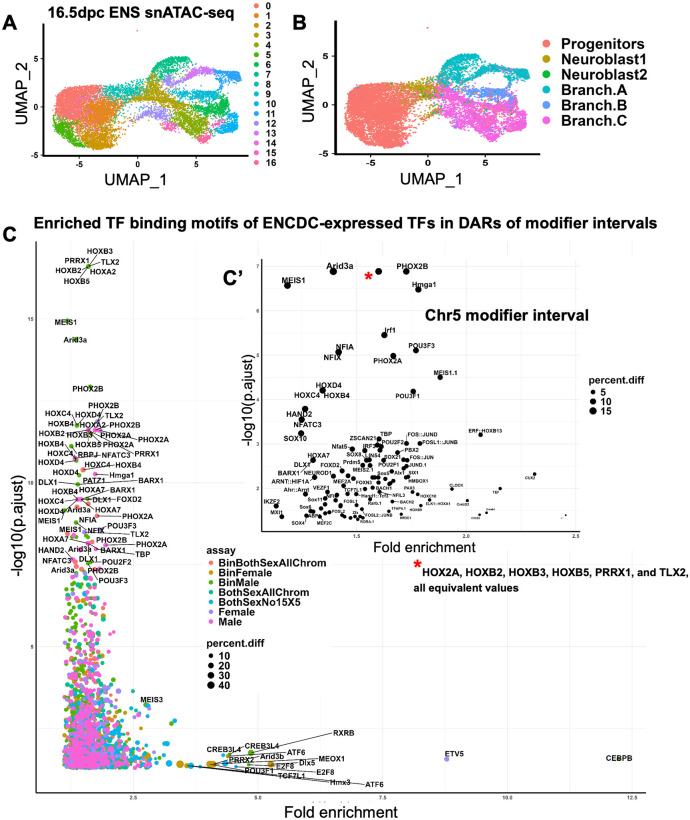
Whole gut 16.5dpc *Phox2b* H2B-CFP + snATAC-seq-derived transcription factor binding motif enrichment in differentially accessible chromatin identifies modifier interval-contained enriched TF motifs. **(A)** UMAP of snATAC-seq nuclei colored by unsupervised clusters. **(B)** UMAP displaying the result of label transfer of cell types from scRNA-seq to snATAC-seq. **(C)** Scatterplot of enriched TF binding motifs from the modifier interval-contained differentially accessible chromatin from progenitor and neuroblast clusters filtered by expression of their corresponding genes in the fetal gut ENS. The scatterplot points are colored by which modifier interval set each TF motif is enriched, with duplicates resulting from the analysis being performed by specific interval (“assay”). (**C’**) Scatterplot of enriched TF binding motifs from panel C plotted only for the BothSexAllChrom Chr5 modifier interval. The percentage difference between the number of TF motifs in the DAR and the background open chromatin (“percent.diff”) is indicated by the size of the point.

Modifiers can consist of variants in *cis*-regulatory elements that affect gene expression [52,53]. To assay open chromatin, that might harbor sites of gene regulatory activity within *Sox10*^*Dom*^ modifier intervals, we utilized single nucleus assay for transposase-accessible chromatin with sequencing (snATAC-seq) within mouse fetal ENS cells (S7A Fig). We captured ENCDCs at 16.5dpc based on expression of a *Phox2b* H2B-CFP transgene shown to mark glial, neuronal progenitor, and neuronal cells [54,55]. By 16.5dpc, the gut has been fully colonized, yet enteric neurogenesis is ongoing. We processed 13431 *Phox2b* H2B-CFP+ nuclei and performed unsupervised clustering to obtain 17 clusters (Fig 7A). We then utilized a pre-existing scRNA-seq dataset flow-sorted from WT 15.5dpc ENS to annotate snATAC-seq nuclei via estimated gene expression (see Supplementary Methods; S7B Fig). This approach assigned six main groups (progenitors, neuroblast1 and 2, and neuronal branches A, B, and C; Figs 7B and S7C). Differential chromatin accessibility analysis was used to localize 71521 differentially accessible chromatin regions (DARs) for each of these six main groups (S19 Table). Genomic annotations did not deviate across groups except for Neuroblast2, which we postulate could be due to fewer nuclei assigned as compared to the other groups (n = 102 Neuroblast2 nuclei/ 13431 total nuclei; S7D Fig, top panel). We filtered these DAR to those within *Sox10*^*Dom*^ modifier intervals, yielding 9151 unique regions (S20 Table). Modifier interval DAR regions in the Neuroblast2 nuclei are limited to promoter and distal intergenic categories, and this might be, again, due to fewer nuclei assigned to this group (S7D Fig, bottom panel).

To determine whether conserved SOX10 binding sites from Gopinath and colleagues localized to DAR regions within modifier intervals, we examined the overlap between these datasets [51]. Two of the conserved SOX10 binding motifs overlapped with modifier interval DAR. These were intronic to *Ranbp17* (close to *Tlx3*; chr11:33400347–33400362) and *Dach1* (chr14:97891443–97891462; Tables 5 and S21).

Transcription factor (TF) binding to cis-regulatory elements is important for the transcription of genes, and prominent candidate genes identified in this study are TFs including *Phox2b*, that have established roles in enteric neurogenesis [23]. We performed TF binding motif enrichment on modifier interval-contained DAR from progenitor and neuroblast groups to evaluate potential regulators of ENS development in *Sox10*^*Dom*^ modifier intervals. We focused on data from the progenitors and neuroblasts groups because these cell states enable collection of sufficient numbers of nuclei to pursue accessibility studies that otherwise would have been extremely difficult with the low numbers of migrating ENCDCs at early stages of gut colonization. Our approach examined both open chromatin in progenitors and neuroblasts as well as regions that are closed in differentiating enteric neuronal cells. This resulted in 797 enriched TF binding motifs in modifier interval DAR compared to the rest of accessible chromatin (S22 Table). We filtered these TF binding motifs to select for those whose encoding genes are expressed in the ENCDC scRNA-seq data, revealing 367 motifs (S23 Table). This enrichment analysis coupled with expression identifies the following TFs from modifier intervals: *Phox2b*, *Rbpj*, *Hoxb2/3/4/5/9*, *Sox2*, *Pou5f1*, *Tlx2*, *Tbx3*, *Pou3f1*, and *Mecom* (Fig 7C and S23 Table). Several of these TFs participate in ENS development [56–61].

### Prioritization of top aganglionosis modifier candidate genes across data modalities

**Fig 8:**
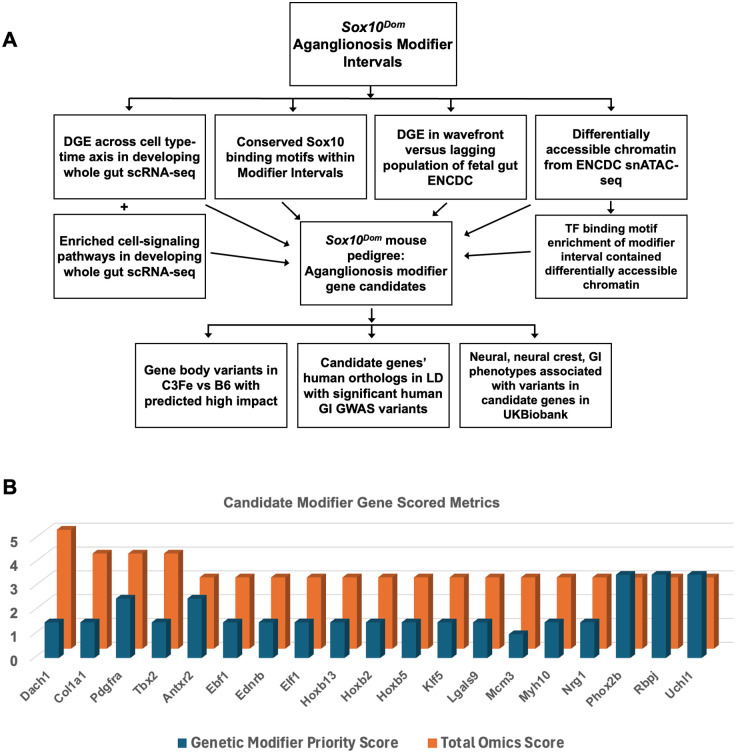
Analysis pipeline used to identify candidate genes from *Sox10*^*Dom*^ pedigree-derived aganglionosis modifier intervals. **(A)** Outline for candidate gene analysis pipeline following GEMMA genome-wide scans to identify genomic regions that modify the aganglionosis phenotype in *Sox10*^*Dom*^ mice, then use bulk and single cell sequencing strategies to identify candidate causal genes. After identifying candidate genes, these are then used to find gene body variants in C3Fe as compared to mm39 (C57BL/6J), then compared to human phenotype-genotype association data. DGE: differential gene expression. **(B)** Bar chart plotting the relative genetic modifier priority score and the total omics evidence score for 19 candidate genes with modifier prioritization scores >0 and multiple lines of omics evidence.

To rank candidate genes within *Sox10*^*Dom*^ aganglionosis modifier intervals (S13 Table) we assessed both genetic and multiomics data in a prioritization pipeline (Fig 8). In this process, we applied two scoring metrics: a modifier prioritization score and an omics evidence score. For our modifier interval prioritization score, we assigned 1 to 3.5 points to each modifier interval and genes within these intervals inherited this score based on the following criteria to minimize false positives: 1) peak SNPs of modifier intervals significant after multiple testing correction were assigned one point, 2) peak SNPs of modifier intervals with LOD score of ≥3 were assigned one point, and 3) intervals replicated from the prior F_1_-intercross study were assigned 1.5 points (Tables 6 and S24; [20]). We defined the preponderance of evidence omics score over a range of 0–5 corresponding to the number of separate lines of evidence implicating candidate genes. This incorporated single cell differential expression, estimated cell communication (CellChat), differential expression at the ENCDC wavefront, snATAC-seq-derived DAR regions in ENCDCs, ENCDC-expressed genes whose TF binding motifs are enriched within DAR regions in modifier intervals, and proximity to evolutionarily conserved SOX10 binding motifs (Fig 8A and S25 Table). Genes from each modifier interval were assessed independently through our prioritization pipeline to identify relevant candidates with potential to influence aganglionosis (Fig 8A and S25 Table). Thirty genes emerged that appear in three or more of our omics analyses. Of these, 11 with modifier interval prioritization scores of 0, indicating lack of strong genetic evidence, including *Mecom* and *Sox2*, were excluded from further analysis. Nineteen genes exhibited modifier prioritization scores >0 and multiple lines of omics evidence (Fig 8B and Table 6). One top candidate modifier gene, *Dach1*, exhibits five lives of omics evidence. Three additional genes have four supporting lines of evidence (*Col1a1*, *Pdgfra*, and *Tbx2*; Tables 6 and S25). Several genes that emerged from the pipeline are already known to be involved in ENS development and HSCR including *Phox2b*, *Ednrb*, and *Nrg1*, which effectively serve as internal controls.

**Fig 9:**
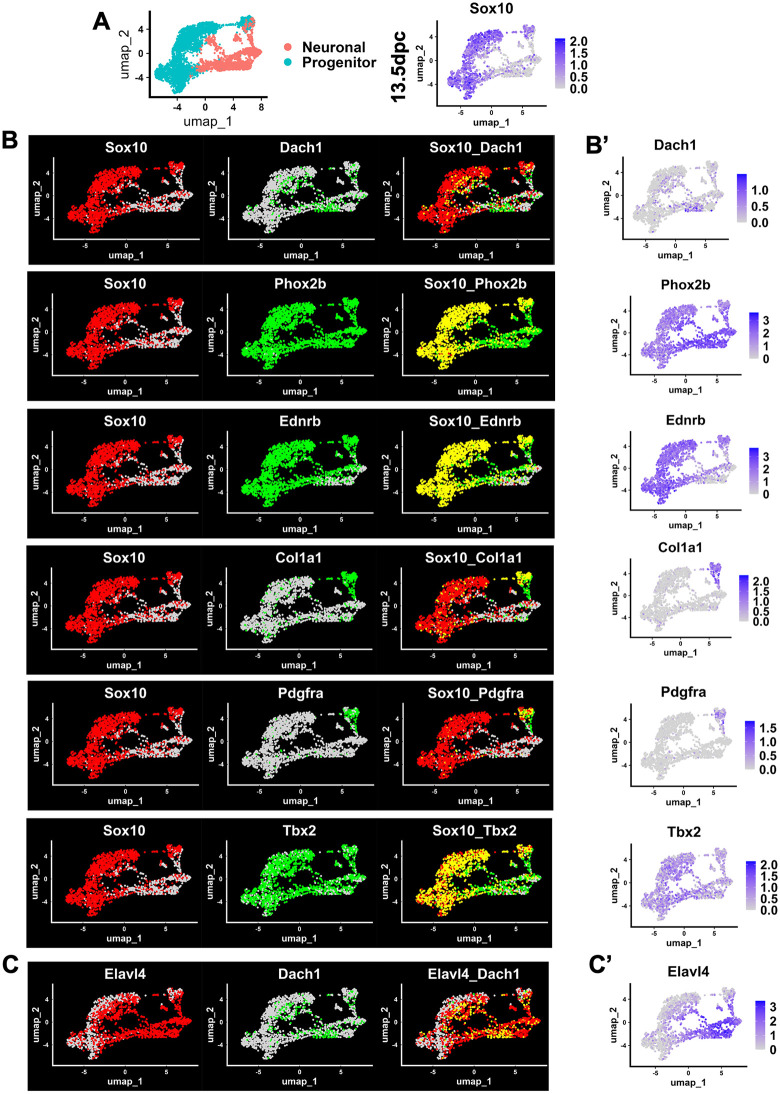
Co-expression of candidate modifier genes with Sox10 during migration stages highlights potential for interaction. Potential gene interaction within the same cells were examined in feature plots from the [24] data evaluated for co-incident transcription with *Sox10*. **(A)** All ENCDCs from Zhao et al. colored to show progenitor and neuronal states (left) and normalized expression of *Sox10* at 13.5dpc. Normalized expression **not** limited by “max.cutoff=0.5” is shown. **(B)** UMAPs showing co-expression (yellow) at 13.5dpc of top candidate genes (green) with an omics score of 4+ and well established markers of ENS subtypes (*Dach1, Phox2b, Ednrb, Elavl4, Pdgfra, Tbx2,* and *Col1a1*) relative to *Sox10* transcription in Zhao et. al. 2022 data. Feature plots show normalized expression limited by “max.cutoff=0.5” to highlight cells that may lowly express, but still express candidate genes. (**B’)** Normalized expression of candidate genes corresponding to those in B without a max cutoff of 0.5. **(C)** Co-expression of neuronal marker *Elavl4* (red) and candidate gene *Dach1* (green). Expression shown uses a max cutoff of 0.5 normalized expression to highlight cells that may lowly express, but still express *Dach1*. (**C’)** Normalized expression of *Elavl4* without a max cutoff of 0.5.

Given the transcription of several candidate modifier genes within ENCDCs and the presence of conserved SOX10 binding motifs nearby several of these candidates, we evaluated co-expression of genes with a priority score of four or greater with *Sox10* (Table 6)*.* We undertook this analysis in the subset ENCDC populations from the [24] data set during migratory stages (13.5 dpc) when the hindgut is being colonized. This effort identified co-transcription of *Sox10* with *Phox2b*, *Ednrb, Dach1*, and *Tbx2* in large numbers of ENCDCs (Fig 9). Similarly, *Ednrb* is expressed in progenitors and early neurons coincident with *Sox10* transcription. *Tbx2* and *Sox10* are also co-expressed among large numbers of progenitors and transitional neuroblasts. While *Dach1* has the highest omics score (5), the gene is most frequently transcribed in transitional neuroblasts and developing neurons (S8 Fig). This distribution results in the most frequent co-transcription of *Sox10-Dach1* occuring in transitional states between progenitors and neurons (Fig 9). In contrast, *Pdgfra* and *Col1a1* exhibited infrequent co-transcription with *Sox10* that was sparse among progenitors and observed primarily in a single transitional population. These patterns of co-expression suggest the potential for direct interaction between *Sox10* and several candidate modifer genes in ENCDC cells.

After comparison across data modalities to identify candidate genes with the highest relevance scores, we probed each candidate modifier gene sequence for C3Fe variants, as compared to B6, with predicted high impact on expression [62]. Of these candidate genes, *Phox2b* and *Col1a1* contained C3Fe variants with predicted high functional impact (S26 Table). *Phox2b* contained a C3Fe variant (T > TC insertion, chr5:67255011, transcript ENSMUST00000174251.2 UCSC Genome Browser, mm39) located at a splice junction boundary that could affect splicing efficiency and overall gene expression levels [22]. *Col1a1* also contained a C3Fe variant predicted to impact splicing (S26 Table). Identification of sequence variants between the C3Fe and B6 strains for *Phox2b* and *Col1a1* candidate genes adds further evidence to the likelihood that strain differences may exacerbate severity of *Sox10*^*Dom*^ aganglionosis.

### Candidate modifiers of aganglionosis related to human GI phenotypes

To relate our *Sox10*^*Dom*^ aganglionosis modifier candidate genes to human GI and HSCR disease loci, we assessed whether these genes’ human orthologs were in linkage disequilibrium (LD) with SNPs identified as significant from four human HSCR GWAS and GWAS of stool frequency [10–13,63]. Two separate HSCR GWAS summary statistics had significant SNPs that were in LD with genes identified in this study, including *Phox2b*, *Col1a1*, *Rbpj*, *Nrg1*, *Ednrb*, *Hoxb2/5/13*, and *Uchl1* (Tables 7 and S27–S29; [10,12]). The GWAS of stool frequency had significant SNPs that were in LD for *Col1a1*, *Rbpj*, *Nrg1*, and *Elf1* (S30 Table; [63]). While not in an LD block with significant SNPs, *DACH1* is ~ 2.6Mb away from an associated SNP in the stool frequency GWAS [63].

**Table 7:** Human genome-wide significant SNPs that are in LD with candidate gene orthologs identified in *Sox10*^*Dom*^ modifier analyses.

Mouse Candidate Gene	GWAS(s)	Number of SNPs in LD with Human Ortholog	Ancestry LD Block(s)	Distance, Range of Distances from gene to SNP(s) in base pairs
***Phox2b***	Tang HSCR	2	EUR, ASN	21281, 395969
***Col1a1***	Tang HSCR, Stool Freq	2	EUR, ASN	15888, 1568054
***Rbpj***	Garcia-Barcelo HSCR, Stool Freq	5	EUR, ASN	41792-60046, 269536
***Nrg1***	Garcia-Barcelo HSCR, Stool Freq	85	EUR, ASN	0-72775
***Ednrb***	Tang HSCR	2	EUR, ASN	0, 30159
***Hoxb5***	Tang HSCR	2	EUR, ASN	23438, 22300
***Uchl1***	Tang HSCR	2	EUR, ASN	79684, 501822
***Elf1***	Stool Freq	42	EUR	1255345-1387370
***Hoxb13***	Tang HSCR	2	EUR, ASN	107586, 108724
***Hoxb2***	Tang HSCR	2	EUR, ASN	71010, 72148

Each candidate gene was evaluated for whether they were within shared LD blocks with significant GWAS SNPs. The third column indicates from which human GWAS(s) significant SNPs that share an LD block with the candidate genes were sourced, including Garcia-Barcelo et al. [10] (Garcia-Barcelo HSCR), Tang et al. [12] (Tang HSCR), and Bonfiglio et al. [63] (Stool Freq). The fourth column indicates how many SNPs are in LD with the given candidate gene’s human ortholog. The fifth column indicates whether the gene is within either European (EUR) or Asian (ASN) ancestry LD blocks with significant GWAS SNPs. The last column indicates the distance or range of distances in base pairs from the gene to a SNP(s).

To further relate our candidate *Sox10*^*Dom*^ aganglionosis modifier genes to other human GI, neural, and neural crest phenotypes, we took an exploratory approach to probe the publicly available exome-based PheWAS sever Genebass. We evaluated whether variants in human orthologs of our candidate mouse genes might be associated with ENS-related traits. We utilized a text/term-based search among the top 20 phenotypes that were returned for each of the orthologs (Supplemental Methods; [64,65]). Of the orthologs examined in PheWAS, each had phenotypes based on partial string searches that captured terms based on neural crest, nervous system, or GI (Supplemental Methods). Among these is *DACH1* with four different phenotypes relating to GI surgery (S31 Table). Interestingly, among the terms found for *DACH1* is “Anterior resection of rectum and exteriorisation of bowel”, a process often used to treat HSCR.

## Discussion

While HSCR patient studies have largely focused on causal genes, animal models, such as *Sox10*^*Dom*^, are advantageous for defining the role of genetic background on aganglionosis severity. Prior *Sox10*^*Dom*^ modifier mapping studies identified very large genomic intervals and relatively few genes emerged as modifiers of aganglionosis severity and penetrance [20]. Omics from relevant cell and tissue types can help prioritize large numbers of genes in modifier intervals to select candidates with highest potential to influence a trait. In this study, we conducted genome-wide analyses on an extended pedigree of the *Sox10*^*Dom*^ mouse model of HSCR to refine aganglionosis modifier intervals and identify candidate modifier genes within those intervals. Several nominally significant modifier intervals are associated in a sex-biased manner. Utilizing multiple omics datasets including scRNA-seq, bulk RNA-seq, snATAC-seq, and TF binding motifs, we analyzed genes within modifier intervals to prioritize those candidate genes with the greatest relevance for aganglionosis. This strategy revealed multiple highly relevant candidate modifier genes that are expressed in developing gut, and which either have known roles in ENS development or exert effects on other aspects of cell migration. Our analysis confirms the interval near *Phox2b* is a candidate modifier of *Sox10*^*Dom*^ aganglionosis and identifies multiple novel candidate genes likely to influence aganglionosis severity. Among these, *Dach1* stands out as the highest priority candidate based on omics data and prior evidence of this gene affecting cell migration [47,66,67].

Quantitative trait mapping in mice has blossomed with the availability of mouse genetic resources like the collaborative cross [68,69]. However, challenges remain for identifying disease modifiers that rely on inclusion of a mutant allele. We took advantage of a multi-generational *Sox10*^*Dom*^ mouse pedigree for GEMMA genome-wide analyses anticipating that the iterative crosses of *Sox10*^*Dom*^ males with B6C3Fe-*a*/*a* wildtype females would greatly narrow the associated genome regions. This approach compliments our previous F_1_-intercross strategy to map *Sox10*^*Dom*^ aganglionosis modifiers by taking advantage of additional recombination in pedigree generations. Aganglionosis modifiers identified in this study replicate intervals located in a prior F_1_-intercross including a genome-wide significant modifier on chromosome 5 and marginally significant modifiers on chromosomes 3 (shifted), 8, 11, and 14 [20]. We also detect novel marginally significant modifiers, including some that are sex-biased (Tables 1 and 2). However, the modifier intervals we detected were still quite large, some exceeding 10Mb. The large size of modifier intervals produced in aggregate thousands of candidate genes. This result necessitated that we develop strategies to prioritize and filter for candidate genes that are most likely to be influencing severity of aganglionosis. This multistep process, moving from genome interval to a limited number of high priority candidate genes, relies upon accumulation of evidence to pinpoint those genes that are most likely to be influencing phenotype and is a prerequisite for further functional testing of individual genes [70].

Our dual score approach—confidence of modifiers and lines of evidence through omics analysis—allowed us to prioritize candidate genes from large genomic intervals. Among the total 6216 annotated genes in modifier intervals, 683 were expressed in either ENCDCs or the surrounding gut mesenchyme through which these cells migrate [24]. Subsequent gene filtering identified 5 cell communication candidate genes, 26 candidates near conserved SOX10 binding motifs, 9151 DAR regions enriched for motifs of 367 ENCDC-expressed TFs from snATAC-seq, and 14 genes differentially expressed at the ENCDC migrating wavefront. Thirty candidate genes with cumulative evidence aggregated from multiple modalities emerged as putative modifiers of *Sox10*^*Dom*^ aganglionosis, however only 19 of these also exhibited significant modifier scores >0 (Table 6). Of the 19 candidate genes, *Phox2b*, *Rbpj*, and *Uchl1* were located in modifier intervals that 1) had peak SNPs that were significant past the threshold for multiple comparisons, 2) had peak SNPs that had LOD scores ≥3, or 3) overlapped with intervals derived from the prior F_1_-intercross study (Tables 6, S24 and S25; [20]). *Pdgfra* and *Antxr2* met two of these criteria (Tables 6, S24 and S25). *Dach1* was within a replicated modifier interval (Tables 6, S24 and S25; [20]). We then examined these candidate genes for variants of predicted high impact in the C3Fe genome versus C57BL/6J. This analysis revealed potential function-altering C3Fe variants for *Phox2b* and *Col1a1* that could affect splicing [62].

Our analysis was challenging due to the zero-inflated, non-normal distribution of the phenotype resulting from approximately thirty percent of animals in the pedigree that exhibited no detectable aganglionosis. This zero-inflated phenotype required that, in addition to treating the phenotype as-is, we implement a binary phenotype conversion approach. We considered several alternatives including a rank-based transformation. Transformation of zero-inflated data would break the transformation assumption by collapsing all the zeros into a single rank or small rank set, which would ignore the structure of the trait distribution and could lead to spurious associations. We also considered analysis approaches based on converting the trait to a categorical classification (e.g., none, short, intermediate, long), which would also produce loss of biological variation like the rank-based transformation. Additionally, categorical classification would require imposing cutoffs for each category, which could bias the results. We were unable to locate an appropriate tool that could be utilized specifically for mouse pedigrees and would account for the inherent relatedness of inbred strains as GEMMA does while also accounting for the zero-inflated, non-normal distribution of the aganglionosis phenotype [71]. Therefore, we proceeded with GEMMA analysis despite the complication due to zero-inflation and 1) compensated with treating the trait as a binary phenotype in addition to quantitative and 2) assessed how the resulting associations were significant or marginally significant relative to the prior [20] study. This approach allowed us to identify modifier intervals that were replicated across studies. The presence of zero-inflated phenotypes remains a major challenge in quantitative traits analysis that would benefit from future methods/software package development capable of also dealing with strain relatedness and pedigree structures.

The mouse aganglionosis modifier candidate genes identified here may have relevance as modifiers of human GI disease. Therefore, we compared the mouse *Sox10*^*Dom*^ aganglionosis candidate genes to significant SNPs identified from HSCR and stool frequency GWAS [10–13,63]. Of the candidate genes, nine of these genes were in LD blocks with significant HSCR GWAS SNPs and four were in LD blocks with significant stool frequency GWAS SNPs (Table 7). In addition, we use exome-based PheWAS study results from the UKBiobank (Genebass) to find GI, neuronal, and neural crest phenotypes associated with variants in our candidate genes [64,65]. These comparisons suggest that our candidate genes in *Sox10*^*Dom*^ aganglionosis modifier intervals are likely relevant for human HSCR phenotype variation or other GI phenotypes.

In this study, we generated and mined a novel snATAC-seq dataset of 16.5dpc fetal ENS collected from *Phox2b* H2B-CFP mice [54, 55]. We used this dataset to prioritize modifier interval candidate genes based upon differentially accessible chromatin and enriched TF binding motifs. Because this dataset contains chromatin accessibility for populations that include progenitors, neuroblasts, and maturing neuronal linages, it has the potential to be highly informative for researchers interested in regulatory regions that control neuronal diversification and maturation in the fetal ENS. This dataset can be used further for analyses of TF binding motif accessibility changes over neurogenesis for each neuronal trajectory relative to analogous scRNA-seq datasets. Future work combining the snATAC-seq data with single cell gene expression profiles at 16.5dpc has the potential to identify putative target genes of predicted cis-regulatory elements that could emerge from combined snATAC-seq/scRNA-seq analytics. These may be relevant to HSCR or other gastrointestinal motility disorders that arise from disrupted ENS development.

Several of the high priority candidate modifier genes, identified here are co-transcribed with *Sox10,* which offers a means for direct interaction between genes*.* Prior genetic studies suggested the potential for direct interaction between *Sox10 and Phox2b* as well as *Ednrb* [6,20]. Our analysis reveals co-transcription of *Phox2b* with *Sox10* in progenitors and enteric glia as well as in early neuronal transitional states and is consistent with prior studies reporting *Phox2b* transcription in these cell types, [54,72]. Similarly, co-transcription of *Ednrb* occurs with *Sox10* in ENS progenitors and early developing neurons. The novel candidate modifiers identified by our omics analysis, including *Dach1*, *Col1a1*, *Tbx2*, and *Pdgfra*, are expressed in ENCDCs and exhibit intriguing patterns of expression across ENCDC populations. *Dach1* expression is observed in progenitors and in early transitioning neurons, where high levels of *Sox10* transcript are prevalent. Moreover, recent identification of SOX10 protein persistence in developing neurons further extends the potential timeline for interaction between SOX10 and the *Dach1* locus [61]. Co-expression of *Dach1* in *Sox10* + cells during critical migratory stages (13.5dpc) when the hindgut is being colonized has potential relevance for extent of aganglionosis. TBX2 has been reported in postnatal neurons of the ENS [60], although to our knowledge there have not been reports of TBX2 protein or transcription in fetal ENS other than current scRNA-Seq data sets. Our analysis identifies high levels of *Tbx2* in numerous ENCDC progenitors and early transitioning neuronal states with frequent co-transcription with *Sox10* (Fig 9). *Tbx2* exhibits several of the same features as *Dach1* with differential expression over ENS development, conserved SOX10 TFBMs, and differential chromatin accessibility between developing neurons and glia although it does not have the same wavefront differential expression that *Dach1* does. In contrast, *Pdgfra* and *Col1a1* exhibit very sparse co-expression with *Sox10* that is mostly limited to one of the several transitional neuronal states. Whether there is direct interaction between *Sox10* and these candidate modifier genes identified in our analysis awaits further experimental study.

**Fig 10:**
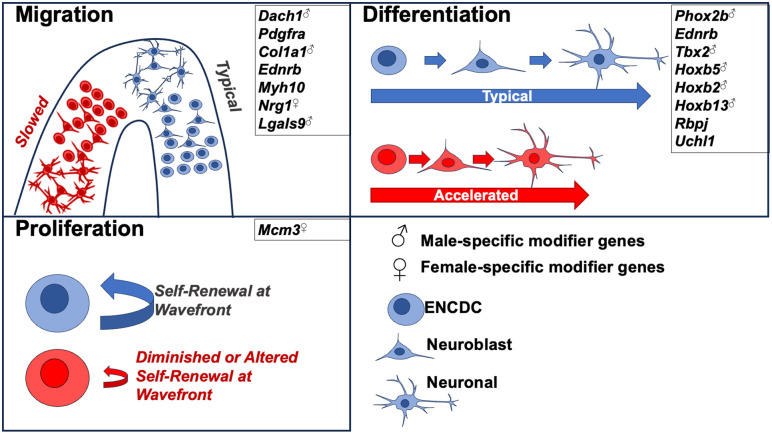
Models illustrating developmental processes by which candidate *Sox10*^*Dom*^ modifier interval genes may alter ENCDC migration. Schematic diagrams displaying migration of enteric neural crest-derived cells (ENCDCs), differentiation of ENCDCs towards more mature ENS fates, and proliferation of ENCDCs at the migrating wavefront. In blue are the typical functions, and in red are cells and functions that are the hypothesized results of perturbation of the candidate genes. Candidate genes hypothesized to be involved in each function are in smaller boxes in the upper right of each function box, with superscript sex symbols for those genes that are within sex-specific modifier intervals.

There is tremendous temporal and spatial complexity in ENCDC colonization that hinges upon migration, proliferation, and differentiation. Given that HSCR aganglionosis is a consequence of failed colonization, we hypothesize these top modifier candidates regulate different aspects of ENCDC migration (Fig 10). There is substantial prior evidence of *Dach1* influencing migration in neural crest, embryonic fibroblasts, and breast cancer [47,66,67]. In *Xenopus*, *Dach1* is specifically associated with early neural crest migration [47]. Similarly, *Col1a1* in zebrafish is implicated downstream of retinoic acid (RA) signaling, which has been shown to be important for ENS development and ENCDC migration [73–77]. *Pdgfra* affects both migration and survival in oligodendrocytes as regulated by *Sox9* and *Sox10* while PDGFR signaling has been shown to affect nitrergic enteric neuron specification *in vitro* [78,79]. In zebrafish, *tbx2a/b* CRISPR/Cas9 targeting caused reduced ENS cell density, suggesting function for differentiation or proliferation of ENCDCs [80]. Two genes that fell off our omics candidate list due to lack of strong genetic evidence include *Sox2* and *Mecom*. However, these two genes exhibit four lines of omics evidence. *Mecom* affects neural crest-derived chondrocyte differentiation, orientation, and polarity in mouse and zebrafish models [81]. *Sox2* has been shown to contribute to maintenance of progenitor states of neuronal progenitors and has been identified as a modifier of human HSCR via copy number variation [14,82]. These genes may exert small additive effects on *Sox10*^*Dom*^ aganglionosis despite their failure to reach significance in our genetic analysis*.* The depth of prior analyses assist in understanding how these different genes might contribute to the aganglionosis phenotype in *Sox10*^*Dom*^ mice.

There are several limitations to our study. The omics data analyzed in this study are sourced from the fetal gut and therefore does not include genes whose expression during initial migration of vagal neural crest from neural tube to the foregut, which also could influence *Sox10*^*Dom*^ aganglionosis. Ideally future analysis will capture expression profiles of early migrating vagal neural crest and deeply sequence migration stages to capture lowly expressed transcription factors. We utilized CellChat, which estimates ligand-receptor interactions, and misses any sort of transcription factor-chromatin interaction. There is also inherent stochasticity of ENCDC development even in congenic inbred backgrounds which can contribute to variation in aganglionosis [83]. Resolution for detecting modifiers on chrX is limited both due to the cross structure and the analysis tools we applied. GEMMA does not include algorithms that can account for copy number or mosaicism that are needed for analysis of chrX SNPs [21]. We expect since only male *Sox10*^*Dom*^ mice are bred to propagate the pedigree, and WT B6.C3Fe F_1_ female mice are introduced into the pedigree at each generation, there is minimal recombination on chrX. Even with the low density of SNPs in the genotyping panel and the high variability of aganglionosis phenotype, we replicated the chr5 interval containing *Phox2b*, which surpassed genome-wide significance after multiple testing correction. Other loci were nominally significant, including a novel locus on chr1 with a LOD score > 3 (rs6404446, chr1:20784226; Fig 4A and Table 3), and several other loci that replicated prior modifier regions [20]. Candidate genes in these regions were supported by our omics analysis. Lastly, future work validating the effects of individual modifier genes identified here, particularly *Dach1* and *Tbx2*, will be required. Analysis of individual modifier gene loss on ENCDP migration or the extent of aganglionosis will require complementation test crosses with *Sox10*^*Dom*^ mutants or knockdown studies in homogenous genetic backgrounds.

In conclusion, we demonstrate the ability to improve resolution of *Sox10*^*Dom/+*^ aganglionosis modifier intervals from mapping in an extended pedigree. Combinatorial omics analysis identifies a high priority list of modifier genes that meet multiple genetic and genomic metrics. Detection of *Phox2b* as a well-known gene in ENS development confirms the pipeline approach. *Dach1* emerges as a novel modifier of *Sox10*^*Dom*^ aganglionosis. Cumulative evidence for *Dach1* includes genetic interval replication across studies, prior reports of *Dach1* mutation on neural crest migration, and omics evidence of SOX10 binding sites in accessible chromatin around this gene. Our analysis enriches the gene network that impacts ENS development and reveals important genes for future variant analyses in human HSCR and other GI motility disorders.

## Methods

### Ethics statement

All animal experimental protocols were approved by the Institutional Animal Care and Use Committee (IACUC) at Vanderbilt University Medical Center.

### Mouse husbandry

All mice were housed in a modified barrier facility on a 14-hour on, 10-hour off-light cycle in high-density caging (Lab Products Inc., #10025) with breeders and all *Sox10*^*Dom*^ mice on (LabDiet 5LJ5) and water ad libitum. The B6C3Fe-a/a.*Sox10*^*Dom*^ line originated at Jackson Lab (Jackson Stock 000290) and was maintained by crosses to B6C3Fe-a/a WT female mice generated on site by crosses of C3FeLe.B6-*a*/J (Stock # 000198) females crossed to C57BL/6J (Stock # 000664) males. Pedigree offspring over 10 generations were euthanized at postnatal days 7–10 for GI tract collection that was dissected intact from stomach to anus.

The Tg(Phox2b-HIST2H2BE/Cerulean)1Sout mice (MGI: 5013571), *Phox2b* H2B-CFP, was maintained by crosses with female C3FeB6F1 [54]. Timed matings between female C3FeB6F1 and male *Phox2b* H2B-CFP mice were conducted to produce fetal intestines at 16.5dpc used for snATAC-seq.

### Acetylcholinesterase histochemistry

Whole-mount acetylcholinesterase enzyme histochemistry of all *Sox10*^*Dom*^ mutant offspring from the pedigree collected for this study was performed as described [6,84]. Briefly, intestines were dissected from postnatal 7–10 day old pups extending from the pyloric sphincter to the anus and gently flushed with 1X PBS to remove luminal contents. Tissues were fixed in 4% paraformaldehyde between 90–120 minutes on ice then rinsed twice before transfer to saturated sodium sulfate solution for an overnight incubation at 4^o^C. Tissues were stained with a solution of 0.2mM Ethopropazine, 4mM Acetylthiocholine Iodide, 10mM Glycine, 3mM Cupric Sulfate pentahydrate, 6mM Sodium Acetate Trihydrate to reveal patterning of the enteric ganglia within the gut wall as previously illustrated [6]. Total gut length and extent of aganglionosis was measured on a ruler to 1mm increments using a Leica MZ12.5 microscope to view ganglia distribution. All samples were scored independently by two researchers. The percent gut length aganglionic was calculated as a percentage of gut length lacking enteric ganglia over the total gut length to account for differences in body size due to differences in nutrition status of *Sox10*^*Dom*^ pups.

### Mouse genotyping

All pedigree offspring were genotyped for the *Sox10*^*Dom*^ allele using established methods [6].

Genome-wide SNP genotyping data was generated by the Center for Inherited Disease Research at Johns Hopkins University using the Illumina Mouse Linkage Panel (GoldenGate GS0006826-OPA), on a Sentrix array (1449 SNPs). Array data was analyzed with *de novo* clustering in GenomeStudio 2.0 with 1369 SNPs meeting quality metrics for analysis of which 876 were informative between C3HeBFeJLe-a/a and C57BL6/J.

### Analysis of *Sox10*^*Dom*^ extended pedigree using Genome-wide Efficient Mixed Model Analysis (GEMMA)

GEMMA was used generally on the population and in sex-specific analyses to generate relatedness matrices [21]. We then performed SNP-based aganglionosis association analyses using GEMMA. Analysis code and further details can be found in Supplemental Materials and in supplementary files located on our Zenodo repository.

### Modifier intervals for downstream candidate gene analysis

*Sox10*^*Dom*^ aganglionosis modifier intervals were defined as each interval of adjacent significant SNPs (p-value not adjusted for multiple tests) +/- 0.5 megabase (Mb). The + /-0.5Mb window was chosen to account for associations that may not contain the causal SNP(s), gene(s), or locus (loci) and is consistent with many long-range regulatory elements that are within a 1Mb interval of regulated genes (Fig 7). We utilize shorthand for each GEMMA association run. Shorthand for each GEMMA analysis and modifier interval set used as input for our analysis pipeline (Fig 7) is as follows: sex-regressed quantitative aganglionosis phenotype with zeroes included, **BothSexAllChrom**; sex-regressed quantitative aganglionosis phenotype with zeroes included with removal of SNPs from chromosomes 5, 15, and X, **BothSexNo15X5**; sex-regressed with pseudo case-control “binary” phenotype, **BinBothSexAllChrom**; male- and female-specific quantitative aganglionosis phenotype with zeroes included, **Male** and **Female**, respectively; male- and female-specific pseudo case-control “binary” phenotype, **BinMale** and **BinFemale**, respectively. Genes within modifier intervals were found using the UCSC Table Browser Tool [85] based on the above modifier interval genomic regions as input (S13 Table). Briefly, BED files were generated using the chromosome number, start, and end of the modifier intervals. These positions were input into the UCSC Table Browser, selecting for gene symbol output. Output gene lists were downloaded in.csv format and assessed for expression, then differential expression in scRNA-seq as described in the section below and our Supplemental Methods.

### Reprocessing of whole gut 9.5-15.5 days post coitus scRNA-seq data

ScRNA-seq data were downloaded from the Gene Expression Omnibus at accession GSE186525 [24]. Seurat and SCTransform version 2 were utilized for quality control, clustering, and integration across age and sample [86–88]. Seurat’s FindAllMarkers function was used for differential gene expression analysis. Analysis code and further details for scRNA-seq processing can be found in Supplemental materials and on our Zenodo repository.

### Enteric neural crest-derived cell wavefront differential gene expression (DGE) overlapping with modifier intervals

We downloaded differential gene expression data from Stavely et al. on the Gene Expression Omnibus at accession GSE217757. This file was imported into R and filtered by multiple testing correction-adjusted p-value of 0.05 and a log2FoldChange of <-0.5 and >0.5. These genes were then filtered by those that were within modifier intervals using the same strategy as performed in the methods for imprinted genes (see Supplemental information section “Determining whether modifier intervals contained imprinted genes”). This was done for each GEMMA association-based modifier intervals, both non-sex specific and sex specific.

### SOX10 conserved binding motifs within modifier intervals

SOX10 binding motifs from Gopinath et al. were downloaded and ported into UCSC Genome Browser’s LiftOver tool [51,89]. Motif regions were processed sequentially from hg18 to mm10 in LiftOver. The binding motifs were then assessed to determine which overlapped with modifier intervals from all GEMMA associations using R code.

### CellChat analysis of cell-cell communication in ENCDCs

We imported the reprocessed developing fetal gut scRNA-seq dataset/Seurat object from [24] into CellChat version 2.1.2 in R [35,36]. Per timepoint excluding 15.5dpc (when the gut is fully colonized with ENCDCs), we estimated ligand-receptor interactions between ENCDCs (“Neural crest”) and other cell types across the signaling genes expressed. We filtered then filtered to ligand-receptor interactions that passed interaction probability thresholds of 0.25 and were statistically significant. Of those that passed this filter, we subset those that were within modifier intervals.

### Generation and processing of fetal ENS single nucleus assay for transposase-accessible chromatin-sequencing (snATAC-seq) data

#### Isolation of nuclei from fetal mouse tissue.

At 16.5 days post coitus (dpc), C3FeB6.*Phox2b*-H2BCFP+ fetal intestine (from stomach to anus) was used as source tissue to isolate ENS cells relying on cold-active protease dissociation, as previously described [61]. Tissue was pooled and cells were isolated using fluorescence-activated cell sorting (FACS) to select for viable cells that excluded 7-aminoactinomycin D+ stain. Differential sorting for CFP+ high (neuronal cells) and separately CFP + low (progenitors, glial cells) was performed as reported [55]. Nuclei were produced from post-FACS cell suspensions using the 10x Genomics protocol CG000209_Rev D. Nuclei counts were obtained via hemocytometer or Countess Automated Cell Counter (Thermofisher) followed by encapsulation on the 10x Genomics Chromium Next GEM single cell platform. Libraries for snATAC-seq were produced from two replicates with the first CFP-high and -low sorted populations produced from 6 pooled fetal guts and the second produced from 12 pooled fetal guts. Libraries were sequenced on an Illumina NovaSeq 6000 using an S4 flow cell with custom read lengths to support ATAC-seq samples. Sequencing depth targeted greater than 70,000 paired-end reads per nucleus.

#### Analysis of snATAC-seq data.

Sequence FASTQs were processed with CellRanger ATAC pipeline version 1.2.0 by the Vanderbilt Technologies for advanced genomics (VANTAGE) shared resource [90]. Sequences were aligned to mm10. One CFP+ high-intensity sample was excluded based on CellRanger output showing an overabundance of nuclei with approximately 10 times fewer fragments per cell than other replicates (see supplementary methods). The remaining single CFP + -high and CFP + -low intensity samples were imported into R using Seurat and Signac. Default Signac processing was used to generate Seurat objects per replicate, including LSI dimensionality reduction [86,91; See Supplementary methods]. Harmony was used to integrate the two low-intensity replicates [92]. Seurat and Signac’s LabelTransfer was used to identify cell types according to scRNA-seq data from similar cell types at 15.5dpc (GSE262898), resulting in snATAC-seq counterpart clusters to the identities seen in scRNA-seq [61]. This approach included both enteric neuronal and glial lineages as genes marking both cell types are expressed at this stage [93]. Differential chromatin accessibility analysis was performed using MACS2-derived peaks and Seurat’s FindAllMarkers function for both unsupervised and LabelTransfer clusters [94]. The resulting differential chromatin accessibility datasets were filtered by Bonferroni-adjusted p-value < 0.05 and average log2 Fold Change > 1.5 and <-1.5. To find overlap for differentially accessible chromatin loci and *Sox10*^*Dom*^ aganglionosis modifier intervals, we first added 25 base pairs on either side of each differentially accessible locus, then assessed for overlap via the same method described in Supplementary Methods.

TFBM enrichment for differentially accessible loci within modifier intervals was performed using Signac’s built-in function “FindMotifs”. Briefly, the R package TFBSTools function “getMatrixSet” was used in combination with the JASPAR 2024 R package to get position frequency matrices (PFM) from each validated (core) vertebrate TFs [12, Rauluseviciute et al. 2024]. Since DACH1 binding motif’s PFM was not in the validated core, this PFM was manually added from the unvalidated section of JASPAR2024 [Rauluseviciute et al. 2024]. Signac’s “AddMotifs” function was used to add the motifs to the snATAC-seq Seurat object. Differentially accessible peaks for progenitor and neuroblast clusters, which were then split by each modifier interval per interval set (in other words, for each modifier interval set, the differentially accessible chromatin for each modifier interval were split, such as “Male_Chrom5_Interval1”). These were then input into the “FindMotifs” function to find enriched TF binding motifs per modifier interval. These were then filtered by TFs that are expressed via Seurat’s “AverageExpression” function greater than 0.1 (grouped by timepoint) in the [24] subset ENCDC (“Neural Crest”) scRNA-seq dataset [24]. The enriched TF binding motifs in modifier intervals were further filtered to those TFs whose genes were found through one of the other previous modalities (scRNA-seq-based differential gene expression, differentially expressed at the migrating wavefront of ENCDCs, conserved SOX10 binding motifs).

### *Sox10*^*Dom*^ aganglionosis modifier interval candidate gene prioritization pipeline from mouse datasets

Omics analyses used in prioritization were merged into a final table that was filtered to only candidate genes that were in three or more lines of omics evidence (Tables 6 and S24). A score of 0-3.5 was assigned to each candidate gene based on whether the modifier loci in which the candidate resides were 1) significant after multiple testing correction assigned 1 point, 2) LOD score of ≥3 assigned one point, and 3) overlapped with the modifier intervals from the F_1_-intercross study assigned 1.5 points (S25 Table; [20]).^7^ We do this to consider whether modifiers in which candidate genes reside could be false-positive associations.

### Filtering *Sox10*^*Dom*^ aganglionosis modifier interval candidate genes for those with intron or exon variants with predicted high impact

C3HeB/FeJ variants (VCF; mm39) for 30 candidate genes were used as input for the online Ensembl Variant Effect Predictor (VEP) for Mus musculus and were filtered for high predicted impact [62,89,95].

### Overlap of *Sox10*^*Dom*^ aganglionosis modifier interval candidate genes with human HSCR and stool frequency GWAS summary statistics

Summary statistics or analogous tables were downloaded for stool frequency and four different HSCR GWAS [10–13,63]. If all SNPs were available, false discovery rate (FDR) p-value adjustment was used, and SNPs were filtered based on FDR significance [10,12,63]. Each summary statistics table was verified to use SNP coordinates on hg19. SNPs were tested for LD with candidate genes via precalculated LD blocks [96]. These genes were then overlapped with their mouse orthologs from Table 7.

### Extraction of relevant phenotypes from Genebass-sourced *Sox10*^*Dom*^ aganglionosis modifier interval candidate gene-based PheWAS

Exome-based PheWAS from Genebass for variants in each candidate gene were filtered to the top 20 associated phenotypes per candidate [64,65]. These phenotypes were then filtered by common GI and nervous system terms as described in Supplementary Materials.

## Supporting information

S1 Fig QQ Plots of Non-Sex-specific and Sex-specific GEMMA genome-wide scans on the *Sox10*^*Dom*^ mice population. QQ plots visualizing GEMMA genome-wide scan results with inclusion of all chromosomes **(A)** and exclusion of chromosomes 5, 15, and X **(B)** for the total quantitative aganglionosis percentage phenotype. (**C**) QQ plot visualizing results from a GEMMA genome-wide scan in which a binary phenotype—either the individual mouse has or does not have aganglionosis measured—was used. QQ plots are also shown visualizing GEMMA genome-wide scan results of female- **(D)** and male-specific **(E)** runs for the total quantitative percentage aganglionosis phenotype. QQ plots visualizing GEMMA genome-wide scan results of female- **(F)** and male-specific **(G)** runs for the binary phenotype—either the individual mouse has or does not have aganglionosis measured.
(TIFF)

S2 Fig GEMMA genome-wide scans using only those *Sox10*^*Dom*^ mice that exhibit detectable aganglionosis. (**A**) Manhattan and QQ plots visualizing association analysis of quantitative aganglionic length, excluding unaffected *Sox10*^*Dom*^ mutation carriers. (**B**) Analysis as in A but only including male *Sox10*^*Dom*^ mice. (**C**) Analysis as in **A** but only including female *Sox10*^*Dom*^ mice.
(TIFF)

S3 Fig Stratification of allele-specific directions of effect by sex reveal sex effects. **A-E,I-N** The top 2 most significant SNPs per sex-specific genome-wide scans comparing the percent aganglionosis across individuals by genotype split by sex. **F** Chromosome 3’s top hit in the first GEMMA run shows a larger effect on males than females with rs13477043, and larger effects in females for genotype combinations for rs3667738, chromosome 8 **(G).** Similar differences in males and females are observed for rs13481145, the top hit for chromosome 11 **(H).** Each box plot shows distribution of percentage of aganglionosis and comparative statistics for each allele combination split by sex. Color of dots indicate the GEMMA runs with which each SNP is the peak associated SNP. See methods for the shorthand key for GEMMA association runs, which are the labels used here. Overall p: Kruskal-Wallis; internal p: Wilcoxon test. *, p < 0.05; **, p < 0.005; ***, p < 0.0005; ****, p < 0.00005; ns, not significant. A ‡ beside a SNP indicates significance of a SNP past multiple testing correction.
(TIFF)

S4 Fig Expression of genes in fetal gut near top associated SNP of nominally sex-biased modifier intervals across developmental time. Feature plots display expression via presence and intensity of purple split by developmental timepoint in the reprocessed [24] scRNA-seq dataset for candidate genes nearest to the top 2 associated SNPs or the most likely candidate gene based on known ENS developmental biology (*Ednrb*).
(TIFF)

S5 Fig CellChat identifies modifier interval candidates via estimated cell signaling with neural crest cells. (**A**) Dot plot split by time point visualizing probability of activity of significant signaling pathways (y-axis) by cell type (x-axis). Shape of the dot indicates which modifier interval each signaling gene is within. Signaling genes within modifier intervals are underlined. Significance is represented by the size of the dot. Cell types on the left (orange bracket) represent signaling into neural crest cells, while types on the right (blue bracket) represent signaling out of neural crest cells to those cell types. (**B**) Dot plot showing expression of genes within aganglionosis modifier intervals and their ligand-receptor partners. Cell types have been consolidated to those that are in Fig 5, and each cell type has split expression for developmental time in order, excluding 15.5dpc.
(TIFF)

S6 Fig Expression of *Sox10*^*Dom*^ aganglionosis modifier interval candidate genes from wavefront ENCDC bulk RNA-seq and near conserved SOX10 binding motifs. (**A**) UMAP of the neural crest cells from [24] highlighting neuronal and progenitor cells. UMAPs of the neural crest cells from [24] showing expression in purple of candidate genes that are upregulated in the migrating wavefront of ENCDCs (**B**) or are near or overlapping with conserved SOX10 binding motifs grouped by prior evidence (**C**), other data modalities supporting gene as a candidate) and number of binding motifs (**D**, two binding motifs; E, one binding motif).
(TIFF)

S7 Fig Whole gut 16.5dpc *Phox2b* H2B-CFP + snATAC-seq label transfer and derived putative cis-regulatory elements within modifier intervals. (**A**) Flow chart of analysis pipeline for differentially accessible chromatin contained within modifier intervals. (**B**) UMAP of scRNA-seq of 15.5dpc whole gut enteric nervous system cells annotated by supervised clustering used as a template for estimation of cell types in the snATAC-seq. (**C**) Estimation of cell types via Seurat and Signac’s LabelTransfer function used to annotate Fig 6B. Clusters from **B** are on the x-axis and clusters from Fig 6B are on the y-axis. (**D**) Annotations of differentially accessible (DA) peaks from all DA peaks (top) and those within modifier intervals split by cluster (bottom).
(TIFF)

S8 Fig Expression of progenitor, neuronal and candidate modifier genes across ENCDCs migration stages. Top column shows the Zhao et al. ENCDCs split by timepoint (columns) in chronological order and all cells (rightmost column) colored by cell state (progenitor, neuronal). Violin plots showing expression of marker genes (*Sox10*, *Phox2b*, *Ednrb, Elavl4*) relative to candidate modifier genes (*Dach1*, *Col1a1, Pdgfra, and Tbx2)* by cell state corresponding to each timepoint in the top row.
(TIFF)

S1 Table Tab-separated table with phenotypes of each mouse in the study listed by column, including length of intestine, length of hypoganglionosis and aganglionosis, percentages of hypoganglionosis and aganglionosis, sex of the mice, and mouse individual IDs. (TSV)

S2 Table Tab-separated genotype file in a PED-like format (PLINK) with the first columns being individual IDs for each mouse and columns per SNP genotype. (TSV)

S3 Table CSV file containing GEMMA scan results for all mice using sex as a covariate and the quantitative percentage aganglionosis phenotype. (CSV)

S4 Table CSV file containing GEMMA scan results for all mice using sex as a covariate and the quantitative percentage aganglionosis phenotype but excluding chromosomes 5, 15, and X. (CSV)

S5 Table CSV file containing GEMMA scan results for all mice using sex as a covariate and the binary aganglionosis phenotype (either no aganglionosis or yes aganglionosis). (CSV)

S6 Table CSV file containing GEMMA scan results for only mice that had aganglionosis using sex as a covariate and the quantitative percentage aganglionosis phenotype. (CSV)

S7 Table CSV file containing GEMMA scan results for only female mice that had aganglionosis using sex as a covariate and the quantitative percentage aganglionosis phenotype. (CSV)

S8 Table CSV file containing GEMMA scan results for only male mice that had aganglionosis using sex as a covariate and the quantitative percentage aganglionosis phenotype. (CSV)

S9 Table CSV file containing GEMMA scan results for male mice only using the quantitative percentage aganglionosis phenotype. (CSV)

S10 Table CSV file containing GEMMA scan results for female mice only using the quantitative percentage aganglionosis phenotype. (CSV)

S11 Table CSV file containing GEMMA scan results for male mice only using the binary aganglionosis phenotype (either no aganglionosis or yes aganglionosis). (CSV)

S12 Table CSV file containing GEMMA scan results for female mice only using the binary aganglionosis phenotype (either no aganglionosis or yes aganglionosis). (CSV)

S13 Table CSV file containing comprehensive information of modifier interval definitions derived from significant SNPs in S3–S5 and S9–S12 Tables. (CSV)

S14 Table CSV file that lists the genes within each modifier interval that are expressed above a normalized expression threshold of 1 in the Zhao et al. scRNA-seq dataset. (CSV)

S15 Table CSV file containing the significant (Bonferroni-correction p-value) differential gene expression results (Seurat’s FindAllMarkers function) for genes within the modifier intervals in the Zhao et al. scRNA-seq dataset. (CSV)

S16 Table Excel file containing significant results of the CellChat analysis in the Zhao et al. scRNA-seq dataset. (XLSX)

S17 Table TSV file containing migrating wavefront versus lagging enteric neural crest-derived cell significant differential gene expression results from Stavely et al. filtered for those within modifier intervals. (TSV)

S18 Table CSV file containing cross-species conserved SOX10 transcription factor binding motifs from Gopinath et al. that fall within modifier intervals, along with their closest genomic feature found through the ClosestFeature function from the R package Signac. (CSV)

S19 Table CSV file containing significant differential chromatin accessibility results (Seurat’s FindAllMarkers function) from 16.5dpc *Phox2b* H2B-CFP + ENS snATAC-seq. Cluster definitions were derived from transferring labels from 15.5dpc ENS scRNA-seq onto the snATAC-seq data.
(CSV)

S20 Table Results in S18 Table but filtered for those differentially accessible chromatin regions that lie within modifier intervals. (CSV)

S21 Table Overlap of the results in S17 and S19 Tables; those differentially accessible chromatin regions that overlap with conserved SOX10 transcription factor binding motifs. (CSV)

S22 Table CSV file containing significantly enriched transcription factor binding motifs derived from differentially accessible chromatin regions contained within modifier intervals (S19 Table) found via the R package Signac’s FindMotifs function. (CSV)

S23 Table CSV file containing the results from S21 Table filtered for those transcription factors that are expressed in the Zhao et al. “neural crest” subset of cells. (CSV)

S24 Table Excel file containing, for each omics candidate gene, the genetics score information. (XLSX)

S25 Table TSV file containing, for each omics candidate gene, the results from each omics prioritization method including: the p-value, method of quantitation/statistics, cell type (if applicable), age (if applicable), number of loci (if applicable), the modifier interval(s) in which a gene resides, the dataset from which the gene was prioritized, and the omics score (filtered for those omics scores greater than 3). (TSV)

S26 Table TSV containing Ensembl Variant Effect Predictor results for omics candidate genes whose C3HeB/FeJ variants were predicted to have high impact on the corresponding mRNA or protein. (TSV)

S27 Table CSV file containing Hirschsprung GWAS summary statistics from Tang et al. filtered by those within EUR (European ancestry group) LD of candidate genes. (CSV)

S28 Table CSV file containing Hirschsprung GWAS summary statistics from Tang et al. filtered by those within ASN (East Asian ancestry group) LD of candidate genes. (CSV)

S29 Table CSV file containing Hirschsprung GWAS summary statistics from Garcia-Barcelo et al. filtered by those within ASN (East Asian ancestry group) LD of candidate genes. (CSV)

S30 Table CSV file containing Stool Frequency GWAS summary statistics from Bonfiglio et al. filtered by those within EUR (European ancestry group) LD of candidate genes. (CSV)

S31 Table TSV file containing concatenated Genebass.org PheWAS results from this study’s list of candidate genes’ human orthologs. (TSV)
