# Supplementary figures and images for: Combinatorial multiomic analysis from a pedigree of Sox10Dom Hirschsprung mice identifies multiple high confidence candidate modifiers of Enteric Nervous System development
Source: PLoS Comput Biol. 2026 Jul 6;22(7):e1014424. doi: 10.1371/journal.pcbi.1014424 (PMC13372245; doi:10.1371/journal.pcbi.1014424)

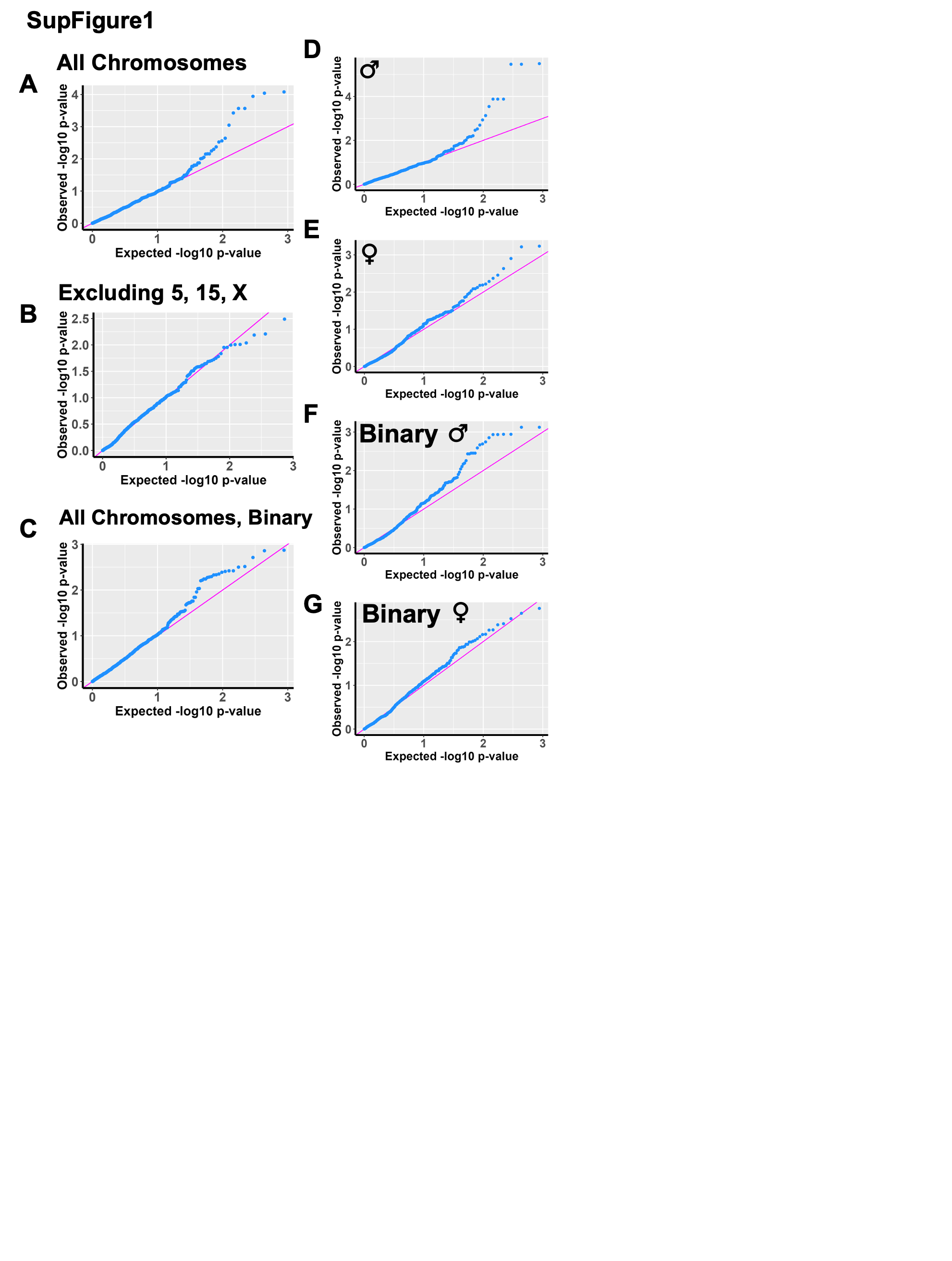

Supplement: S1 Fig — QQ plots visualizing GEMMA genome-wide scan results with inclusion of all chromosomes (A) and exclusion of chromosomes 5, 15, and X (B) for the total quantitative aganglionosis percentage phenotype. (C) QQ plot visualizing results from a GEMMA genome-wide scan in which a binary phenotype—either the individual mouse has or does not have aganglionosis measured—was used. QQ plots are also shown visualizing GEMMA genome-wide scan results of female- (D) and male-specific (E) runs for the total quantitative percentage aganglionosis phenotype. QQ plots visualizing GEMMA genome-wide scan results of female- (F) and male-specific (G) runs for the binary phenotype—either the individual mouse has or does not have aganglionosis measured. (TIFF) [file pcbi.1014424.s001.tiff]

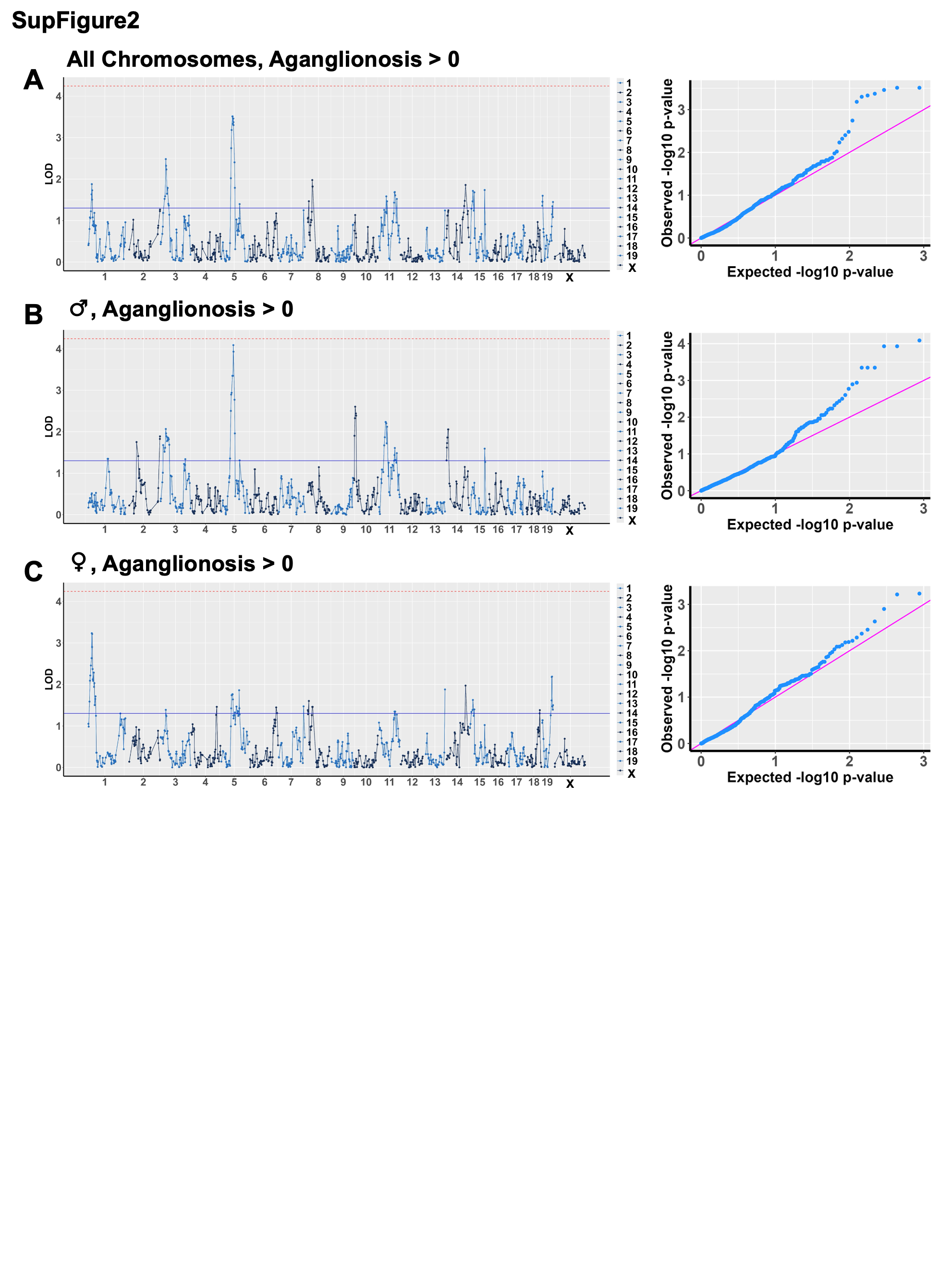

Supplement: S2 Fig — (A) Manhattan and QQ plots visualizing association analysis of quantitative aganglionic length, excluding unaffected Sox10Dom mutation carriers. (B) Analysis as in A but only including male Sox10Dom mice. (C) Analysis as in A but only including female Sox10Dom mice. (TIFF) [file pcbi.1014424.s002.tiff]

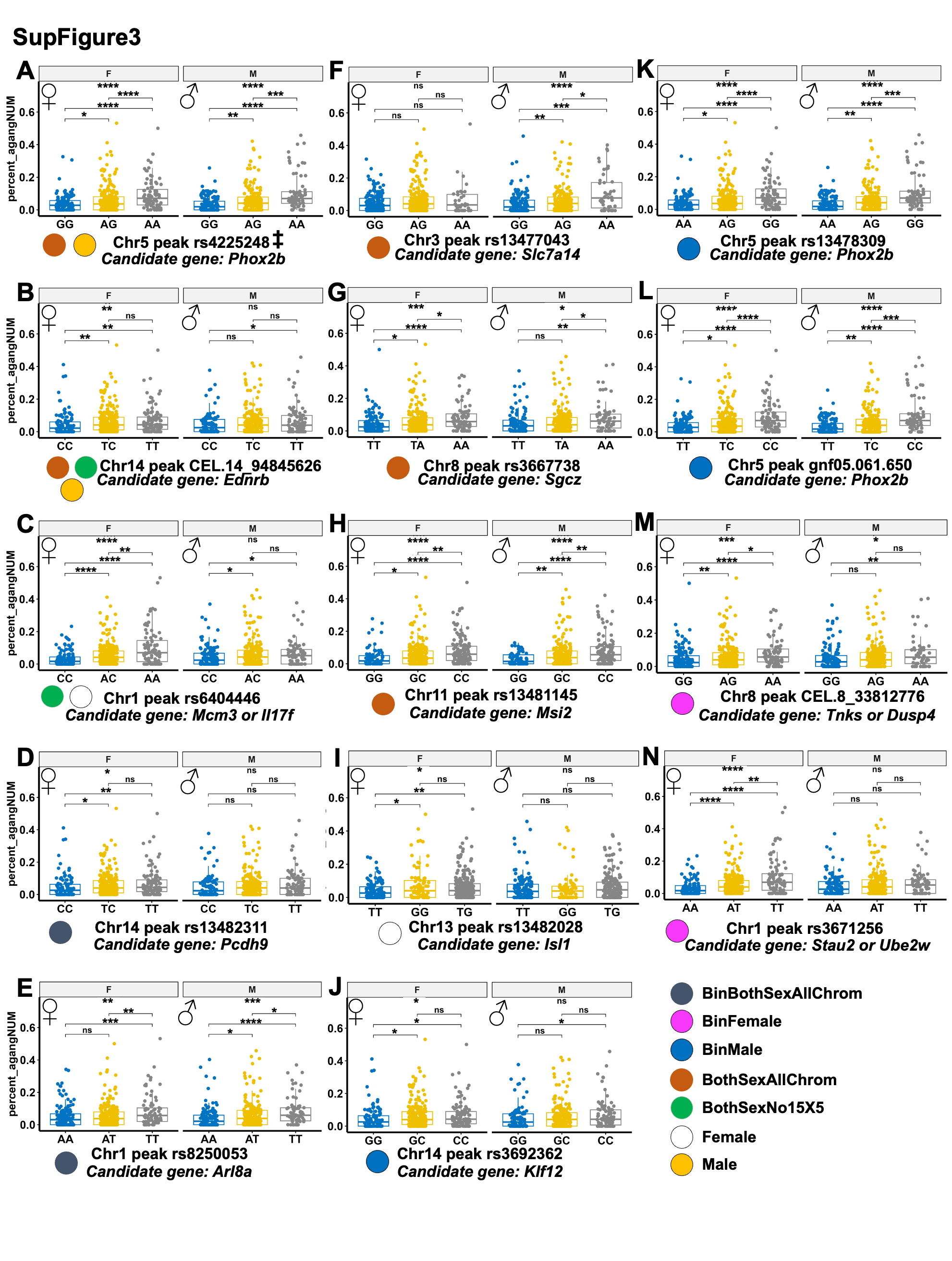

Supplement: S3 Fig — A-E,I-N The top 2 most significant SNPs per sex-specific genome-wide scans comparing the percent aganglionosis across individuals by genotype split by sex. F Chromosome 3’s top hit in the first GEMMA run shows a larger effect on males than females with rs13477043, and larger effects in females for genotype combinations for rs3667738, chromosome 8 (G). Similar differences in males and females are observed for rs13481145, the top hit for chromosome 11 (H). Each box plot shows distribution of percentage of aganglionosis and comparative statistics for each allele combination split by sex. Color of dots indicate the GEMMA runs with which each SNP is the peak associated SNP. See methods for the shorthand key for GEMMA association runs, which are the labels used here. Overall p: Kruskal-Wallis; internal p: Wilcoxon test. *, p < 0.05; **, p < 0.005; ***, p < 0.0005; ****, p < 0.00005; ns, not significant. A ‡ beside a SNP indicates significance of a SNP past multiple testing correction. (TIFF) [file pcbi.1014424.s003.tiff]

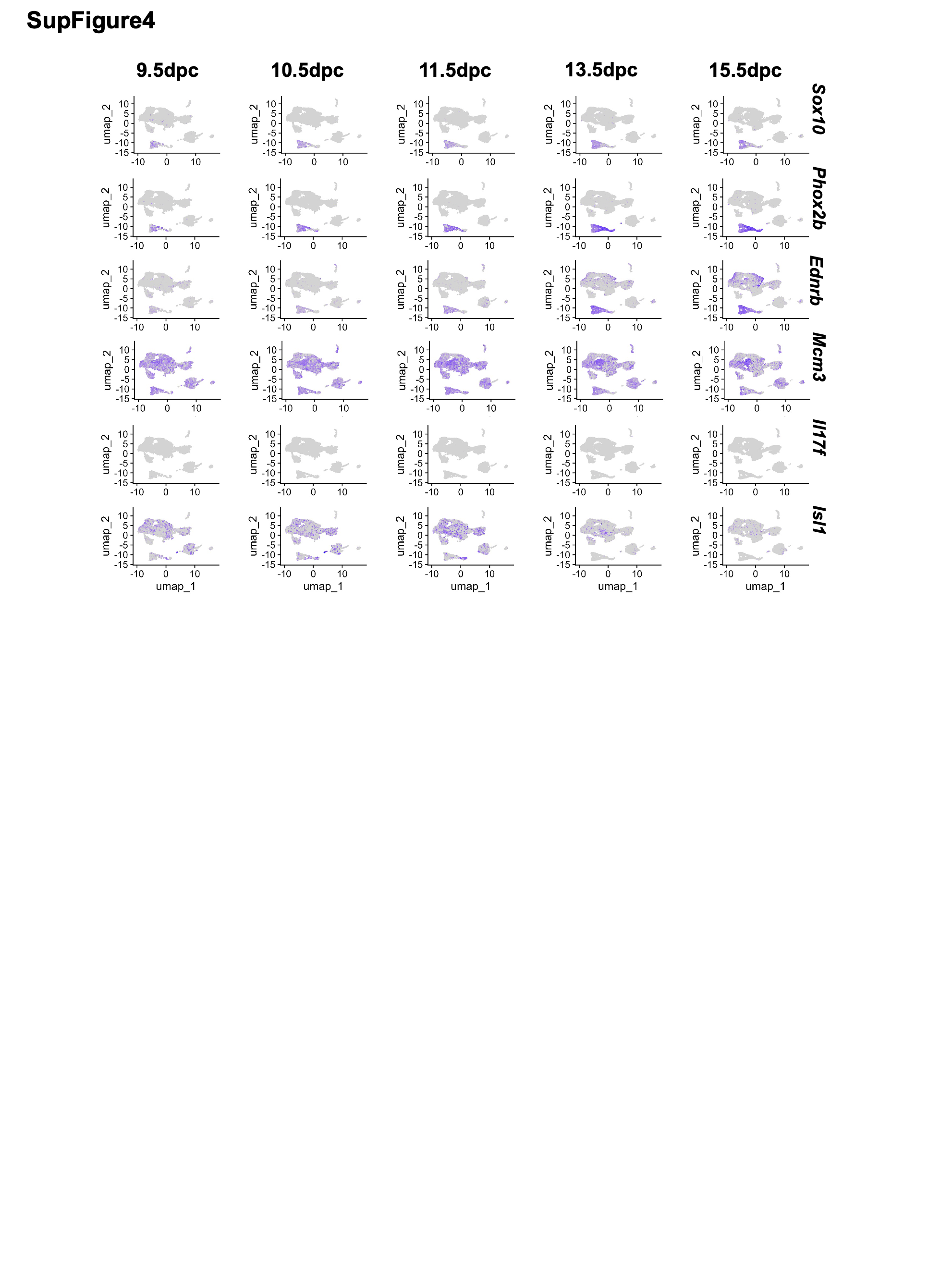

Supplement: S4 Fig — Feature plots display expression via presence and intensity of purple split by developmental timepoint in the reprocessed [24] scRNA-seq dataset for candidate genes nearest to the top 2 associated SNPs or the most likely candidate gene based on known ENS developmental biology (Ednrb). (TIFF) [file pcbi.1014424.s004.tiff]

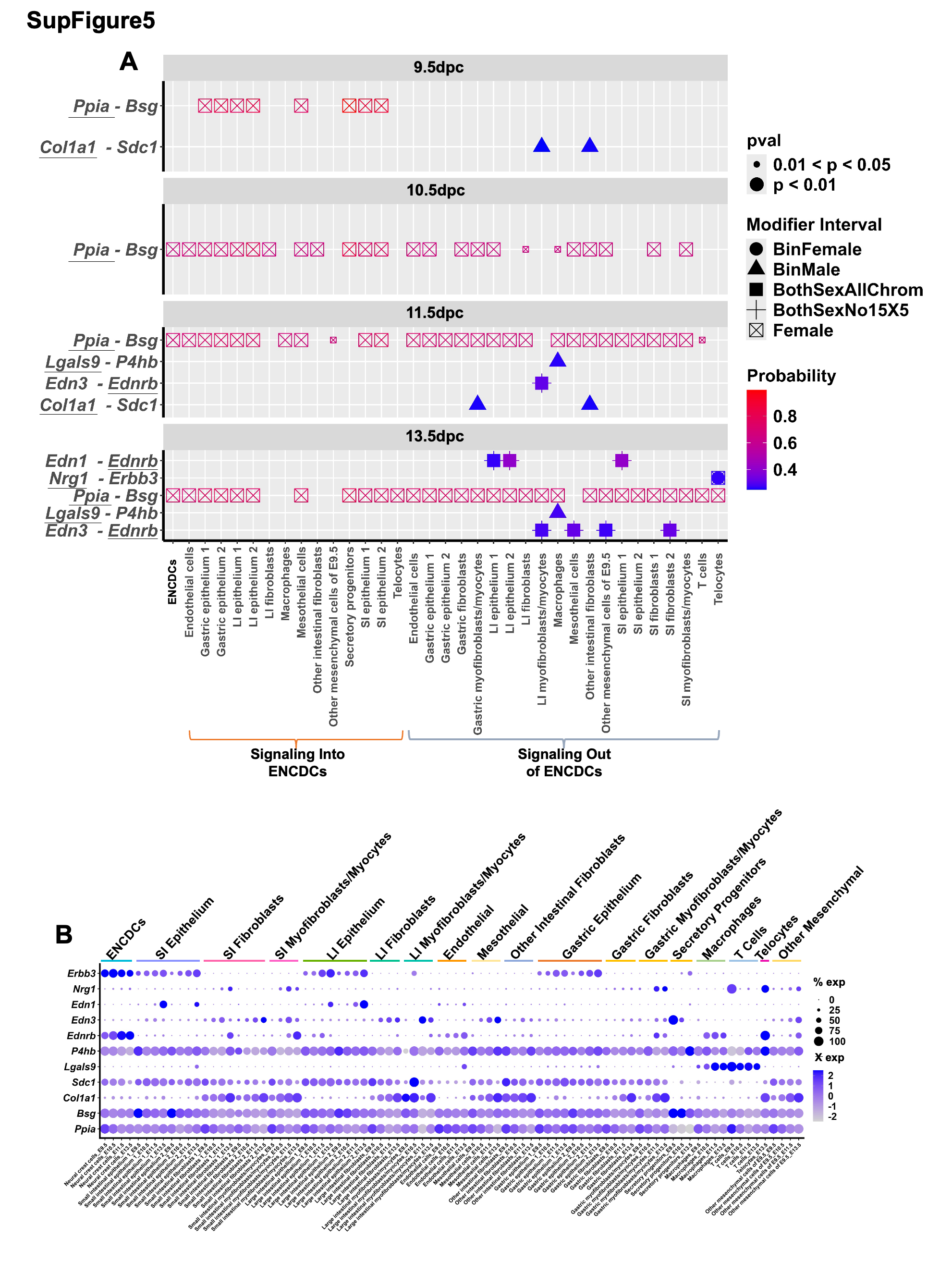

Supplement: S5 Fig — (A) Dot plot split by time point visualizing probability of activity of significant signaling pathways (y-axis) by cell type (x-axis). Shape of the dot indicates which modifier interval each signaling gene is within. Signaling genes within modifier intervals are underlined. Significance is represented by the size of the dot. Cell types on the left (orange bracket) represent signaling into neural crest cells, while types on the right (blue bracket) represent signaling out of neural crest cells to those cell types. (B) Dot plot showing expression of genes within aganglionosis modifier intervals and their ligand-receptor partners. Cell types have been consolidated to those that are in Fig 5, and each cell type has split expression for developmental time in order, excluding 15.5dpc. (TIFF) [file pcbi.1014424.s005.tiff]

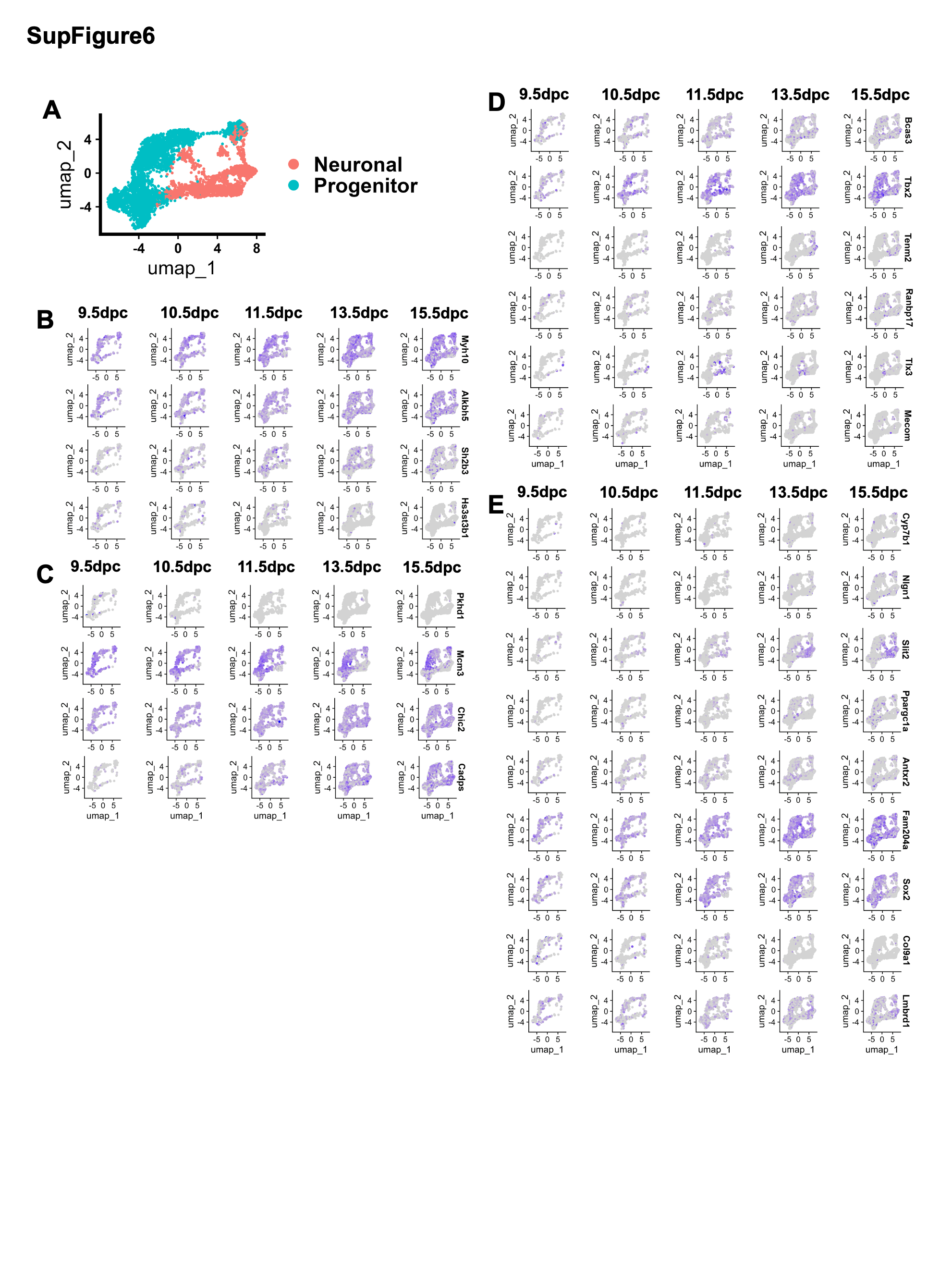

Supplement: S6 Fig — (A) UMAP of the neural crest cells from [24] highlighting neuronal and progenitor cells. UMAPs of the neural crest cells from [24] showing expression in purple of candidate genes that are upregulated in the migrating wavefront of ENCDCs (B) or are near or overlapping with conserved SOX10 binding motifs grouped by prior evidence (C), other data modalities supporting gene as a candidate) and number of binding motifs (D, two binding motifs; E, one binding motif). (TIFF) [file pcbi.1014424.s006.tiff]

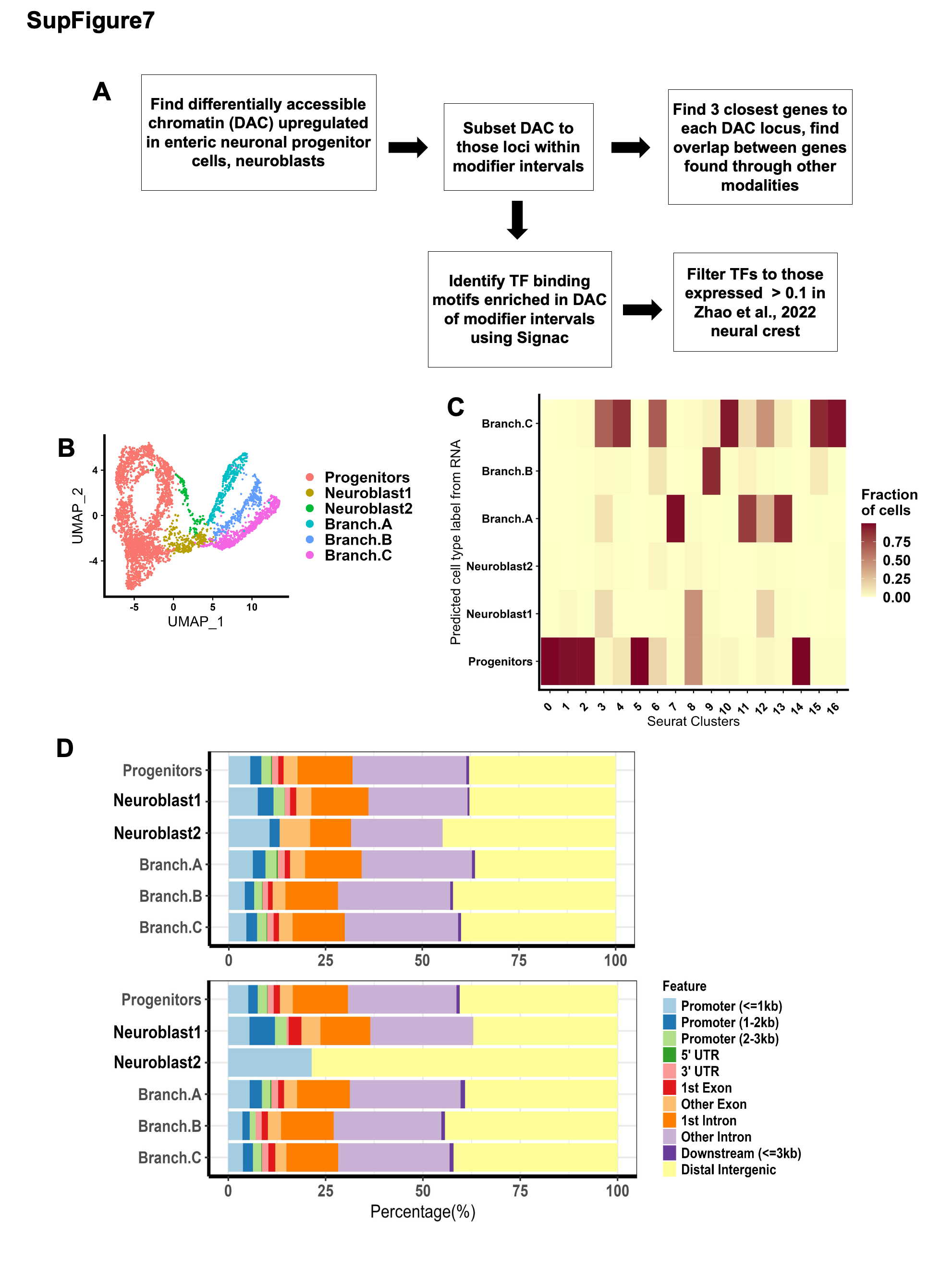

Supplement: S7 Fig — (A) Flow chart of analysis pipeline for differentially accessible chromatin contained within modifier intervals. (B) UMAP of scRNA-seq of 15.5dpc whole gut enteric nervous system cells annotated by supervised clustering used as a template for estimation of cell types in the snATAC-seq. (C) Estimation of cell types via Seurat and Signac’s LabelTransfer function used to annotate Fig 6B. Clusters from B are on the x-axis and clusters from Fig 6B are on the y-axis. (D) Annotations of differentially accessible (DA) peaks from all DA peaks (top) and those within modifier intervals split by cluster (bottom). (TIFF) [file pcbi.1014424.s007.tiff]

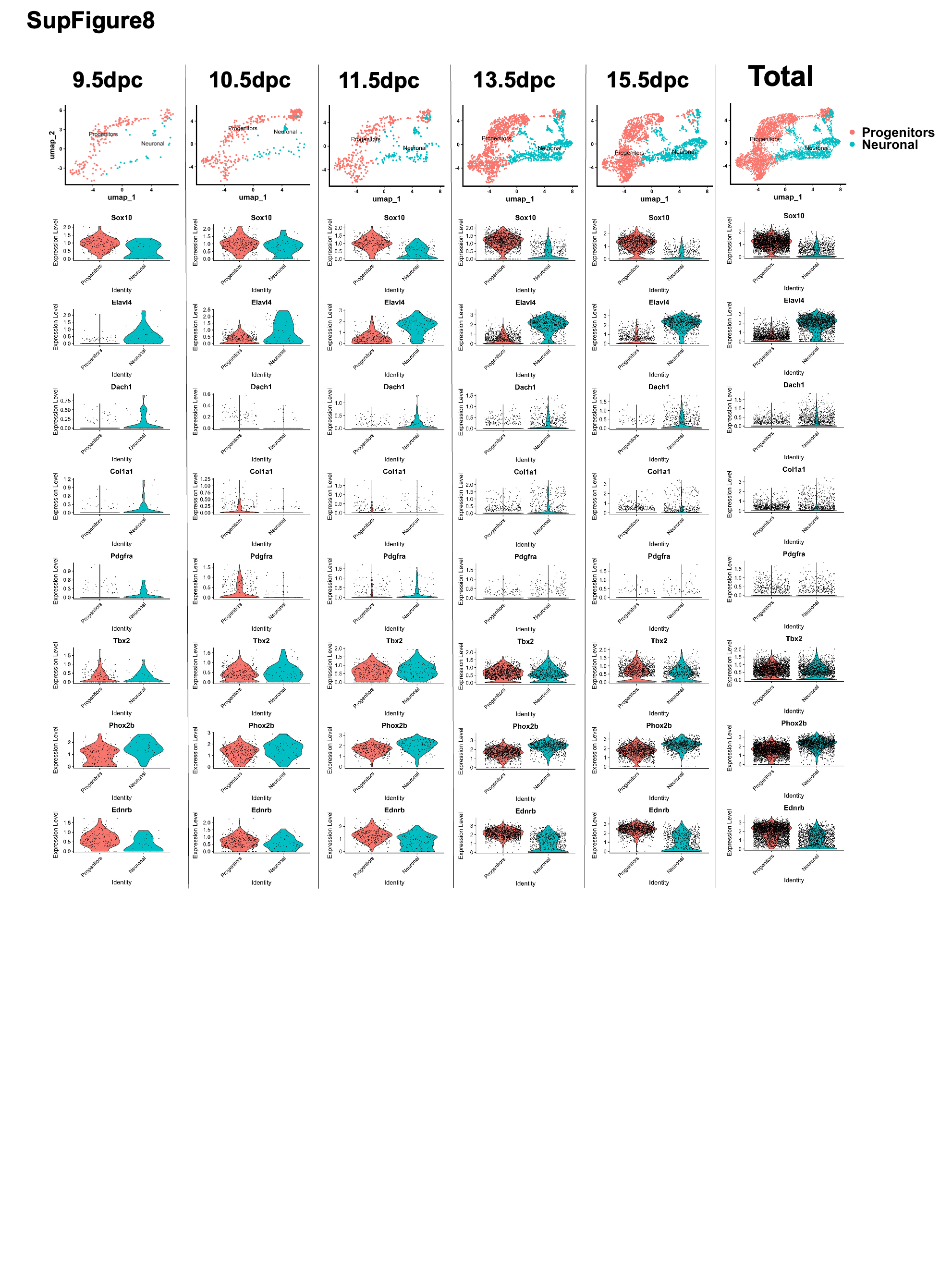

Supplement: S8 Fig — Top column shows the Zhao et al. ENCDCs split by timepoint (columns) in chronological order and all cells (rightmost column) colored by cell state (progenitor, neuronal). Violin plots showing expression of marker genes (Sox10, Phox2b, Ednrb, Elavl4) relative to candidate modifier genes (Dach1, Col1a1, Pdgfra, and Tbx2) by cell state corresponding to each timepoint in the top row. (TIFF) [file pcbi.1014424.s008.tiff]
