# Supplementary material for: Combinatorial multiomic analysis from a pedigree of Sox10Dom Hirschsprung mice identifies multiple high confidence candidate modifiers of Enteric Nervous System development
Source: PLoS Comput Biol. 2026 Jul 6;22(7):e1014424. doi: 10.1371/journal.pcbi.1014424 (PMC13372245; doi:10.1371/journal.pcbi.1014424)
Supplement: Attachment — Submitted filename: 2026-04-06_Response to Review PCOMPBIOL-D-25-02281.pdf [file pcbi.1014424.s042.pdf]

Response to Review Pedigree Paper PCOMPBIOL-D-25-02281

We greatly value the time and attention the reviewers have given to our submission and we are delighted that our article was found to be "rigorous", "conceptually clear", and an "important contribution to the HSCR field" based on " data driven strategy for discovery" that yields a "useful roadmap for future efforts aimed at delineating polygenic contributions to HSCR severity". We provide our responses below to clarify and expand upon the article so that prior concerns are addressed. We have added several additional figure panels, amended the text extensively, and responded point by point to reviewer comments. We believe these revisions improve the article and increase its value to readers of PLOS Computational Biology. Our responses to reviewer comments are below in blue text.

Reviewer 1 Points for Response

This manuscript takes advantage of an unusually large ten generation Sox10Dom pedigree, encompassing 830 heterozygous mutants with quantitative aganglionosis measurements and genome wide SNP genotyping, and uses this resource to refine modifier loci that influence Hirschsprung disease severity (segment length). The authors integrate these genetic signals with multiple publicly available and ENS relevant single cell, bulk RNA seq, snATAC seq and TF motif datasets to prioritize biologically plausible modifiers, with particularly strong convergent support for Phox2b, Ednrb, Nrg1 and Dach1. The conceptual framework is clear, the analytical workflow is rigorous and transparent, and the main conclusions are well supported by the data.

This is an important contribution to the HSCR field because two long standing questions remain unresolved: the genetic basis of segment length variation and the genetic underpinnings of the pronounced sex difference in disease prevalence. Despite decades of work, neither has been fully dissected. This study directly tackles one of these gaps, namely the genetic architecture of segment length defects, by combining classical mouse genetics with multiomic integration to pinpoint candidate modifiers of aganglionosis severity. The approach, and the prioritization framework it introduces, represent a step toward resolving why the extent of aganglionosis varies so widely even among individuals or animals sharing the same primary pathogenic allele. Conceptually, the work moves the field beyond broad, low resolution modifier loci and anecdotal candidate genes to a systematic and data driven strategy for modifier discovery. The integrative analysis that connects genetic loci to genes expressed in migrating ENCDCs, to chromatin accessibility in ENS progenitors and neuroblasts, and to human motility traits greatly strengthens the biological plausibility of the prioritized genes. Overall, the study provides a useful roadmap for future efforts aimed at delineating the polygenic contributions to HSCR severity.

Below are some suggested clarifications and analytical refinements to further strengthen the manuscript and maximize its impact.

We are delighted that the reviewer valued our submission and recognized the article's importance for the HSCR field. We are grateful for the thoughtful suggestions from the reviewer that demonstrated strong expertise in quantitative traits mapping to improve clarity of the analysis and results for the readers. We have made modifications to the text to clarify our analysis and improve readability. We have modified figures as requested with insertion of new figure panels and we have added two new figures. We have moved methods detail into the main text versus supplementary materials as recommended to

clarify our analysis and make these details most readily available to readers. We describe these modification in our point-by-point response below.

1. The authors correctly note that the aganglionosis proportion is heavily zero inflated. They deal with this by 1) using GEMMA on the quantitative proportion with zeros included, and 2) converting to a pseudo binary affected vs unaffected phenotype.

A. Consider including a brief comparison of models that treat the phenotype as

a. a rank based transformed quantitative trait

b. an ordered categorical trait (for example none, short, intermediate, long),

c. a true zero inflated or hurdle model if available in a mixed model framework for mouse pedigrees.

We appreciate the reviewer's interest in this aspect and note in our response the challenges associated with the distinct analysis modes. To address this concern, we have made the following modifications to the manuscript:

First - In the results section titled "*Genome-wide SNP analysis identifies Sox10^{Dom} aganglionosis modifiers*" we have added text that mentions how we assessed the possibility of using GEMMA with only mice that exhibited observable aganglionosis. This added section located on lines 233-237 reads: " Third, to assess SNP effects on only the variance in aganglionosis without effects of those with no detectable deficits, we performed GEMMA analysis using only mice with measurable aganglionosis (**Supp. Fig 2, Supp. Tables 6-8**). This approach produced an approximately 30 percent decrease of animal numbers available for the analysis, and therefore we do not consider these modifiers further."

Second – In the Discussion we have added a paragraph that describes the challenges of dealing with zero-inflated phenotypes on lines 692-711 and mentions possible alternatives for dealing with this kind of data as suggested by the reviewer.

Briefly this text includes:

a. use of a "rank-based transformed quantitative trait" given the zero-inflatedness of the data would break the assumption for that transformation because it would collapse all the zeros (about 30% of mice have zeros for aganglionosis) into a single rank or small rank set, which would ignore the structure of the trait distribution and could lead to spurious associations.

b. use of categorical methods as mentioned also collapses the data into categories, which produces loss of biological variation similar to the rank-based transformation and could lead to spurious associations.

c. We thoroughly investigated zero-inflated models and consulted with QTL expert Dr. Karl Broman. Dr. Broman recommended we use a two-part model in which zeros are treated separately from the rest of the data. In the initial manuscript, we utilized the "binary" phenotype method but left out the continuous-only analysis due to loss of ~30% of the sample size and therefore decreased power to detect signal. This loss of power was especially the case for the sex-specific GEMMA runs. These sex-specific GEMMA runs did, however, replicate the main hits found in the other zero-included quantitative trait-based GEMMA runs. For true zero-inflated models, we could not find an existing tool specifically for mouse that accounts for the inherent relatedness of the inbred strains. Additionally, models such as the zero-inflated negative binomial (ZINB, Moghimbeigi, 2015; https://doi.org/10.1016/j.jtbi.2015.02.016) are better for count-like data where our data are percentage-based (0-1). For ZINB, it would also be difficult to incorporate the

kinship matrix calculated by GEMMA into this model. We elected to apply a trusted method for GWAS on inbred model organisms despite the assumption being inconsistent with the expectations for GEMMA (residuals are non-normal), so as long as we 1) compensated with treating the trait as a binary phenotype in addition to quantitative and 2) replicated significant/marginal significant associations from the prior Owens et al., 2005 study.

B. At minimum, provide residual diagnostics or some statement that the GEMMA assumptions are reasonably met for the proportion phenotype, and that the key loci (for example the Phox2b region on chr5 and the Dach1 region on chr14) are consistently detected across plausible transformations.
Even if the authors decide not to add new models, a short section in the Methods or Results that documents sensitivity analyses would strengthen confidence in the mapping.

We appreciate this comment and emphasize that for GEMMA runs on the quantitative trait with zeros included, the residuals are non-normal and ,therefore, violate the assumption of residual normality for GEMMA. However, other methods that account for the assumption violations do not account for the relatedness between individuals in the extended *Sox10^{Dom}* mouse pedigree. As far as we are aware the tools do not exist to both account for relatedness and the non-normal nature of the residuals. The two-part model is the most biologically accurate approach available at this time. In an effort to address the binary-converted trait-based GWAS in the manuscript we have point to supplementary information (Supp Figure 2, Supp Tables 6-8_). We further described this aspect of the analysis in the Supplementary methods on lines 54-58 in the section titled "*Modifier intervals for downstream candidate gene analysis*" that states: "We performed GEMMA analyses using only mice that had aganglionosis without including those that did not have measurable aganglionosis.[5,6] We performed this analysis in order to have a two-part model (presence of aganglionosis, variance of aganglionosis within those *Sox10^{Dom}* mice with the phenotype), as, to our knowledge, no GWAS method exists that accounts for both inbred relatedness of our mice and the zero-inflated, non-normal phenotype distribution. We consider modifiers from the binary-transformed phenotype. However, our analysis using only *Sox10^{Dom}* mice with aganglionosis had lower power than the other analyses due to smaller sample size: we lose ~30% of mice as compared to the other GEMMA analyses. The modifiers detected via this method that were not detected via other methods were of marginal significance. Due to these reasons, we chose not to consider these modifier intervals in the main results (**Supp. Fig 2, Supp. Tables 6-8**)."

2. The manuscript relies heavily on loci that are only nominally significant (p Wald less than 0.05) after multiple testing, and then brings in biological replication and multiomic evidence to prioritize these. This is conceptually reasonable for a highly pleiotropic trait with modest sample size, but could be framed more clearly:

A - I suggest more explicitly separating
o genome wide significant loci (for example the Phox2b locus in males),
o FDR significant loci, and
o exploratory loci that are only nominally significant but supported by replication or omics integration.

We appreciate and value this request for clarity. We are concerned that structuring the presentation in this way will make the analysis approach and subsequent text appear very fragmented. Reviewer 2 has requested that we work to make the overall text flow better so we need to balance that request as well. We incorporated text edits to make it very clear that a single locus is genome wide significant after multiple test correction (Chromosome 5) with annotations to tables including legends for Tables 1, 2, and 6. The newly added Figure 4 also visually illustrates this aspect complemented by Supp Table 13 where the significance level of each modifier interval is provided. In addition, we have added text to the Discussion on lines _692-711_ that clarifies this aspect and emphasizes that majority of regions detected, while not meeting genome wide significance, replicate intervals detected in the Owens et al study and are supported by the omics integration .

B. In Table 1 and the main text, it would help to mark which intervals are formally significant after correction in at least one analysis, and which are purely exploratory.

We appreciate the suggestion to clarify this aspect. Only the Chromosome5 association is significant after multiple test correction. We have modified Tables 1, 2, 5, and 6 to note which were significant after multiple testing correction with additional text added to the Table legends as well.

C. The current modifier interval definition uses all contiguous SNPs with nominal p less than 0.05 plus or minus 0.5 Mb. Given the relatively sparse marker panel, this may generate quite wide intervals. The authors might provide summary statistics of interval sizes and, if possible, show that the main conclusions do not change with a slightly more stringent SNP inclusion threshold. Clarifying this hierarchy will make the story more convincing and avoid overinterpretation of weak statistical signals.

We value the reviewer's insights on this aspect. We emphasize that the intervals are already quite large (in some cases 10Mb) due to the SNP panel density even before adding the 0.5Mb on either side. Our reasoning for using 0.5Mb is elaborated in the Methods where we have expanded our rationale for this flanking interval size in the text to state: "The use of +/-0.5Mb interval flanking SNPs was chosen to account for associations that may not contain the causal SNP(s), gene(s), or locus (loci) and is consistent with many long-range regulatory elements that are within a 1Mb interval of regulated genes.". Due to sparse markers and some associations having a >1Mb distance from the affected gene, we used 0.5Mb flanking regions as a buffer to account for small intervals while not increasing the already large intervals even further. This a conservative attempt to add flank while also considering potential biology of variant functional distance.

As the reviewer requested, we did evaluate the outcome when the flanks were decreased to 0.25Mb. This approach decreases from 6216 genes for the 0.5 flanks to 5719 genes for the 0.25 flanks, which is 497 fewer genes. Within the 0.5Mb flank, there are 502 genes that are expressed, while in the 0.25Mb flank there are 466 genes, leaving a 36-gene differenced (Dbi, Nav1, Cdc73, Glrx2, Uchl5, Cd34, Nrd1, Cited4, Utp11, Psmd8, Yif1b, 2200002D01Rik, Spint2, Ppp1r14a, Oat, Gck, Mrpl22, Prkar1a, Trim27, Zkscan3, Thoc3, 4833439L19Rik, Nop16, Higd2a, Faf2, Rangap1, Phf5a, Aco2, Slc38a1, Slc38a2, Fam89b, Znrd2, Malat1, Neat1, Xpnpep1, and Il1rl1). None of these genes appear in the final candidate list based on the other omics analysis we utilized, so this exercise shows that the final results do not change if we reduce the interval flanks.

To address this concern in the text and provide further clarity, we refer the reader to Supplementary Table 13 which includes all intervals considered in this manuscript. We have added the distances of each interval to this table and include when an interval is nominally significant, LOD>3, FDR or Bonferroni significant.

3. Dach1 is a very appealing candidate, and the authors marshal several lines of evidence:

- **Upregulation at the ENCDC wavefront,**
- **Expression in ENCDCs that increases over developmental time,**
- **Overlap between conserved SOX10 motifs and Dach1 intronic regions,**
- **snATAC peaks in ENS progenitors overlapping these motifs, and**
- **Proximity to human stool frequency loci and GI surgery phenotypes.**

Given that there is no direct functional validation yet, I would suggest:

- **Slightly softening the title and abstract language. For example, instead of "implicates Dach1 as a modifier" one could say "prioritizes Dach1 as a high confidence candidate modifier of ENS development."**

We have revised the title to read: *Combinatorial multiomic analysis from a pedigree of Sox10^{Dom} Hirschsprung mice identifies multiple high confidence candidate modifiers of Enteric Nervous System development*

The abstract has been revised to read: " Among the novel genes, *Dach1* ranked as a top priority candidate gene for modifying migration of ENCDCs and thus influencing aganglionosis severity."

- **Adding one or two relatively low effort analyses that leverage existing data, such as:**
  - **o Showing the specific alleles at the Dach1 containing SNPs in the pedigree and the effect size on aganglionosis proportion, perhaps stratified by sex.**

While appealing, this strategy would only be possible with much greater genotyping density. In the SNP panel utilized, the closest SNPs to *Dach1* that were genotyped in the pedigree flank the gene at chr14:97201128 (rs13482327) and chr14:99776069 (rs3692362), where *Dach1* is at chr14:97786853-98169543. We are unable to implement a strategy looking at alleles of Dach1 given the SNP density and the data we have on hand. Therefore, we relied upon complementary omics to prioritize relevant genes such as *Dach1*.

o Examining co-expression of Dach1 with known ENS transcription factors (for example Sox10, Phox2b, Ret, Ednrb) in the Zhao scRNA seq dataset, for instance via a correlation heatmap across ENCDC subclusters.

We agree that co-expression of *Dach1* with *Sox10* is an important aspect and we have utilized screening for candidate gene expression concurrently in the ENS as a means to prioritize potential modifier genes. We offer expression feature plots for *Dach1*, with *Sox10, Phox2a, and Hoxb5* in Figure 5, which are relevant transcription factors for ENS development. These plots show that *Dach1* is expressed in progenitor populations that are concurrently expressing high levels of Sox10 transcription. However, we are aware that ***SOX10 protein persists in early developing neurons*** (Avila et al., 2025 DOI: 10.1016/j.jcmgh.2025.101590) therefore strictly relying on RNA levels in the same cells to prioritize candidate modifiers does not take into consideration the possibility that protein interactions may be affecting the severity of aganglionosis as ENS

progenitors undergo differentiation. To address this request, we provide a heatmap in our response that shows the overlap with *Sox10* since that is the gene mutation for which we are working to map modifier loci. As expected, since our criteria for candidates was that genes be expressed in the ENS and surrounding gut cells there are some cells that exhibit co-expression. Analysis of co-expression for other factors and receptors like Ednrb likely will confuse the reader and distract from the main focus of the article on combinatorial analysis to prioritize biologically relevant candidate modifier genes of *Sox10*^{Dom} during ENS development.

Response Figure 1 Inset Next Page: Overlap of Expression for *Sox10* and *Dach1*. Left set of panels are co-expression FeaturePlots displaying the sparse overlap between *Sox10* and *Dach1* in only ENCDCs (enteric nervous system; left) and in all gut cells (right). Each row is a timepoint indicating which cells from what timepoint are subset (9.5dpc-15.5dpc) as well as the final row being cells from all timepoints.

ENCDC Co-expression

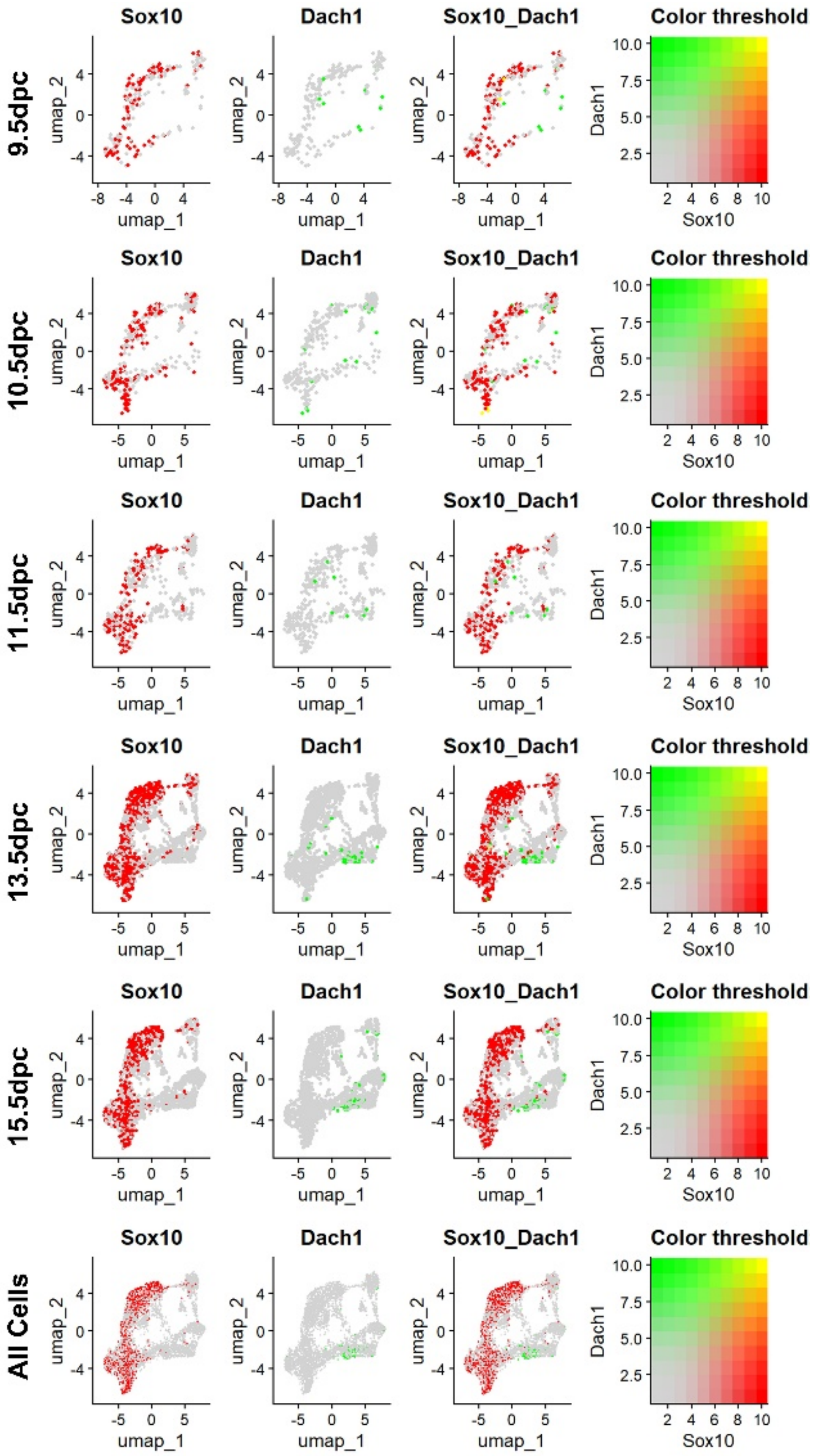

All gut cells Co-expression

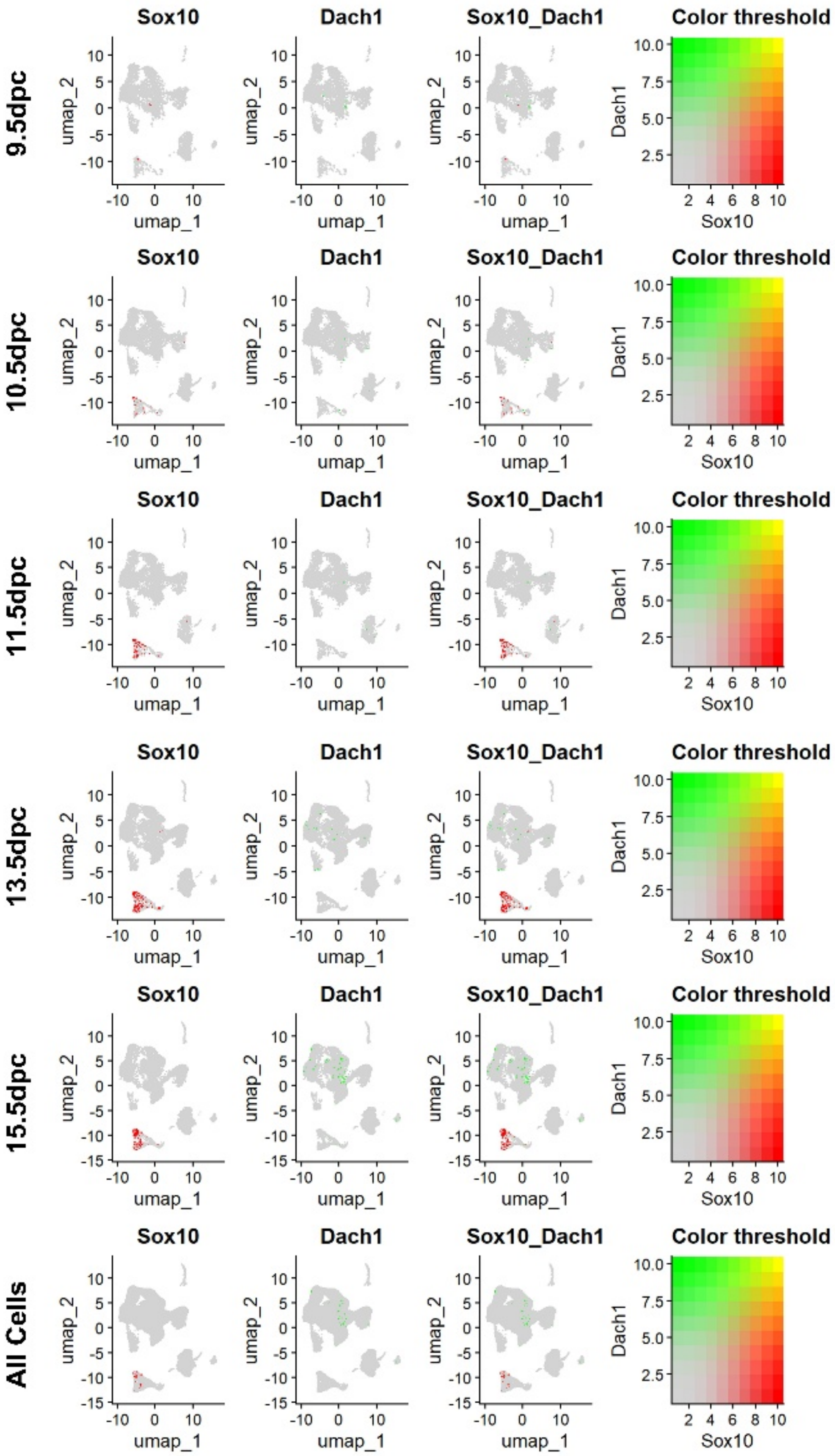

4. The dual scoring system is a key contribution of the paper but is currently somewhat buried in the text and supplementary tables.

- **I recommend adding a compact main figure panel that shows, for the top approximately 15 genes, both the modifier interval score and the omics evidence score side by side, perhaps as a bar chart or heatmap.**
- **In the Methods, please spell out exactly how points were assigned, including whether replicated loci and sex specific analyses were weighted differently, and how genes that fell in multiple intervals were handled.**

This will make it easier for readers to appreciate why Dach1, Phox2b, Ednrb, Nrg1 and others rise to the top.

We appreciate the reviewer's encouragement to bring this important scoring metric to the forefront. We have restructured the text and figures to emphasize the dual scoring system:

We have added a new figure panel 7B that illustrates combined scored metrics for the 19 candidate genes that exhibited both genetic and omics evidence. We refer the reviewer to the Results section titled "Prioritization of top aganglionosis modifier candidate genes across data modalities" where we elaborate on how points are assigned based on multiple test correction, LOD score of >3, and replication from the prior F1 intercross (lines 543-548) that states "For our modifier interval prioritization score, we assigned 0 to 3.5 points to each gene within modifier intervals based on the following criteria to minimize false positives: 1) peak SNPs of modifier intervals significant after multiple testing correction were assigned one point, 2) peak SNPs of modifier intervals with LOD score of ≥3 were assigned one point, and 3) intervals replicated from the prior F_1-intercross study were assigned 1.5 points (**Table 6**, **Supp. Table 24;)**". Sex specific loci were not weighted differently.

The methods section further describes the scoring process (lines 952-956). These scores are included in Table 6 where modifier scores and specific elements of omics evidence are summarized for the top 19 candidate genes.

5. The links to human stool frequency GWAS and PheWAS are intriguing, but currently described in a fairly qualitative way. Within reason, the authors could strengthen this section by:

- **Stating clearly which human loci are within the same LD blocks as the mouse orthologs and which are more distant, perhaps in a concise table.**

We have moved the information on Human GWAS LD blocks and location of GWAS associated SNPs into a separate summary table (Table 8) titled "Human genome-wide significant SNPs that are in LD with candidate gene orthologs identified in *Sox10^Dom^* modifier analyses". All genes listed in this table are in LD blocks that were significantly associated with phenotype in the human studies. This table offers a range of distances for the associated SNPs. The associated text of Results states "Two separate HSCR GWAS summary statistics had significant SNPs that were in LD with genes identified in this study, including *Phox2b*, *Col1a1*, *Rbpj*, *Nrg1*, *Ednrb*, *Hoxb2/5/13*, and *Uchl1* (**Table 8, Supp. Tables 27,28,29**; Garcia-Barcelo et al. 2009; Tang et al. 2016). The GWAS of stool frequency had significant SNPs that were in LD for *Col1a1*, *Rbpj*, *Nrg1*, and *Elf1* (**Supp. Table 30**; Bonfiglio et al. 2021)." These changes clarify and strengthen this section.

- **Clarifying whether effect directions for variants near candidate genes are consistent with increased or decreased motility.**

While assessing direction of effects for SNPs near candidate genes on motility is an appealing proposition, the phenotype measured in *Sox10*^{Dom} pups was based on aganglionosis only and motility was not measured. Similarly, the human HSCR GWAS studies did not measure motility and only recorded a case-control phenotype for HSCR. The Stool Frequency GWAS (Bonfiglio et al.) is a proxy phenotype for motility where increased stool frequency could possibly reflect increased motility but motility is not the value reported by the Bonfiglio study. We are concerned that attributing a specific SNP near a human candidate gene with some level of functionality related to motility may be over-interpreting the data and require a leap of faith beyond simply relating the candidate modifiers of aganglionosis to human studies of gut phenotypes.

- **Describing the strategy for the text based search of ENS related phenotypes in Genebass, and acknowledging that this is exploratory.**

We have elaborated on the process details of the text-based search for ENS related phenotypes in Genebass in the last section of the Supplemental Information titled "Extraction of relevant phenotypes from Genebass-sourced *Sox10*^{Dom} aganglionosis modifier interval candidate gene-based PheWAS" on lines 977-983.

We have further modified the wording in the Results section where this is first mentioned to indicate that this is an exploratory approach. The revised phrasing states "we took an exploratory approach to probe the publicly available exome-based PheWAS sever Genebass".

Minor comments and suggestions

1. Methodological clarity

a. In the GEMMA methods, please state exactly which covariates were included (for example sex, batch, generation) and whether intestinal length was used as a covariate when modeling aganglionosis proportion.

We have expanded the methods section of the article and include all relevant parameters for GEMMA in the supplementary methods section. Only sex was used as a covariate. This is stated explicitly in the supplement on lines 21-23 as "A column of 1's and sex were used as covariates for the GEMMA analyses not split by sex, while analyses split by sex used only the column of 1's as described in the GEMMA manual." We did not use intestinal length as a covariate since during phenotyping we specifically measured gut length and length of aganglionosis then converted these values to a percentage of total gut length affected to account for any impact of nutrition status on animal size that might affect gut length.

b. For the snATAC seq analysis, consider giving a brief description of peak calling thresholds and how differential accessibility was defined (for example log fold change cutoffs, FDR thresholds).

We have moved this information from supplementary methods to the main methods in the body of the article stating "Differential chromatin accessibility analysis was performed using MACS2-derived peaks and Seurat's FindAllMarkers function for both unsupervised cluster and LabelTransfer clusters.[27] The resulting differential chromatin accessibility datasets were

filtered by Bonferroni-adjusted p-value < 0.05 and average log2 Fold Change > 1.5 and < -1.5."

2. Figure clarity

• In Manhattan plots, label the chromosomal positions of the key genes (Phox2b, Ednrb, Dach1) directly on the plots to guide readers.

We appreciate the reviewer's enthusiasm for the genes that we have identified through a combined analysis of multiple data modalities. However, we are concerned that overlaying the genes on the Manhanttan plots could lead to misinterpretation of those GEMMA data, which are strictly association with SNPs across the genome. We detect *Dach1* through a combination of genetics and complementary omics analysis. Adding gene labels to chromosomal positions could cause some naïve readers to think that we directly evaluated association with those genes, which was not the case.

• In the UMAPs that focus on ENCDCs, it would help to clearly label progenitor vs neuronal clusters and to add a schematic of gut colonization timing for readers less familiar with ENS development.

As suggested, we have added a schematic of gut colonization timing to Figure 5B. We previously had labels for UMAP clusters that indicated progenitor and neuronal populations in the Supplementary Figure 6 and we have adjusted Figure 6 to include this aspect for greater clarity.

3. There are a few duplicated words or minor typos (for example "extended pedigree pedigree" in the abstract) that should be corrected.

We thank the reviewer for their careful attention to detail in reading our submission and we have edited the abstract and manuscript text for correctness.

4. The author should add the reference PMID: 30970187 in the introduction, where they discuss "human studies identified variants or genes that influence the penetrance of HSCR" (Line 117, page 5). This is to date the largest genome screen on HSCR.

We have added the citation for PMID: 30970187 into the list of references that describe "human studies identified variants or genes that influence the penetrance of HSCR".

<u>Reviewer 2 Points for Response</u>

In this study Benthal et al set out to uncover the gene interactions that may underlie the variable severity of Hirschsprung's (HSCR) disease. HSCR is neurocrisptopathy whereby neural crest (NC) derived enteric neurons fail to populate the enteric nervous system causing severe gastrointestinal defects in patients. A number of genes are well established to play a role in HSCR, including SOX10 which is the focal gene of this study. However, to date no study has uncovered the combinational action of genes in HSCR and how these combinations may influence the severity of disease.

We are pleased that the reviewer appreciates the novelty of our analysis, which combined genomic association analysis, gene expression, and chromatin accessibility (completely novel snATAC-seq data for developing enteric neurons and progenitors/glia) in order to distill and prioritize genes that are the highest priority candidate modifiers within large genome intervals that are typical for quantitative traits like aganglionosis. This is the

first report that takes a computational approach to mine highly complex omics data for the identification of novel genes that likely regulate the extent of aganglionosis in HSCR mouse models.

For their study Benthal et al selected the Sox10dom mouse line, which is appropriate since this is a well-established Sox10 mutant line which displays HSCR (among other neural crest phenotypes). However, from the outset it is not clear how the authors quantified the severity of aganglionosis, there is mention of acetylcholinesterase staining, but this is not shown or elaborated on. Since this is crucial for the rest of the work presented, I have reviewed the manuscript assuming there has been some robust quantitative assessment of the severity of aganglionosis, if it so transpires that this is lacking, I'm afraid all subsequent analysis is unsubstantiable.

The initial methods section under the animal husbandry section stated "Whole-mount acetylcholinesterase enzyme histochemistry of all *Sox10*^{Dom} mutant offspring from the pedigree collected for this study was performed as described by Enomoto and colleagues with subsequent measurement of total gut length from pyloric sphincter to anus and aganglionosis based on acetylcholinesterase stain as previously reported (Enomoto et al. 1998; Cantrell et al. 2004)." We have now moved and greatly expanded this description into a stand-alone methods section titled "*Acetylcholinesterase histochemistry*" so that it is more apparent to readers. Further, we elaborate in the text of this section how the extent of aganglionosis was rigorously quantified so this aspect is very clear, and we have added a schematic to Figure 1 that illustrates this process. Our detection of the Chromosome 5 locus, that was previously reported as a modifier interval for aganglionosis, substantiates this approach.

The study frequently refers to data/results from other studies, particularly Owens et al 2005. Arguably this helps to place the data within the broader context of the field, and it is good practice to utilise existing data, however it seems in places this study is quite dependent on previous work to substantiate the new analysis. In places this makes it quite challenging to unravel what is novel and meaningful from the current work.

We regret that the reviewer found the analysis challenging to follow. The current studies do not rely on the prior Owens 2005 work and are an independent body of analysis. It is reassuring that the Chromosome 5 locus we detect in the genome wide analysis from the pedigree study recapitulates the prior significant interval detected in the Owens 2005 publication. In genetic studies replication is considered a strength. Moreover, we take the novel approach of combining genetics association outcomes (modifier intervals) with gene expression and chromatin accessibility to aid in discerning which among the many genes that reside in genetic modifier intervals are logical, high-priority candidates to be investigated further. We have added a new figure to illustrate this process (Figure 4B) to clarify the process for readers.

The manuscript includes a large number of tables (8), if some of the data in these tables could be presented in a more tangible way that would greatly enhance the readability of the paper and better demonstrate the key findings.

We regret this reviewer found the tables were not presented in an optimal manner. The tables are designed to emphasize key aspects of the study. We have removed Table 5 and we have consolidated the information from Tables 1 and 2 into a new figure (Figure 4A). tables.

The article is not cohesive; each results section presents some data but then this is largely unrelated to the next section. The authors don't focus enough on any particular finding they just jump to the next analysis, e.g. they explore some gene expression patterns in single-cell RNA-seq data but then use bulk RNA-seq data to look at other genes, both gene sets are not explored in both data sets, which is disappointing and frustrating and indicates potential weaknesses in the current analysis if the findings are not consistent across different analysis pipelines.

We understand the reviewer's concern that the results sections in the original draft may have seemed unrelated. We have reorganized the text and added additional sentences as transition information between each of the results sections to aid with the flow of logic in the article emphasizing that the overall goal of the study is to identify those genes that are the highest priority modifier candidate genes within the detected modifier intervals and we have added an additional overview figure (Figure 4B) to illustrate this process. We believe this restructuring and additional language makes the text more cohesive.

Regarding the concern about the differences in analysis between the single cell data and the bulk RNA-seq, we reiterate that the point of using an ensemble of diverse datasets is to identify what is consistent across the multiple methods. These two data types are examined separately because these are not comparable in terms of source tissue. The scRNA-seq is whole gut tissue without emphasis on the ENS, although **we do extract the ENS cells from the dataset later to look at the bulk RNA-seq differentially expressed genes that are upregulated at the migrating wavefront, which the reviewer may have missed**. Further, we do not expect that genes that are upregulated or exclusively expressed in a minority of the wavefront cells of the migratory ENS detected in the bulk RNA-seq data would also be expressed in all other lagging cells (scRNA-seq ENS).

Section Specific Comments

Genome-wide SNP analysis identifies Sox10Dom aganglionosis modifiersIn this section the authors use Genome-wide Efficient Mixed Model Analysis (GEMMA) to predict causative SNPs in Sox10Dom animals. They use 830 pups, which seems excessive, was there a need for such high numbers of animals?

Linkage studies and GWAS benefit from high sample size to have sufficient power. In comparison to some contemporary studies, 830 pups is a relatively small study.

The authors mention comparing SNPs in animals that died younger, i.e. had more severe phenotype to those that survived to sexual maturity and therefore possibly had protective alleles. This would be a really interesting comparison and arguably essential to the question of which genes are involved in more severe cases of HSCR, but no such comparative data is included.

While this approach would be an interesting comparison, it would require restructuring the analysis to look at survival to breeding age rather than aganglionosis as the primary defining trait for Hirschpsprung disease. Moreover, just relying on death as a datapoint is not a reliable measure of aganglionosis since sometimes pups die because of competition among littermates or poor mothering by the dam. Therefore, collecting remnants of pups that died, which are often partially cannibalized by cagemates, does not ensure those pups had extreme aganglionosis. We intentionally structured the data collection to quantify aganglionosis extent in animals at P7-P10 to ensure that any severely affected pups, which would not survive to breeding age, would be sampled. It is reasonable to presume that those

Sox10^{Dom} mice in our population with no measurable aganglionosis would survive to sexual maturity, and we do compare these mice versus the rest of our population in our study.

Did the authors look at any GWAS data from human HSCR patients, and did they observe any shared SNPs with their mouse model?

We are pleased that the reviewer is interested in the possibility of mouse human comparisons which we did include in our article. Comparisons of individual SNPs across species is not reliable, so we utilized a gene-based approach. At the end of our prioritization pipeline, we compared genes that were in linkage disequilibrium with significant SNPs from human HSCR GWAS to our prioritized candidate gene list. In addition, we emphasize that the study design for our GWAS was based on length of aganglionosis, while human HSCR GWAS have always been case (individuals with HSCR) versus control (unaffected individuals) comparisons. Therefore, we do not anticipate that comparing SNPs from our mouse GWAS, which evaluated extent of aganglionosis, with those from human HSCR GWAS, that examined whether or not an individual has HSCR, would be fruitful.

Did the authors ever cross female Sox10dom/+ with WT males and if so did they observe the same phenotype variability as when they crossed male Sox10dom/+ with WT females?

We did not utilize a breeding strategy that relied upon due *Sox10*$^{Dom/+}$ females because these dams are very unreliable mothers with resulting poor survival of all pups. Such an approach would have significantly lengthened the course of the study and would have introduced confounding effects from mothering efficiency. When we have used *Sox10*$^{Dom/+}$ mothers for general breeding purposes in our colony, we do observe variability in survival of resulting *Sox10*$^{Dom/+}$ pups, which is correlated/consistent with aganglionosis length.

Figure 1A is not very informative. The text 'Extent of aganglionosis was assessed by whole-mount acetylcholinesterase staining collected over multiple pedigree generations (Fig 1A; Supp. Table 1)' suggests the acetylcholinesterase staining is shown here but it is not. Given this staining is the gold standard technique to diagnose HSCR disease and from the outset the authors plan to unravel the combinatorial causes of variable aganglionosis in HSCR it must be shown here.

While the quantitation of percent gut length affected by aganglionosis was previously describe in detail (Cantrell et al., 2004), and we do reference this publication, we have addressed this concern by incorporating a new panel into Figure 1 (panel B) that illustrates how the aganglionsis extent is quantified that includes panels showing images of normally stained ENS ganglia and bowel regions that are aganglionic

Figure 1B, why was intestine length measured? This is not a phenotypic factor in HSCR disease, so this seems irrelevant. Furthermore, why were female and male intestine lengths compared? It is well established that this parameter is fairly consistent between sexes, a more relevant comparison would be intestine length in WT versus Sox10dom/+ animals. Although as my first point here, it would not be expected to see a difference in intestine length between WT and Sox10dom/+.

Total intestinal length was measured so that extent of aganglionosis could be evaluated as a percentage rather than a raw metric. This aspect is important because pup size can vary such that use of either raw intestinal length or raw measurements of aganglionosis length might lead to spurious GWAS results. We therefore utilized a percentage of aganglionosis of the length of the intestine. Addition of the new figure panel (Figure 1B) and Results text further clarifies this aspect.

WT mice are not utilized in this study other than for maintenance of the pedigree.

What data is presented in Figure 1D?

Figure 1 renumbered panel E plots the percentage of gut length that is aganglionoic separately for males and females as described in the figure legend. The percentage of intestinal aganglionosis was utilifized in our GWAS analysis to avoid effects from differences in pup size.

Table 1 is quite 'dry'. Could the authors include some more tangible information, eg what is the nearest gene the SNPs region?

Modifier genome intervals are quite large (some >10Mb) and thus include many genes. As a result, we do not include genes in this table as it would be extremely difficult to display. We refer the reviewer to Table 3 where the nearest genes to significantly associated SNPs are listed.

The GEMMA analysis is conducted on several variables, such as removing different chromosomes, male vs female etc. Whilst this broad analysis could be useful, no consensus seems to be made as to what predicted SNPs are most informative. On the Manhattan plots in Figure's 2 and 3, what is the difference between the blue and black traces? The discovery of a SNP ~48kb upstream from Phoxb2, a known player in ENS development and function is surely of significant interest and yet this is not further explored. It is also notable that this SNP and one near Ednrb were attenuated in females compared to males, are their gender specific differences in HSCR genotype in humans? This is an important point and should be elaborated on.

We now include an new figure panel 4A that illustrates the consistent detection of modifier intervals across the distinct GEMMA analyses we performed.

The blue and black traces correspond to signal from distinct chromosomes indicated by the chromosome number on the x-axis. It is common practice to distinguish chromosomes by distinct colors. We have added text to the figure legend to clarify this aspect that states "Alternating blue and black traces represent the plotted LOD values for distinct chromosomes with corresponding chromosome number indicated below each trace on the x-axis."

Because Phox2b is a known player in ENS development and was previously detected in the Owens et al study, it is considered an internal control for the analysis as we now clarify in the revised Results text stating "While detection of significant association near *Phox2b* serves as an internal control based on prior reporting of this region, we sought to identify other novel candidate modifier genes with the potential to influence aganglionosis extent." We elected to assess other modifier intervals to identify other novel genes as candidate modifiers of aganglionosis.

There are sex differences in the incidence (how frequently HSCR occurs) for this disease in humans with males more commonly affected than females. In the $\mathit{Sox10}^{\mathit{Dom}}$ pedigree analysis, males were not more frequently affected than females as shown by the similar numbers of pups collected (Results line 191) and the similar distributions observed for gut length affected by percent aganglionosis (Figure 1E).

Later in the paper the authors use different subsets of SNPs for further analysis, again muddying the waters as to what is really meaningful from their analysis.

The same set of SNPs was used for each GEMMA analysis. How the analysis was structured (e.g. all chromosomes, or leave out Chr5/15/X) resulted in different associations of SNPs based on the affect of genome regions on phenotype. We subsequently utilize all potential modifier interval signal and we prioritize genes within the broad range of signals

detected using either publicly available omics datasets or the novel ATAC-seq dataset that we generated.

Prioritization of candidate genes based on expression in the fetal mouse intestine
Following their GEMMA analysis, the authors use the UCSC table browser to predict genes associated with the SNP containing genomic intervals. It is not clear which intervals they annotated in this way, did they use a consensus set from all their GEMMA analyses or just those that met their aforementioned criteria 'We prioritized consideration of associated loci that A) were significant after multiple testing correction, B) had a logarithm of the odds (LOD) score of ≥3, or C) replicated observations of an independent F1-intercross study (Owens et al. 2005)' or did they include all 'nominally significant' intervals?

As the reviewer has surmised we utilized the UCSC browser to identify genes associated with all modifier intervals that met ANY of the listed criteria (A) were significant after multiple testing correction, B) had a logarithm of the odds (LOD) score of ≥3, or C) replicated observations of an independent F1-intercross study).

The modifier intervals are sets of SNPs that are all nominally significant or are significant after multiple testing correction that are adjacent to each other. We do this because of the additive nature of the phenotype and the low density of genotyped SNPs across the genome. We utilize the table browser tool simply to derive a list of all genes within modifier intervals, not to predict genes associated with the SNPs. In brief, for omics gene prioritization analyses we included all nomically significant intervals. For the final list of prioritized candidate genes (those listed in Figure 8B), we consider both the omics scores as well as whether the genes were in modifier intervals with genetic scores greater than 1.

Is there any published 3C, or similar chromatin conformation capture data that could be used to determine if the SNPs lie within the topological associated domain of the 'nearest' gene?

To date 3C or other chromatin conformation data has not been published for the fetal ENS. The complex temporal spatial development and lineage specific differentiation of the ENS are an important aspect to consider that would require generation of 3C or similar chromatin conformation capture from small cell numbers in the developing fetal intestine.

Were changes in expression levels of these genes assessed in Sox10dom animals, or other HSCR models?
The authors go on to examine the expression of SNP associated genes in a previously published scRNA-seq data set. The the top female associated SNPs (lines 296 onwards) are discussed and their location in the scRNA-seq data is determined. Why is the focus on female SNPs here when in the GEMMA analyses it seemed many SNPs were attenuated in females compared to males. The female SNP associated genes, Mcm3 and Isl1 whilst indeed expressed in ENCDCs', are also expressed in other populations at higher levels, but this is not discussed. Mcm3 was also detected in the 'BothSexNo15X5' GEMMA analysis, so this is not female specific. Isl1 is only expressed in ENCDC at 9.5, 10.5 and 11.5dpc, and only at very low levels. Is this meaningful? Did the authors further validate? Other genes seem more enriched in the ENCDCs, such as Uchl1, Limch1 and Arl8a but these are not discussed.

Modifier genes are expressed in wildtype animals and exert an effect when the mutation, in this case *Sox10*^{Dom}, is introduced. Thus we rely on wild-type expression data as described in the text that the scRNA-seq dataset derives from WT fetal gut. The genes we

assess in multiple datasets are contained within the defined modifier intervals. Because modifier genes are related to a particular gene mutation one cannot presume that other HSCR disease models would have the same modifiers or exhibit alterations in the same genes. Finally, because modifiers can arise from sequence variants that produce differences in protein amino acids, not all modifiers lead to changes in expression levels of a particular mRNA. This complexity is why quantitative trait analysis relies on aggregate evidence to identify the highest priority candidate genes and multimodal analysis as recommended by the Complex Traits Consortium (Abiola et al., 2003; DOI: 10.1038/nrg1206) . We now cite the Abiola study in our Discussion on Line 675.

The SNP near Mcm3 is detected in the combined analysis as well as in the female-specific analysis, but NOT in the male-specific analysis. This is why we state that this is female-specific: the lack of signal in males indicates it is at least sex-biased, if not specific to female sex.

Enrichment of gene expression in ENCDCs is not the only characteristic that would be expected of a modifier gene given the complex biological process that produces colonization of the fetal gut by ENCDCs. To better summarize the aggregate analysis, we now include a new panel in Figure 7, as requested by reviewer 1, that illustrates the combined scoring metrics for 19 candidate genes with modifier prioritization scores >0 and multiple lines of omics evidence.

It would be interesting to see trajectory analysis across the single-cell RNA-seq time course, scVelo or similar, and to use this to infer which future populations might be affected by the SNPs.

The scRNA-seq data set is a valuable resource for prioritization of candidate genes. However, there are multiple reasons why it would be unwise to use this for trajectory analysis in this study:

1. Trajectory analysis as a concept for scRNA-seq is still controversial in terms of its reliability. It relies on the organization of populations in the UMAP, which do not always correspond to future populations directly.
2. We include differential expression analysis across time as well as between cells to find differentially expressed genes across both time and cell type, this approach therefore eliminates the need for trajectory analysis.
3. The purpose of the analysis is to prioritize genes, and a velocity analysis, would not test anything beyond what we already have accomplished with our differential expression analysis.

There is no demonstration of co-expression of these modifier interval genes with Sox10, since this is the gene used in this particular model of HSCR it seems important to determine if other genes potentially contributing to the HSCR phenotype are expressed in the same cells.

Modifier genes exert an effect by altering the severity of a primary mutation. Given the complexity of the biological process that forms the ENS, which involves cell migration of ENCDC, signaling of ENCDCs with the gut environment and signaling between ENCDC progenitor cells we cannot limit the analysis to focus on only co-expression with Sox10. Focusing only on co-expression within the same cell may exclude important genes that are signaling from the environment to migrating ENCDCs. However, because *Dach1* exhibits the highest amount of omics evidence, we provide an inset below that shows co-expression of *Dach1* with *Sox10* in some progenitor cells marked by the arrowheads. We do qualify our response with the recent observation that SOX10 protein persists after the gene's

transcription is down-regulated (Avila et a., 2025 DOI 10.1016/j.jcmgh.2025.101590). Therefore, we caution that reliance on co-transcription may underestimate the effects of modifier interactions with the SOX10 protein.

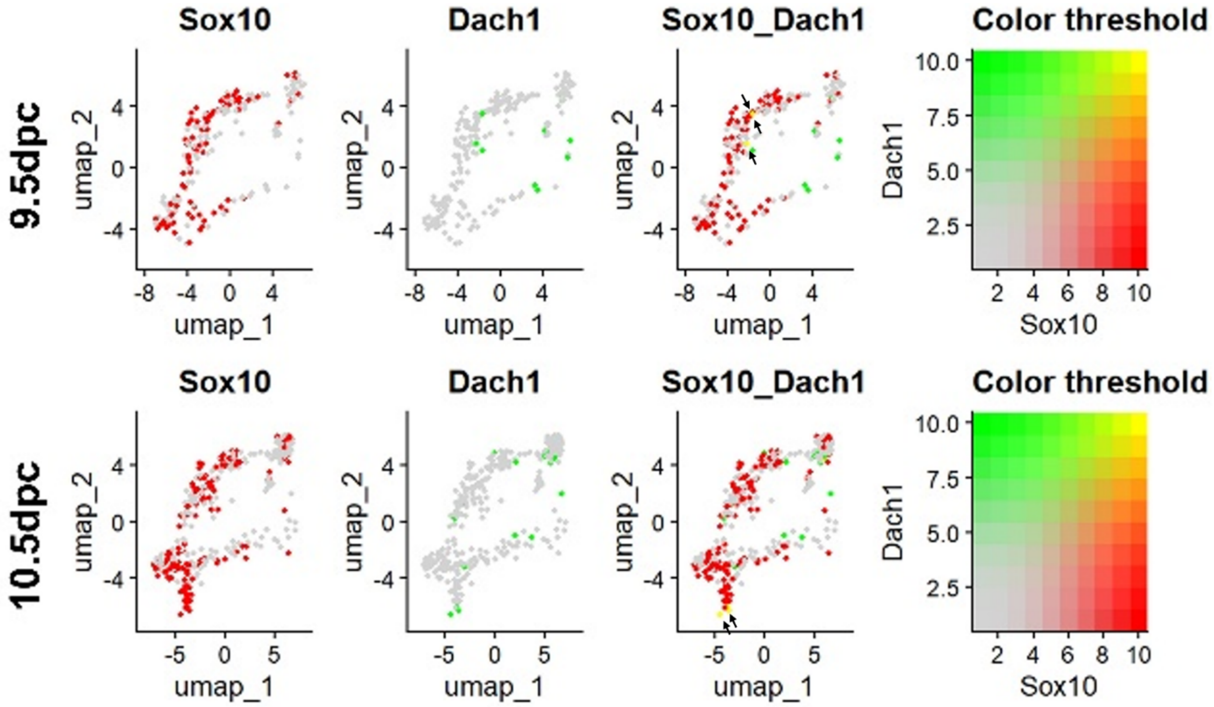

Table 4 could be a venn diagram, assuming there is some overlap of modifier intervals falling into the same category?

We have modified the text of the Table 4 legend to clarify the information shown. Because Table 4 displays numbers of genes within each modifier interval set that differ based on the GWAS from which they originated, the information cannot be collapsed into a single 2-dimensional Venn diagram. Each row corresponds to levels of prioritization of genes: first whether the genes are within modifier intervals at all, second whether the genes are expressed above a log-normalized expression threshold of 1 in pseudo-bulk of cell types at specific developmental time points, and third whether these genes are differentially expressed across cell type and developmental time point. Displaying this information in Venn diagram format would require either a multidimensional Venn diagram or seven separate Venn diagrams, which would make it much more difficult to compare values. We have retained Table 4 so that readers can quickly compare across rows.

The feature plots in Sup Fig 3 don't show anything more than what is shown in figure 4. It would be better here if the colocalization of candidate genes and Sox10 was shown on the same plot.

Actually, Reviewer 1 requested additional information in this figure, so we have retained to respond to that reviewer.

Regarding co-localization analysis, as we describe above, because modifiers for ENS development have the potential to be either genes that are within ENCDCs or genes in the fetal gut environment that are signaling to ENCDCs, focusing on co-expression alone could lead to exclusion of genes that are impacting the phenotype.

CellChat data needs justification and much more explanation. I understand the authors are looking for combinations of factors effecting severity of aganglionosis but what prompted them to examine ligand/receptor interactions over transcription factor co-binding or epistatic relationships, what was the hypothesis here?

We have expanded the introduction of this section and justification of CellChat with transitional sentences for explanation in the Results section which now states, "Communication between ENCDCs and the surrounding gut mesenchymne is an essential aspect for normal colonization of the developing fetal gut (Nagy and Goldstein 2017). To evaluate the potential of candidate genes in modifier intervals to either signal from the gut mesenchyme to ENCDCs or affect communication from ENCDCs to the surrounding gut microenvironment during bowel colonization, we applied CellChat. CellChat is a computational tool that quantifies communication between two cell groups using a reference database of over 3300 ligand receptor interactions and reliance on random permutation to estimate ligand-receptor communication among cell groups in scRNA-seq datasets (Jin et al. 2021; Jin et al. 2024). This adjustment is located on lines 372-381.

Our analysis evaluates ligand/receptor relationships on equal footing to other aspects of omics data since there is prior evidence that Ednrb is a primary gene for HSCR (both human and mouse data) and the protein it encodes is a receptor that is expressed in the developing enteric nervous system. There is no reason to assume that modifier genes are limited to being transcription factors.

We have modified this section to clarify that we examined whether both genes in ligand-receptor pairs could be located in modifier intervals.

Exactly what data is presented in Sup Fig 4A? Many predicted pairs, including Nrg1/Erbb3 have no symbol along the plot so what is their meaning? The excel sheet in supp table 12 is far from self-explanatory. In the text the authors highlight the predicted interaction of Nrg1-Erbb3, however this relationship has been well documented, whilst this provides something of a validation of the approach the fact that even known players are not significant in their analysis is somewhat worrisome.

The plot shown in Sup Fig 4A (now renumbered Sup Fig 5A) is output from CellChat that displays detected cell-cell communications plotted by developmental stage with symbols identifying the source GEMMA analysis run for the detected genetic modifier effect. *Nrg1-Erbb3* interaction is present in the lower panel of Sup Fig 4A for the 11.5dpc stage. CellChat output indicates cell communication via Nrg1-Erbb3 interaction from ENCDCs to telocytes on this plot. Since the reviewer believed we had overemphasized *Nrg1-Erbb3* signaling, we have trimmed back the emphasis on *Nrg1-Erbb3* in the results and equally mentioned the other genes that emerge from this filter including *Ednrb, Col1a1, Lgals9* and *Ppia*.

The value of this analysis is that it identifies predicted ligand-receptor interactions (per CellChat) between enteric neural crest-derived cells (ENS) and other cell types in scRNA-seq data from the developing gut dataset that are statistically significant.

We have added a legend to Supplementary Table 16 to clarify the columns generated by CellChat.

Differential expression of modifier interval genes in the migrating wavefront of enteric neural crest-derived migrating cells further prioritizes candidate genes.

In this section the authors turn to a bulk RNA-seq data set generated from migrating

enteric neural crest cells. I understand this was done to further refine the expression profile of their candidate modifier interval genes from the GEMMA analysis, in neural crest cells. However, they are not showing the expression levels of the genes they previously assessed in the scRNA-seq data e.g. Mcm3, Isl1, why is this? Furthermore, did they look back in the scRNA-seq data for the expression profile of genes that were differentially expressed in the bulk neural crest RNA-seq data?

Unfortunately, our initial description of the analysis process must not have been clear and was misunderstood. We have now expanded the text of this Results section to elaborate what was done and why. Genes that are enriched in the migrating wavefront of enteric neural crest-derived cells are more likely to be important for how far these cells migrate down the length of the gut as shown by the prior Stavely 2023 publication. Therefore, we used differential expression between wavefront cells and lagging cells to filter the gene lists from our modifier intervals. The point, as the reviewer correctly surmises, is to prioritize genes.

Regarding the second question, yes - we do look back at the scRNA-seq data for genes that are differentially expressed in the bulk neural crest data. This is shown in Figure 6 panel E and what is now labeled as Supplemental Figure 6 that provides UMAP plots of expression in ENCDCs.

We do not show data for Mcm3 or Isl1 on the bulk RNA-seq volcano plot as these genes were not detected as differentially expressed in the wavefront versus lagging populations, which would result in their positions being in the non-significant black region of the plot. Including these on the bulk RNA volcano plot has great potential to confuse readers.

When the authors say… 'We therefore prioritized genes within Sox10Dom aganglionosis modifier intervals that were differentially expressed in the ENCDC wavefront versus the lagging cells' does this mean they restricted the differential gene expression analysis to just a handful of genes, if so what were these genes? Or were DEG detected globally but then they looked for modifier interval genes within the DEGs? Either way, on the volcano plot in figure 5B and as mentioned in the text, the gene Ulch1 was among those downregulated in wavefront NC cells. Since this gene was also enriched in ENCDC population in the scRNA data surely it is worthy of further investigation.

Our initial presentation caused confusion on this aspect so we have restructured the information in this results section and added further details to clarify this aspect as elaborated on lines 401-412. We utilized differential expression results originating from the prior Stevely study. We overlapped those differentially expressed genes with the genes in our modifier intervals to identify any genes that exhibited differential expression either up- or down-regulated in the leading edge cells versus cells further back in the migrating population. The revised text now reads "we filtered for genes within modifier intervals that were differentially expressed in the ENCDC wavefront versus lagging cells (**Fig 6A**). We overlapped those differentially expressed genes with the genes in our modifier intervals to identify any genes that exhibited differential expression either up- or down-regulated in the leading-edge cells versus cells further back in the migrating population".

We agree that Uchl1 is among the genes that are interesting as depicted in the new figure panel 7B. However, because *Uchl1* is a well-characterized genes with known expression in differentiating neurons, it is not surprising that this gene is down-regulated in the wavefront and conversely up-regulated in differentiating neurons that lag behind as the gut is colonized. Since the goal of this analysis, is to prioritize candidate genes via a number of omics methods, and Uchl1 exhibits both genetic and omics evidence, we suggest that future studies should investigate candidate genes further in the Discussion.

What were the cutoffs for significance of DEGs? What are the genes shown to have greater p-value and logFC scores on the volcano plot in fig 5B.

The cutoffs were Bonferroni-adjusted p-value of 0.05 and log2fold change of <-0.5 or >0.5. Please see the Stavely 2023 study from which these data were sourced for further detail regarding data processing. The complete list of genes is associated with the Stavely et al publication that can be accessed under supplementary information at https://doi.org/10.1242/dev.201090 .

In the figure 5 legend; 'D Expression of Sox10, Elavl4, and Phox2a to label progenitor and neuronal cells, respectively.' It is not clear which gene(s) mark progenitor or neuronal cells. It reads like Sox10 and Elav4 mark progenitors, but Elav4 and Phox2a label the same cells so presumably these are neuronal? The plot from Sup Fig 5A might be more useful in the main figure 5.

For clarity, we have adjusted the figure legend to be more precise in language so this text now states "Expression of *Sox10*, labels progenitors (cyan) while *Elavl4* and *Phox2a* expression labels neuronal cells (salmon)." We have also moved the SupFig 5A panel into the main figure 6.

'However, wavefront upregulated genes are expressed in either a specific intermediate population (Col1a1, Tgfbi, Pdgfra)' please highlight the imtermediate population on the feature plots.

We have adjusted the text to read "are expressed in either an intermediate population (*Col1a1*, *Tgfbi*, *Pdgfra*) population marked by expression of the neuroblast gene Ascl ". We have added *Ascl1* as a neuroblast marker to Figure 5 panel D and we have emphasized the relevant intermediate populations with asterisks added to the figure panel.

Figure 5E, why are the downregulated genes not show across the time course like the upregulated genes are? Ptprg and dach1 do indeed increase in expression over the time course, but they are not refined to either progenitor or neuronal populations, which is of interest, but this is not elaborated on further. How do the up and downregulated genes look across the whole scRNA-seq data set, a plot of these similar to that in Fig 4C.

Cells left behind as the wavefront progresses undergo differentiation to form differentiated cells. Therefore, the genes enriched in the lagging population are expressed in the more differentiated neuronal populations marked by *Phox2b* and high levels of *Elavl4*.

The expression is not shown over time as it would not provide any greater insight in the main figures although we provide it below for the reviewer's enlightenment.

While we expect genes that are upregulated in the ENCDC migrating wavefront to be

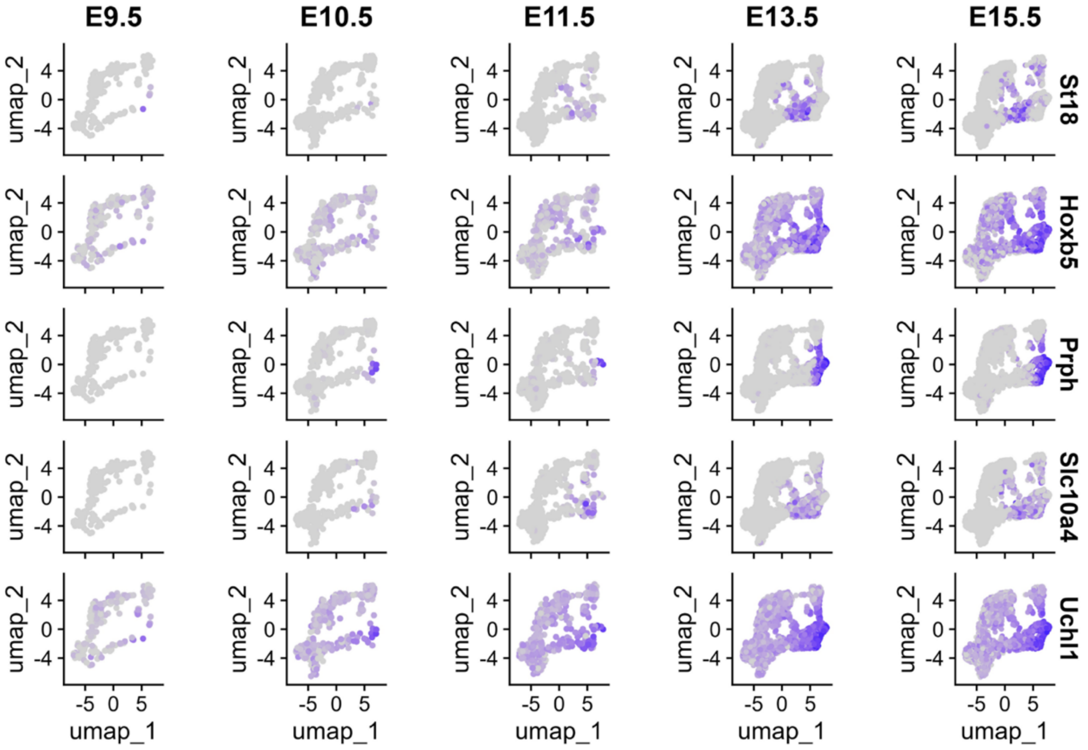

expressed in a small subset of ENCDCs in scRNA-seq and are likely playing a role in regulation of migration, this does not exclude the possibility that these genes are also playing roles in other surrounding gut cell types which may or may not affect the ENCDC migration through this alternative mechanism. We offer the below plots of their expression for the reviewer's interest below:

Wavefront down-regulated genes across all fetal gut scRNA-seq clusters:

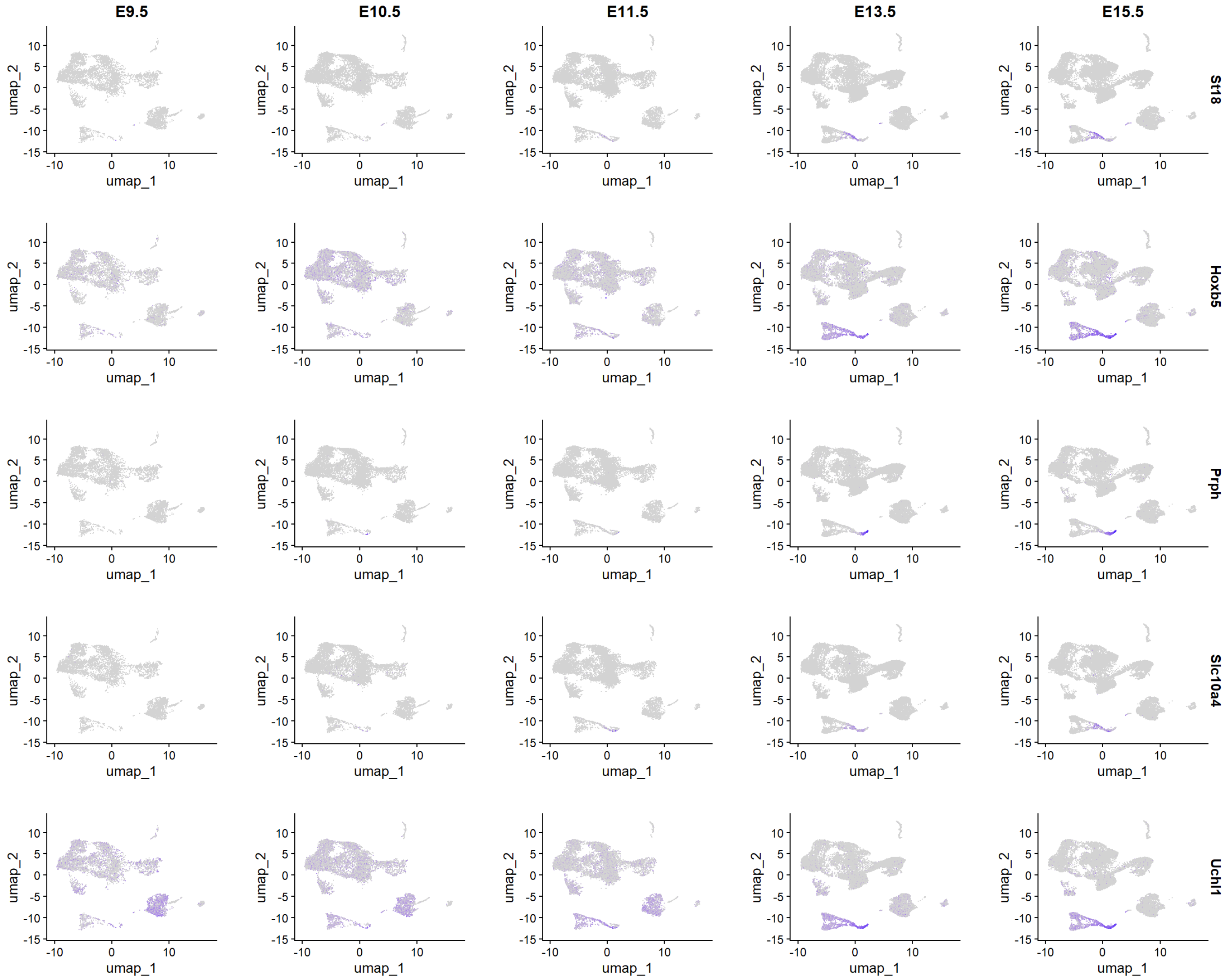

Wavefront up-regulated genes across all fetal gut scRNA-seq clusters:

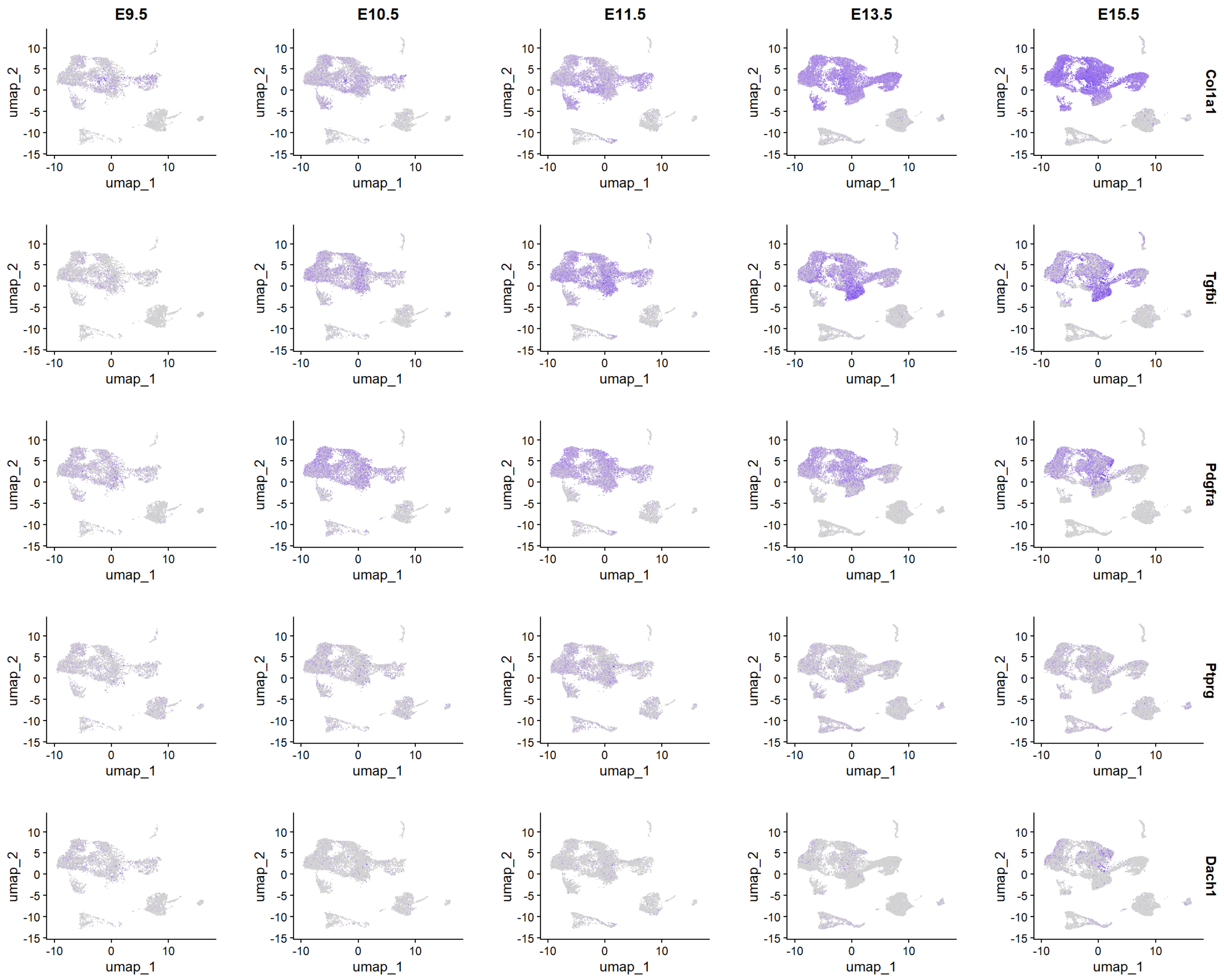

Table 5 could be removed, the GEMMA/GWAS categories in which each gene is found is shown by the legend in Fig 5E, for most genes.

To accommodate the reviewer's request, we have removed Table 5. Supp Table 16 provides this information in detailed format so that readers retain access to the information.

Evolutionarily conserved SOX10 binding sites within Sox10Dom modifier intervals overlap or are near genes differentially expressed in the migrating wavefront

The information in table 6 would be more interpretable presented as position weight matrices.

We appreciate the opportunity to clarify how this analysis was performed. The positions of conserved SOX10 binding motifs derived from a prior dataset (Gopinath et al., 2016 Additional Data file 4 linked at https://link.springer.com/article/10.1186/s12864-016-3167-3#Sec20) that had already located these conserved motifs on the genome assembly. As a result, we do not have PWM data for these sites.

Chromatin accessibility within enteric neuronal progenitors highlights putative regulatory regions within aganglionosis modifier intervals.

Here the authors generate single-nuclei ATAC-seq data to identify putative regulatory regions in which the modifier interval SNPs might be found. This is an appropriate experiment to characterise the genomic regions harbouring SNPs of interest. To this end they use the Phox2b mouse line to extract ENCDC's, it would be helpful to include a picture of CFP+ tissue.

We appreciate the opportunity to clarify our approach. The numbers of SNPs used in the genotyping panel are relatively sparse compared to the numbers of snATAC-seq peaks. We have identified the positions of differentially accessible chromatin from the snATAC-seq data to locate potential cis-regulatory sites within the modifier intervals.

At 16.5dpc when the snATAC-seq data was collected, the full length of the fetal gut is colonized so an overview picture would not be particularly informative from a visual perspective. We offer instead reference to images of fetal gut shown in our prior analyses of the Phox2b-CFP transgene published in Corpening et al 2008 (doi: 10.1002/dvdy.21498) where Figure 1 image panel D is comparable to what we observe at 16.5dpc.

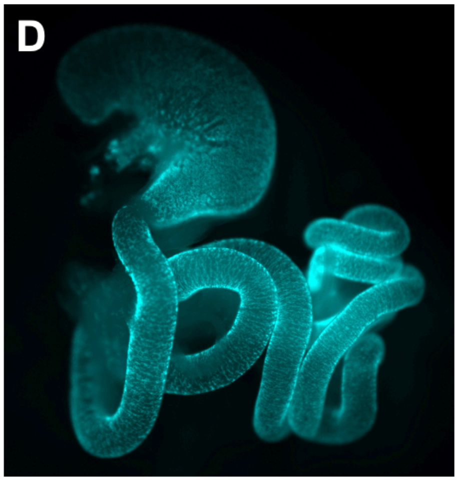

From how many gut's did they get 13431 nuclei? Were these from male or female or a mix of both?

This information was present in the supplemental methods of the original submission. To incorporate this detail into the main methods this information has been moved from into the main methods where we state: "Libraries for snATAC-seq were produced from two replicates with the first CFP-high and -low sorted populations produced from 6 pooled fetal guts and the second produced from 12 pooled fetal guts." So, these pools were a mixture of both sexes.

Following differential chromatin accessibility analysis it is convention to call these peaks 'differential accessible regions' (DARs), not DACs

As requested, we have revised the text to change the acronym so that DARs is used throughout.

The authors use transcriptome data from E15.5 mice (total foetal gut tissue) to annotate their single nuclei ATAC data from E16.5 mice ENCDC's. This is ok, but the authors should mention the caveat that they may be missing glial markers in this

annotation since E15.5-16.5 covers the glial transition phase, glial markers won't be present the in E15.5 transcriptome data. The authors could use promoter proximal peaks from their snATAC data to infer gene expression.

We appreciate the opportunity to clarify our analysis. The scRNA-seq data we utilize for gene prioritization in modifier intervals spans developmental stages 9.5-15.5dpc and is distinct from our previously published dataset at 15.5dpc (Avila et al., 2025; (https://doi.org/10.1016/j.jcmgh.2025.101590 see inset figure below responding to the query about Line 647). We did annotate the snATAC-seq data with 15.5dpc scRNA-seq data that originated from flow-sort isolated enteric nervous system progenitors. In that data, glial markers are present and expression of glial markers in the developing ENS is consistent with the prior report by Heather Young of glial markers (B-FABP at 11.5dpc and S100b at 14.5dpc) spanning from 11.5-16.5dpc (https://doi.org/10.1002/cne.10448). We have added a phrase elaborating this into the main methods section.

Figure 6A, the clusters should be annotated. The plots in 6A and 6B should be the same size, given their axis have the same scale.

The legend at the right side of the 6A UMAP shows the cluster labels. We have not used cluster labels in any aspect of the prioritization process and rely instead on expressed genes to avoid confusing readers.

As requested, the plot in 6B has been scaled to match 6A.

The scatter plot in 6C is very difficult to read. I would suggest either refining the legend by giving a motif with multiple occurrences a shape, or by showing an inset scatter plot of the motif enrichment from just one modifier interval category.

We understand the reviewers concern that scatter plot in Figure 6C is challenging to read. As recommended, we have inserted a scatter plot of motif enrichment for the Chr5 modifier interval which emphasizes the TF binding motifs in this the most significant and replicated modifier interval.

Line 438, Annotations did not deviate across groups except for Neuroblast2…. For precision, this should say 'Genomic annotations….' Since annotation could also refer to the cell type assigned to a given cluster.

We have revised this as requested.

Two of the conserved SOX10 binding motifs overlapped with modifier interval DAC. These were intronic to Ranbp17 (close to Tlx3; chr11:33400347-33400362) and Dach1 (chr14:97891443-97891462; Table 6, Supp. Table 17). This should be shown on the scatter plot in 6C

We appreciate the opportunity to clarify the information shown in Figure 6C. As stated in the figure legend, this plot is an enrichment plot for all transcription factor binding motifs detected in DARs present within modifier intervals. Given this, we are unable to show locations of these DARs on this plot in Figure 6C.

The complex filtering and comparisons of TF motifs in the snATAC data seems to just yield a list of TFs already known to play a role in ENS development. It would be more interesting/useful to discuss the predicted epistatic relationships between these factors.

We are delighted that the reviewer appreciates the value of the snATAC-seq data. We agree that the TF motifs identified in the DARs have been generally associated with ENS

development. It is important to recognize that the analysis we present suggests further that these transcription factors may have specific roles in affecting the ENCDC migration.

The focus of this study is on using the chromatin accessibility to further prioritize the candidate modifiers. Epistasis analysis at this stage, does not enable the main goal of the study and could confuse readers since actual function of these motifs will depend on accessibility and gene expression of these TFs that could be occuring much earlier in the migration process or even before the ENCDCs enter the fetal gut at 9.5dpc. Therefore we defer epistatic interactions of TFs for future analyses that are beyond the scope of the current study.

Line 462, 'Transcription factor (TF) binding is important for the transcription of genes…' this should state '….TF binding to CRE's is important for…..'

As requested, we have adjusted this wording in the revised submission.

Line 466, 'We focused on progenitors and neuroblasts because these are most similar to migrating ENCDCs' By what proxy are progenitors and neuroblasts most similar to migrating ENCDC's? Why is the focus now on migrating ENCDCs when the purpose of using the Phox2b line was to get a complete picture of ENCDCs when the gut was fully propagated with these cells?

We appreciate the opportunity to clarify this aspect of the study. ENS "progenitor cells" (salmon cluster in Figure 6B) are the least differentiated cells that give rise to the resulting lineages within mature enteric ganglia. Neuroblasts have begun to express early markers of neuronal differentiation (as shown by Avila et al., 2025 https://doi.org/10.1016/j.jcmgh.2025.101590 and others cited therein). These two groups of cells are the least differentiated and most similar to migratory ENCDCs at earlier stages. Since aganglionosis is a deficit in the migration phenotype, we expect the gene expression programs and therefore chromatin accessibility of these progenitors to be the most similar to wavefront cells. It must be recognized that even at 16.5dpc the snATAC-seq data required pooling fetal gut samples to obtain sufficient nuclei counts. and these would be diminished at earlier timepoints when cell numbers are dramatically lower which would contribute to lower-quality data. We have added additional text to this section on line 499 that clarifies this aspect stating "We focused on data from the progenitors and neuroblasts groups because these cell states are the most similar to migrating ENCDCs. Our snATAC-seq data collection at 16.5dpc enabled collection of sufficient numbers of nuclei to pursue accessibility studies that otherwise would have been extremely difficult with the low numbers of migrating ENCDCs at early stages of gut colonization."

Line 467, 'This approach examined both open chromatin in progenitors and neuroblasts as well as regions that are closed in differentiating enteric neuronal cells', how were differentiating enteric neuronal cells identified? These are not a separate cluster on the UMAP shown.

The progenitors, neuroblasts, and neuronal cells from the 15.5dpc data were assessed for a profile of many upregulated genes that together mark these specific transitional cell states. We used these and the predicted expression in the snATAC-seq data to label the cell types in the UMAP (Please refer to Sup Fig 7C). As these are developing cells along a trajectory of differentiation, we do not expect to see separate clusters. Developing enteric neuronal cells are those that are "neuronal" based on expression of multiple genes. For an example of some of the genes utilized please see Avila et al., 2025 (https://doi.org/10.1016/j.jcmgh.2025.101590) provided as a screenshot below for the reviewer:

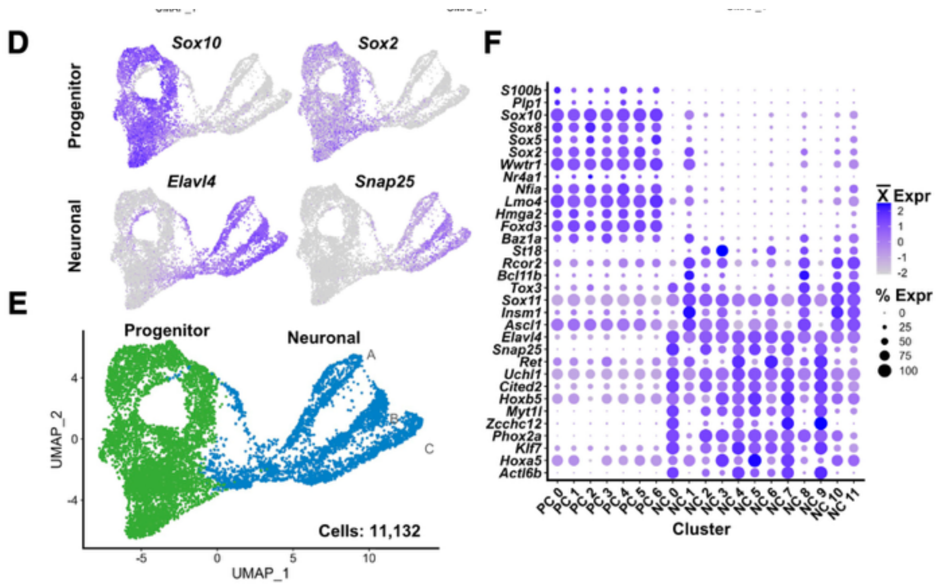

Figure 2. Profiled ENS populations from dual transgenic strategy capture ENCPs and multiple developing neuronal trajectories. (*A*) UMAP plots display similar scRNA-seq clusters for both genotypes prior to data merge (Supplementary Tables 1 and 2). (*B*) UMAP plots for each genotype after data merge shows overlap and similar cluster distributions (Supplementary Tables 3 and 4). (*C*) UMAP plot showing the main ENS populations comprised of 19 total clusters (Supplementary Tables 5 and 6). Other clusters consisting of Schwann-like cells (Cluster 13 panel B) and low-quality cells with high 18s rRNA gene expression, high mitochondrial and low RNA feature counts (clusters 1, 14, 10 panel B) were removed in this view. (*D*) UMAP representations of marker genes for progenitors (*Sox10*, *Sox2*) and developing neurons (*Elavl4*, *Snap25*) reveal the cluster distributions of these populations. A maximum normalized expression threshold was set to 2 in these images to highlight most expression of these genes. (*E*) UMAP of clusters shared by *Sox10*$^{+/+}$ and *Sox10*$^{Dom/+}$ classified by marker gene expression and GO analysis (Supplementary Tables 7 and 8) identified two main groups: progenitors (*green*) and neurons (*blue*). (*F*) Dot plot of known marker genes for ENS progenitors, neuroblasts, neurons, and glia per cluster.

We further clarify that at 16.5dpc, developing enteric neurons are not in a completely mature state as we and others (Memic et al 2018; Morarach et al., 2021)

ATAC data predicts cis-regulatory elements (CRE). The authors have focused on TF motif enrichment, which is useful, but they could take it further by selecting the TF motif enriched DAC, within modifier intervals, and asking what are the cognate putative target genes of predicted CREs and how might they be relevant to HSCR.

Formulating putative target genes of predicted CREs would require direct measurement of gene expression at 16.5dpc. We and others have 15.5dpc scRNA-seq data that we considered sufficiently close in age to utilize for annotation of clusters in snATAC-seq at 16.5dpc. However, lack of gene expression data at 16.5 precludes the exploratory analysis the reviewer is interested in seeing. Moreover, without extensive further data generation such putative target genes would not be validated and therefore speculative. We therefore have revised the text to state this as a future direction on lines 730-733 " Future work combining the snATAC-seq data with single cell gene expression profiles at 16.5dpc has the potential to identify putative target genes of predicted cis-regulatory elements that could emerge from combined snATAC-seq/scRNA-seq analytics, which may be relevant to HSCR or other gastrointestinal motility disorders that arise from disrupted ENS development. "

Prioritization of top aganglionosis modifier candidate genes across data modalities

The flowchart in figure 7 presents a nice summary of the analysis pipeline and the text provides some clarity regarding the analysis done. However, it is still somewhat

convoluted and difficult to entangle. For example, why were genes with a modifier interval score of 0 even included in the multiomics scoring system? A venn diagram might help demonstrate the findings from the dual scoring approach.
It would be interesting to know something about the shortlisted genes, e.g GO or IPA.

We appreciate the opportunity to clarify our analysis. The genetics score is applied to the modifier intervals that contain the genes, not the genes themselves. The genes inherit the genetics score based on location within intervals and the significance level of those intervals. Candidate genes whose modifier interval genetics scores are 0 are not included in the final candidate gene tables and are eliminated from further consideration. We realize the 0 value for modifier intervals P<0.05 was confusing in the text so we've modified this aspect and reworded as stated on lines 544-545 " For our modifier interval prioritization score, we assigned 1 to 3.5 points to each modifier interval and genes within these intervals inherited this score based on the following criteria . . . "

There are too few genes in the shortlist (only 30 genes) prior to filtering by genetics evidence to apply either GO or IPA (Ingenuity Pathway Analysis). Moreover, we prefer not to emphasize these genes that do not pass the filter for genetic evidence because they may not have any relationship to aganglionosis severity and therefore putting attention on them could confuse readers.

Candidate modifiers of aganglionosis related to human GI phenotypes

It is good that the authors have refined their gene lists by strain/background effects and then compared these to human data. However, how is the mouse genetic background relevant to human disease?

It has previously been shown that marked variability between affected HSCR siblings in human families can exist (Southard-Smith et al., 1999, DOI: 10.1101/gr.9.3.215, figure 5). Therefore, understanding how genetic background effects human HSCR disease is generally relevant and the first step in that process is mapping the modifier intervals and identifying the genes within the intervals that can be tested further.

We emphasize that the difference between mouse strains (genetic background) at the SNP level is how we were able to utilize GWAS effectively. The intervals containing SNPs that associate with the severity of phenotype are nearby genes that we have identified through our omics analysis. Looking at the homologous human genes in populations (the analysis we include in this submission, Table 7) has the potential to identify specific genes that exert similar effects.

It is only in this final section that Dach1 is discussed, and without any tangible link to HSCR or other ENS conditions it may not be appropriate to have these gene in the title of the paper, since the paper isn't really focussed on the Dach1finding, in fact this rather discredits all the other work the authors have performed.

We have revised the title to read "*Combinatorial multiomic analysis from a pedigree of Sox10*^{Dom} *Hirschsprung mice identifies high confidence candidate modifiers of Enteric Nervous System development* ".

The discussion section is somewhat repeative of the results and in other places seems to include some new data, or at least different number of genes in different categories, e.g. lines 606-608. It would greatly improve the readability and justification of approaches if the results and discussion section were combined.

While we appreciate the reviewers request to improve readability and we have modified a great deal of the text to address this request, we have retained the separation of Results and Discussion sections. We briefly summarize some results in the discussion to provide context and place the findings in the context of the field. This is important to elaborate how some of this challenging analysis provides greater insight. We have also elaborated on the accepted criteria for moving from genetic locus to identified gene as described by the Complex Traits Consortium (Abiola et al, 2003 article). Our submission is the first step in that process (moving from large modifier intervals to identification of candidate genes) which benefits from keeping the Results, which is packed with information, distinct from the Discussion so that readers are instructed about the multistep process of complex traits mapping.

Concluding comments:

The authors have conducted an extensive analysis of transcriptomes and chromatin accessibility data from mice ENS. Whilst vast, and as such applaudable, much of the analysis is not validated. The authors do recognise this, and modestly other limitations of the study, and I appreciate this paper has come to PLOS Computational Biology and therefore functional validation is not required/appropriate. However, in this era of interdisciplinary research it is not unreasonable to expect some in vivo, functional validation of key findings from computational studies e.g. expression of Dach1 in WT versus HSCR model mice gut, especially since this lab does have wet lab expertise as well as computational.

PLOS Computational Biology emphasizes computational studies with no requirement for accompanying biological wet bench experiments. Validation would require extensive experimentation that is far beyond the scope of the current analysis which already performs GWAS mapping, interval refinement, overlap of gene and chromatin expression patterns and develops a prioritization schema to identify the highest priority candidate genes. Further biological analysis is beyond the scope of the current effort and thus appropriate for future study.
