# Supplementary material for: Combinatorial multiomic analysis from a pedigree of Sox10Dom Hirschsprung mice identifies multiple high confidence candidate modifiers of Enteric Nervous System development
Source: PLoS Comput Biol. 2026 Jul 6;22(7):e1014424. doi: 10.1371/journal.pcbi.1014424 (PMC13372245; doi:10.1371/journal.pcbi.1014424)
Supplement: Attachment — Submitted filename: Benthal_etal_review round 2_Response_Upload.pdf [file pcbi.1014424.s043.pdf]

Response to Review
We again greatly value the time and attention the reviewers have given to our submission and appreciate the opportunity to revise our submission. Our responses are below in **blue** with prior responses in italics so the latest reply in **blue** is easily located. To address reviewer concerns we have expanded the main text with information regarding co-expression of *Sox10* candidate modifiers with *Sox10*, added an additional main figure, and included an additional supplementary figure. We believe this additional information on the potential for *Sox10-Dach1* interaction further strengthens the submission and broadens the articles' appeal to readers who are generally interested in gene expression patterns in the developing ENS.

Original Comment: Examining co-expression of Dach1 with known ENS transcription factors (for example Sox10, Phox2b, Ret, Ednrb) in the Zhao scRNA seq dataset, for instance via a correlation heatmap across ENCDC subclusters.

R1 Response: *We agree that co-expression of Dach1 with Sox10 is an important aspect and we have utilized screening for candidate gene expression concurrently in the ENS as a means to prioritize potential modifier genes. We offer expression feature plots for Dach1, with Sox10, Phox2a, and Hoxb5 in Figure 5, which are relevant transcription factors for ENS development. These plots show that Dach1 is expressed in progenitor populations that are concurrently expressing high levels of Sox10 transcription. However, we are aware that SOX10 protein persists in early developing neurons (Avila et al., 2025 DOI: 10.1016/j.jcmgh.2025.101590) therefore strictly relying on RNA levels in the same cells to prioritize candidate modifiers does not take into consideration the possibility that protein interactions may be affecting the severity of aganglionosis as ENS progenitors undergo differentiation. To address this request, we provide a heatmap in our response that shows the overlap with Sox10 since that is the gene mutation for which we are working to map modifier loci. As expected, since our criteria for candidates was that genes be expressed in the ENS and surrounding gut cells there are some cells that exhibit co-expression. Analysis of co-expression for other factors and receptors like Ednrb likely will confuse the reader and distract from the main focus of the article on combinatorial analysis to prioritize biologically relevant candidate modifier genes of $Sox10_{Dom}$ during ENS development*

R2 New Comment: I thank the authors for providing the UMAPs and associated co-expression heatmaps. From the provided image (below) it seems hardly any cells co-express Sox10 and Dach1(Yellow cells). This is concerning. Also the authors argument –" *therefore strictly relying on RNA levels in the same cells to prioritize candidate modifiers does not take into consideration the possibility that protein interactions…"* is not convincing. For protein interactions to happens the RNA has to be present!
This new data actually makes the interpretation harder to substantiate regarding the role of Dach1. The authors should carefully look at their analysis to check if Dach1 has been mapped properly in the single cell datasets they are looking at given the very low proportions of cells (green) where it is detected. Please generate a side-by-side violin plot of Sox10 and Dach1 to demonstrate levels of expression in same cell types.

Given this data it is even more pertinent the authors look at co-expression of other key ENS markers like Phox2b, Ednrb, Ret in Dach1 positive cells. Also the authors seem to have a low opinion about the intellect of the readers they will "get confused" if they see co-expression data with key ENS markers!

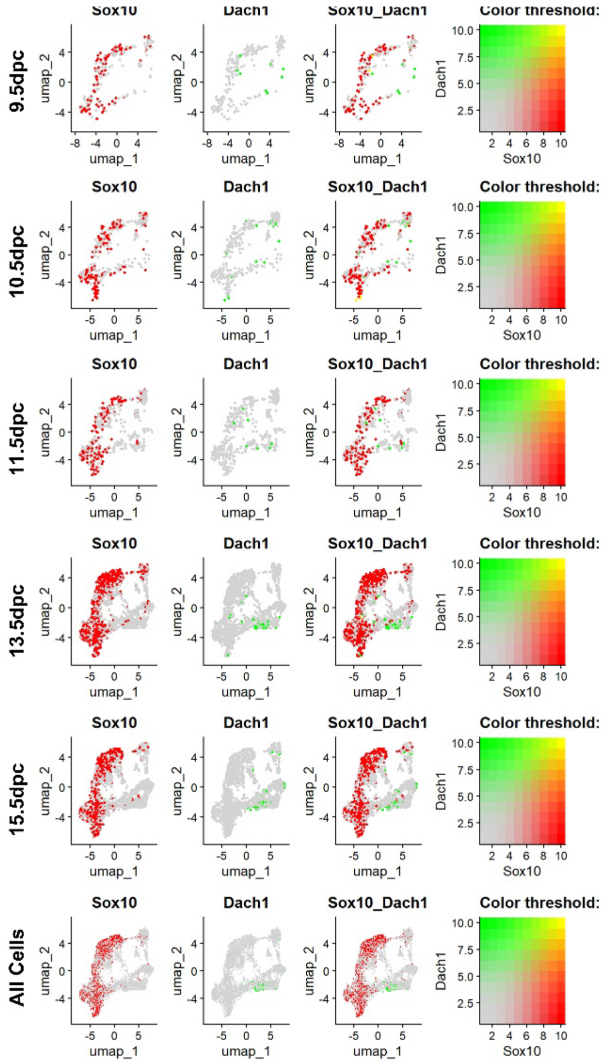

R2 New Response: We regret that we did not fully elaborate on our prior response regarding persistence of SOX10 protein which drew reviewer concern regarding our statement –" *therefore strictly relying on RNA levels in the same cells to prioritize candidate modifiers does not take into consideration the possibility that protein interactions…"* . We appreciate the opportunity to further expand on this aspect here. In the recent Avila et al publication (PMID: 40701368 ;DOI: 10.1016/j.jcmgh.2025.101590), we demonstrated that SOX10 protein persists in ENS progenitor populations that have begun to down-regulate *Sox10* transcription (only faintly Sox10-YFP+ or undetectable Sox10-YFP+) and are upregulating *Phox2b* transcription (emerging Phox2b-CFP+) and exhibit HuC/D immunoreactivity in Figure 1A. We now include this figure panel below for the reviewer's convenience. This down-regulation of Sox10 transcription while SOX10 protein persists in early emerging neurons implies that relying strictly on RNA expression for co-localization with potential candidate modifiers may miss interactions that are mediated by SOX10 protein in transitional cell states as the *Sox10* gene down-regulates. We did not mean to imply that SOX10 protein is present in cells without prior transcription. Our intent was to make the reviewer aware presistance of SOX10 protein occurs and could complicate strict reliance on transcriptional data for co-expression.

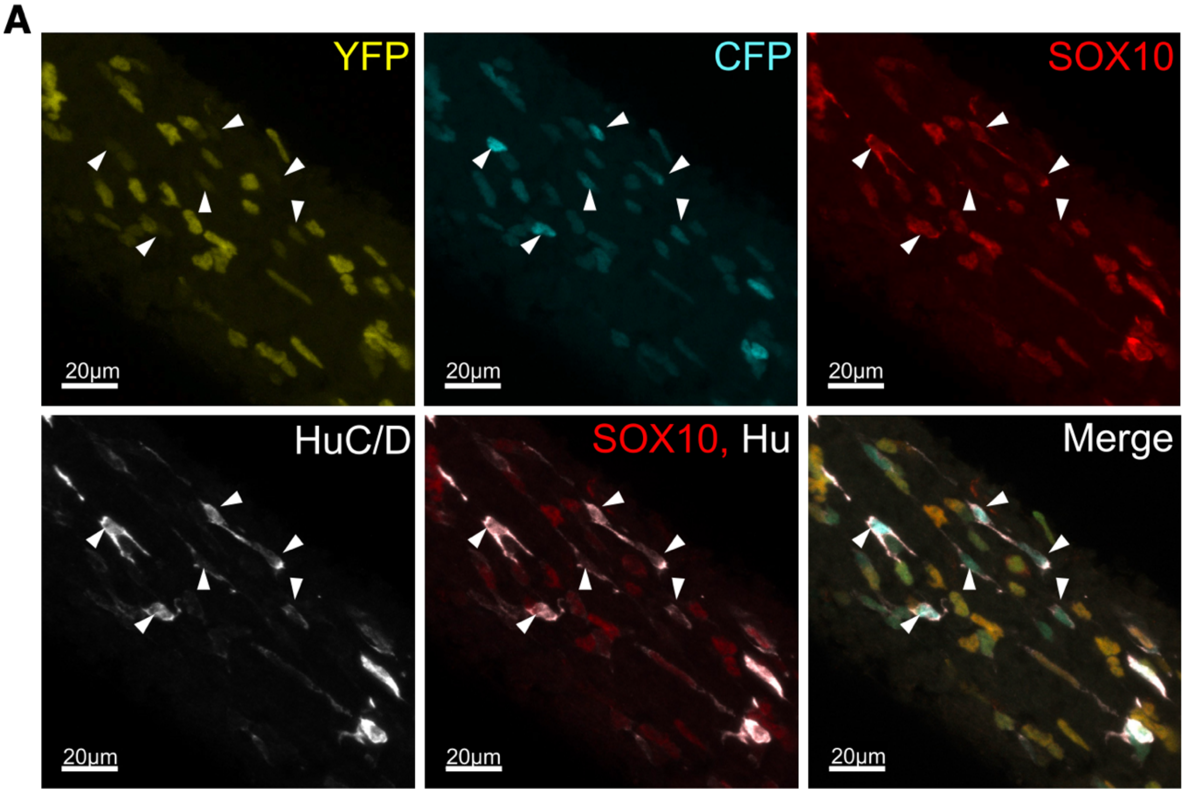

We further thank the reader for the recommendation to more closely evaluate co-expression of *Dach1* and *Sox10*. We have done as the reviewer recommended with new analysis that we offer below and now include in the article*.* For clarity, in our initial response to this request, we neglected to standardize expression levels of *Sox10 and Dach1* on the UMAPs we provided. In early stages of ENS development, *Sox10* is expressed at higher levels in progenitors than *Dach1* and Seurat (the R package we are using for analysis) in its co-expression plots does not adjust for the different levels of expression for each gene. Therefore, we re-evaluated each separate timepoint and compared levels of expression in FeaturePlots of *Sox10* and *Dach1* separately, side by side (See R_2 Figure A below). This comparison shows that in progenitors that express *Dach1*, *Dach1* is typically expressed at about half the level as Sox10 is typically in progenitor cells. Therefore, to better visualize co-expression, we used Seurat's FeaturePlot function argument "max.cutoff" set to 0.5 normalized expression (See R_2 Figure B below). This highlights cells that lowly express *Dach1* in the progenitors that are more visible when viewing the total expression of each dataset alone. Co-expression (yellow cells that indicate presence of *Sox10* transcripts with *Dach1* transcripts in the same cell) is most apparent at 11.5dpc and 13.5dpc when migration is ongoing. **We have now included a new figure that illustrates this co-expression (New Figure 9) in the main text.**

Figure R_2 Figure A: comparison of *Sox10* and *Dach1* expression in Seurat FeaturePlots generated from Zhao et al., 2022 data across ENS developmental stages.

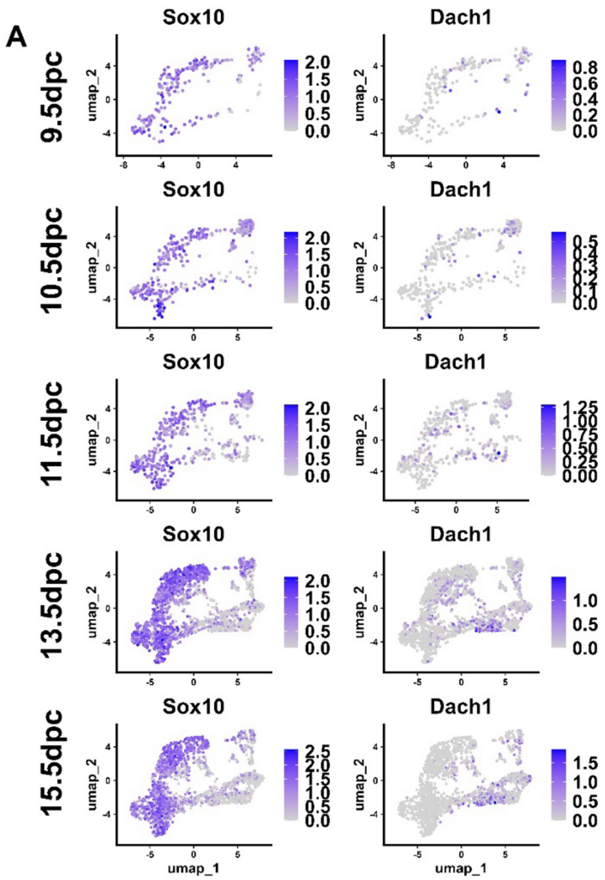

A) Normalized expression **not** limited by "max.cutoff=0.5" is shown in FeaturePlots for Zhao et al. "neural crest" scRNA-seq data split by developmental timepoint (rows) for *Sox10* and *Dach1* (columns).

Figure R_2 Figure B: Feature plots with Seurat FeaturePlot function argument “max.cutoff” set to 0.5 normalized expression generated from Zhao et al., 2022 data. The panel for 13.5dpc is now included as one element of a new Figure 9 in the main text along with panels for *Phox2b, Ednrb* (known modifiers of Sox10), *Elavl4* that shows *Dach1* increased frequency of expression in neurons, well as *Pdgfra1,* and *Tbx2* that are logical candidates from Table 6 based on ENCDC expression and presence of SOX10 binding motifs. These modifications address the reviewers request and provide informative markers of distinct cell types and shown known or candidate modifiers of *Sox10*^{Dom} aganglionosis extent.

B

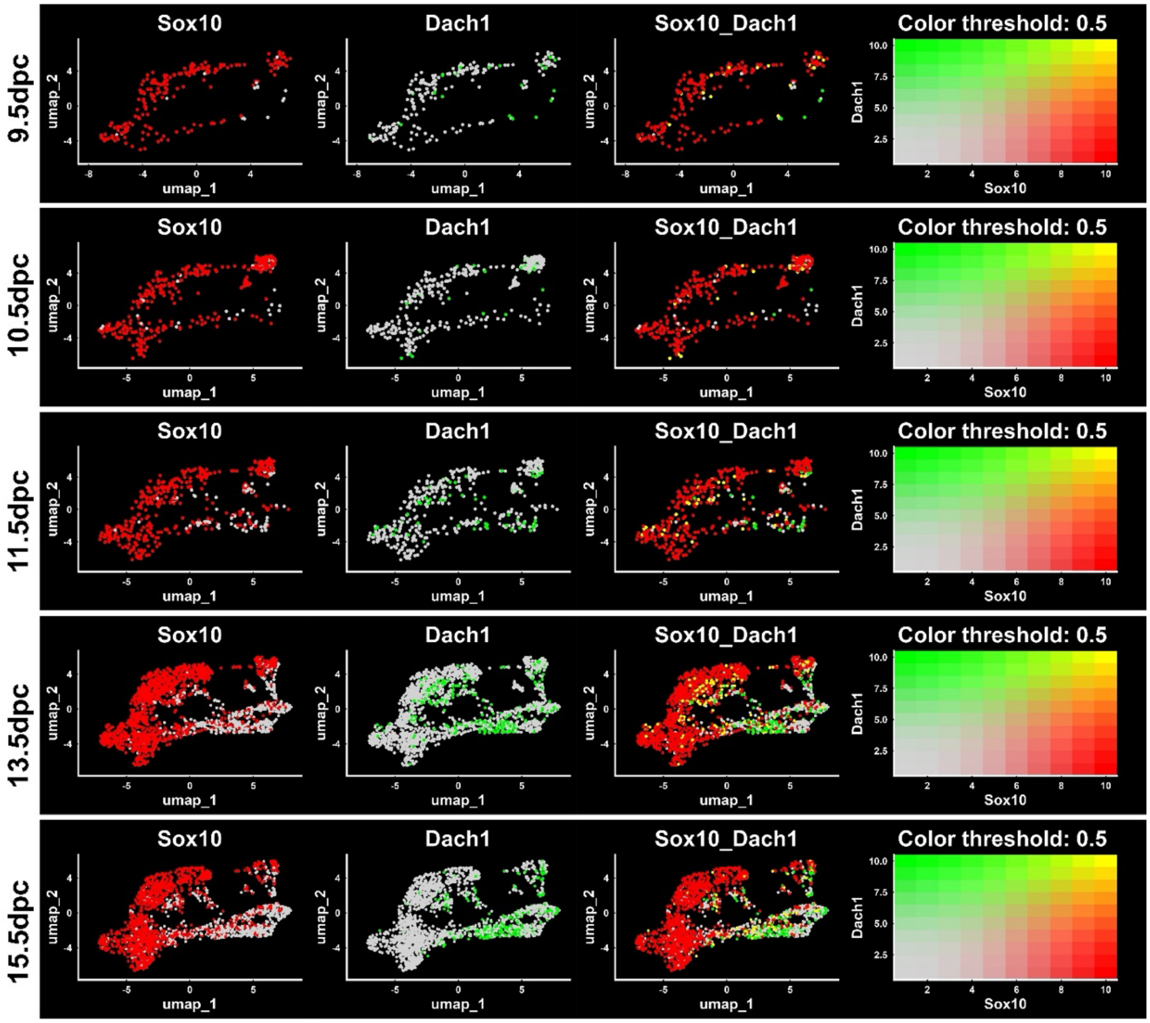

B) Co-expression FeaturePlots generated using the argument “max.cutoff=0.5” showing expression of *Sox10* (first column) with *Dach1* (second column), co-expression of *Sox10* and *Dach1* (third column), and the color threshold (fourth column) split by developmental timepoint (rows).

The reviewer requested violin plots for *Dach1* along with standard ENS marker genes such as *Sox10*, *Ednrb*, *Ret*, *Phox2b*. We provide these below along with a feature plot for *Elavl4,* which is a well-established pan-neuronal marker that encodes HuD (See R_2 Figures F,G below). This figure does show cells that express *Dach1* in progenitors and neuronal cells, with more frequent expression in neuronal cells. We have now incorporated this violin plot overview as Supplementary Figure 8 into the article so this information for *Sox10* and the highest priority candidate genes for interaction with *Sox10* that exhibit a priority score of $\geq$ 4 (*Dach1, Col1a1, Pdgfra, Tbx2*) is available to readers.

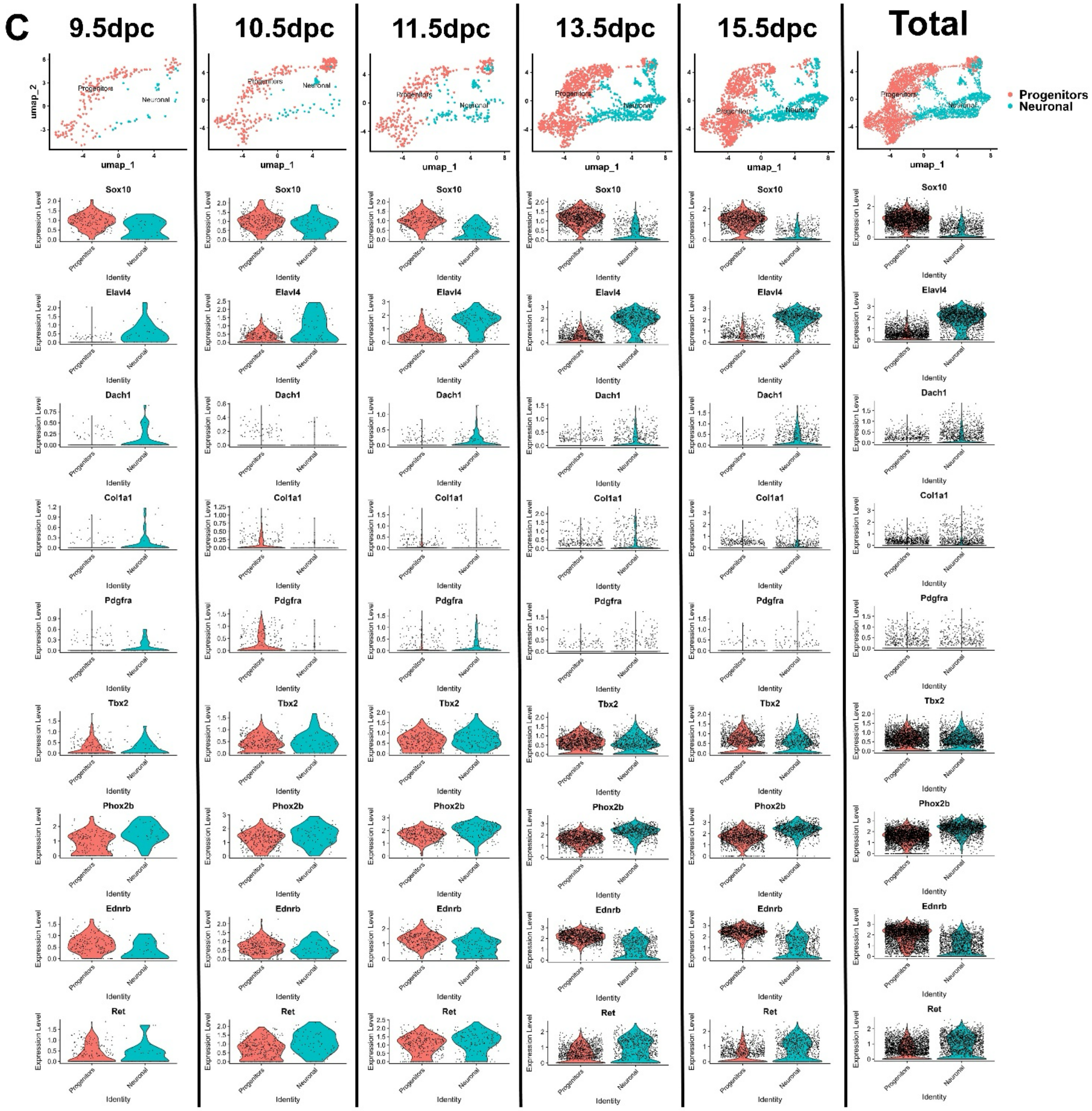

C) Top column shows the Zhao et al. ENCDCs split by timepoint (columns) in chronological order and all cells (rightmost column) colored by cell state (progenitor, neuronal). Violin plots showing expression of *Sox10*, *Elavl4*, *Dach1*, *Col1a1*, *Pdgfra*, *Tbx2*, *Phox2b*, *Ednrb* and *Ret* by cell state corresponding to each timepoint in the top row.

The reviewer requested co-expression plots for *Sox10* as well as *Ednrb*, and *Ret*. We offer these plots below and have included a new Figure 9 added to the manuscript that incorporates *Ednrb*, *Phox2b*, and the genes our study indicates are high priority candidates with scores of 4+ from Table 6. We have also added *Elavl4* to this list, which labels developing neurons to aid readers in recognizing these populations. We employ the same “max.cutoff=0.5” argument to the co-expression FeaturePlots to better visualize cells that express *Dach1* at lower levels and provide these below (See R_2 Figures D-H below). In the co-expression plots, *Dach1* expression overlaps with each of these genes in both progenitor and neuronal populations, with the most prominent expression in neuronal cells, particularly at later developmental stages. For full transparency we note that in the supplemental figure provided in the revised article, we have not included plots for *Ret.* We have no evidence for *Ret* affecting *Sox10*^{Dom} aganglionosis as a modifier in our crosses. This study is focused on candidate modifiers of *Sox10*^{Dom} and we have difficulty justifying inclusion of Ret because it is not a modifier nor does it mark a particular lineage like *Elavl4* does.

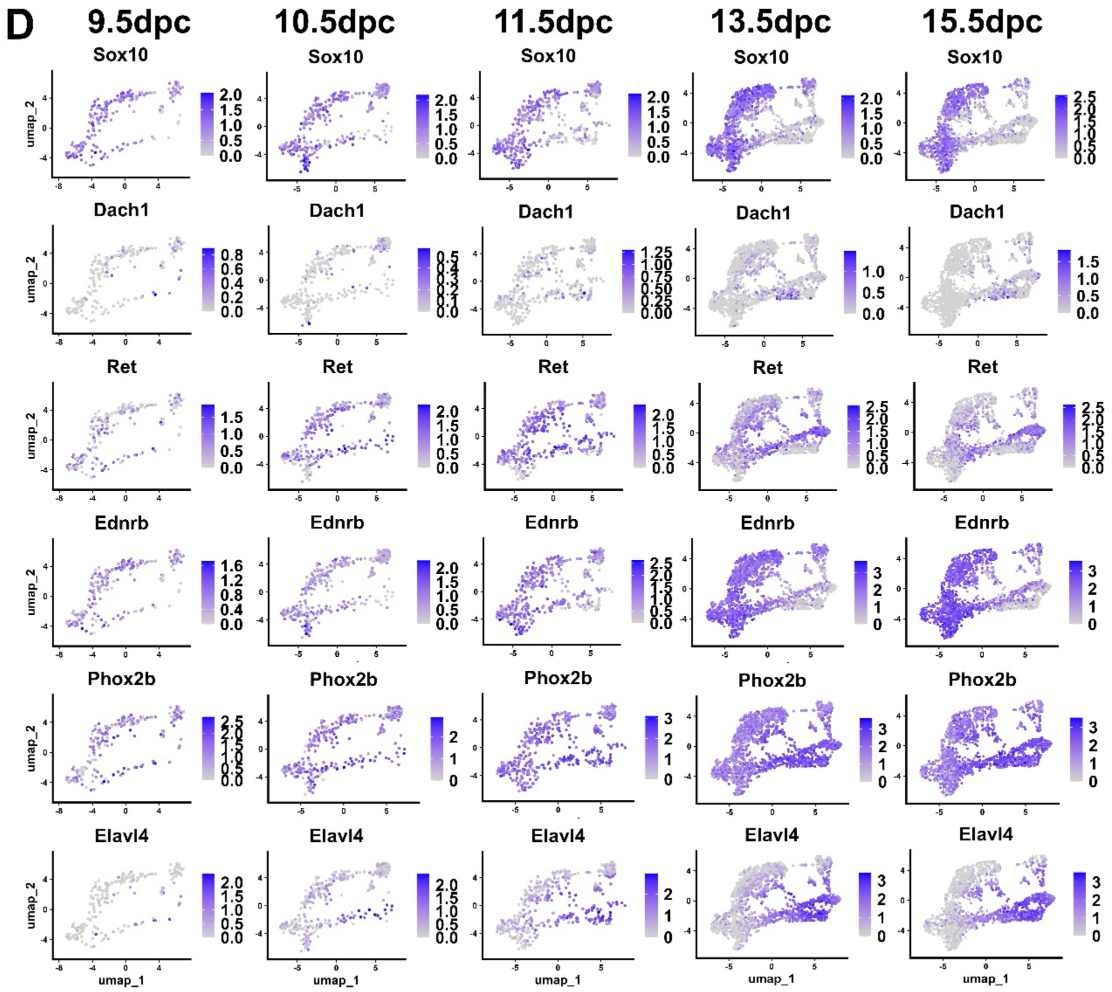

D) FeaturePlots showing normalized expression of *Sox10*, *Dach1*, *Ret*, *Ednrb*, *Phox2b*, and *Elavl4* (rows) **not** limited by “max.cutoff=0.5”, split by developmental timepoint (columns). **E-H**) Same as in B but for *Ret* (E), *Ednrb* (F), *Phox2b* (G), and *Elavl4* (H) showing co-expression with *Dach1* using the “max.cutoff=0.5” argument.

As requested we offer the below panels in our response to review.

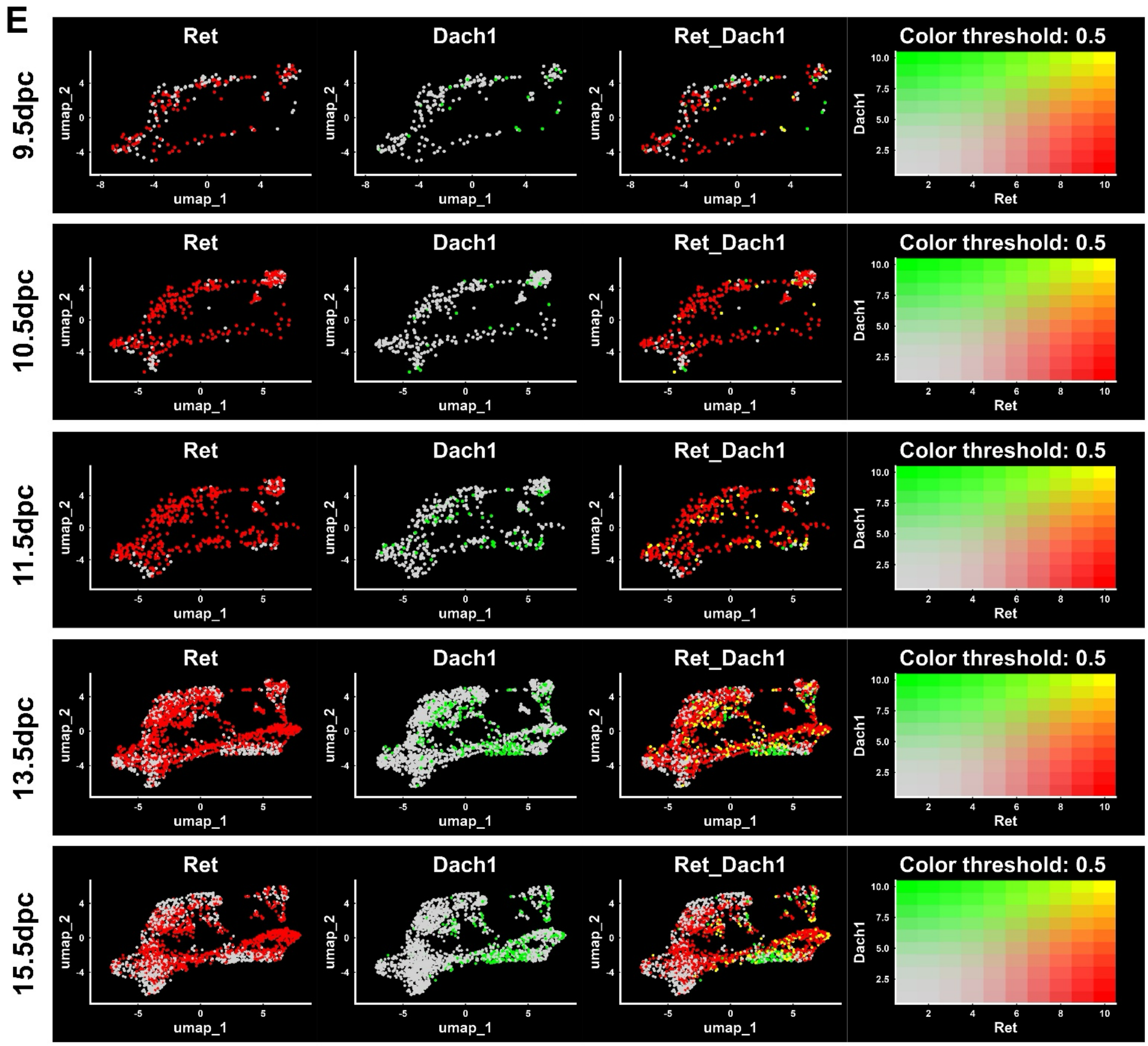

F

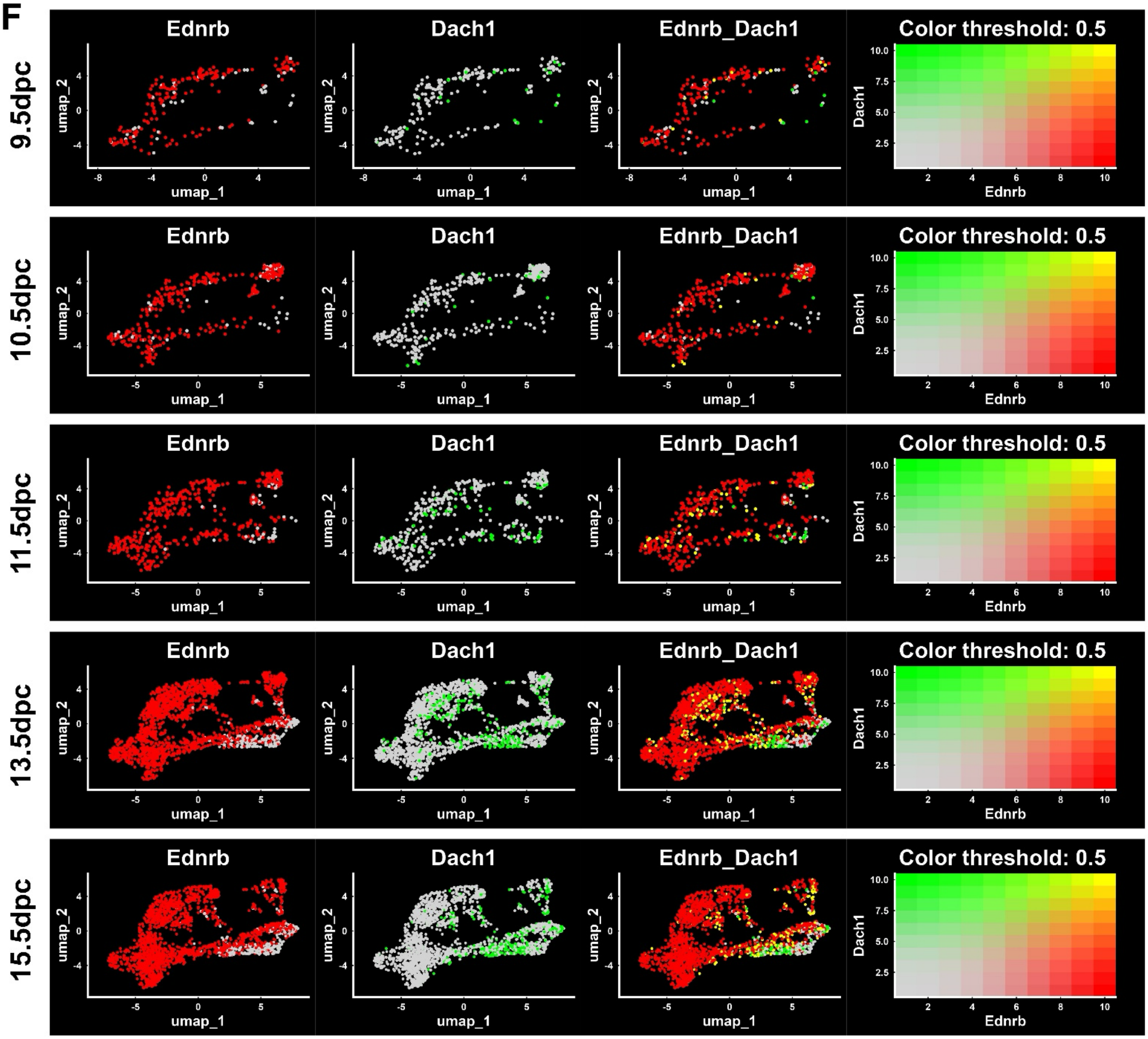

9.5dpc
10.5dpc
11.5dpc
13.5dpc
15.5dpc
Ednrb
Dach1
Ednrb_Dach1
Color threshold: 0.5
umap_1
umap_2
Dach1
Ednrb

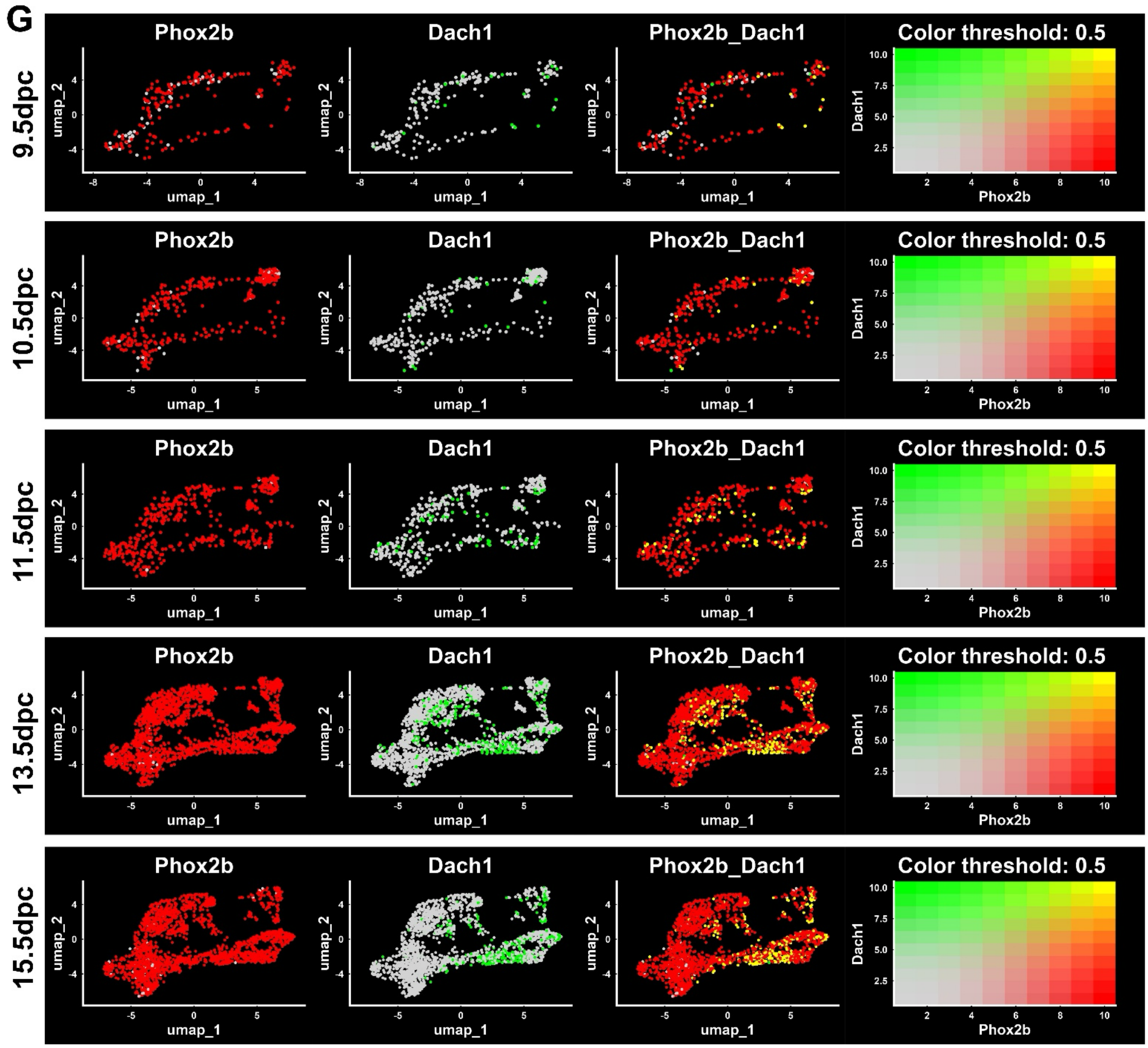
G
9.5dpc
10.5dpc
11.5dpc
13.5dpc
15.5dpc
Phox2b
Dach1
Phox2b_Dach1
Color threshold: 0.5
umap_1
umap_2
Dach1
Phox2b

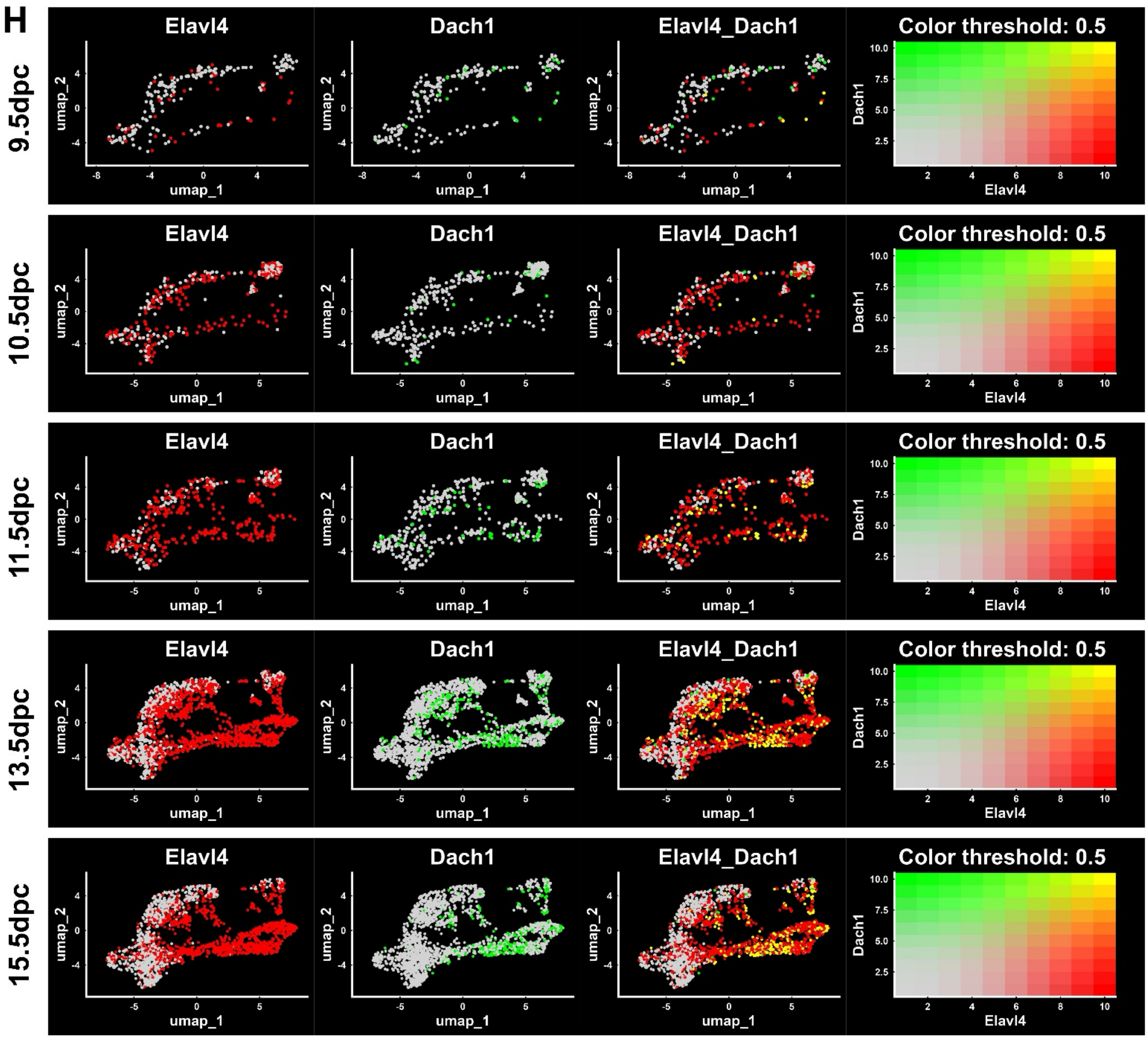

Original Comment: *In Manhattan plots, label the chromosomal positions of the key genes (Phox2b, Ednrb, Dach1) directly on the plots to guide readers.*

Initial Response: *We appreciate the reviewer's enthusiasm for the genes that we have identified through a combined analysis of multiple data modalities. However, we are concerned that overlaying the genes on the Manhanttan plots could lead to misinterpretation of those GEMMA data, which are strictly association with SNPs across the genome. We detect Dach1 through a combination of genetics and complementary omics analysis. Adding gene labels to chromosomal positions could cause some naïve readers to think that we directly evaluated association with those genes, which was not the case.*

<u>R2 New Comment:</u> Again the authors seems to have a very low opinion of the intellect of the readers who will read their papers. It is a very standard practice to mark genes on a Manhattan plot even if the genes are “suggested” or arrived at from multiple lines of evidence. If the reader is “naïve” he or she will look up how this was achieved. A paper should also educated readers. Please include gene names.

Our low SNP genotyping density is the reason for the extensive omics analysis that narrows down our list of candidates. As the reviewer has requested we have added the gene names to Figures 2 and 3. We have annotated with change with details in the figure legend stating that these genes are added from the final list at the end of the paper with the legend now states “Gene positions shown are approximate and are candidate modifiers derived from omics evidence summarized in Table 6 after the prioritization process and are not meant to indicate genome wide significant SNPs at these genes.”
