# Supplementary material for: Combinatorial multiomic analysis from a pedigree of Sox10Dom Hirschsprung mice identifies multiple high confidence candidate modifiers of Enteric Nervous System development
Source: PLoS Comput Biol. 2026 Jul 6;22(7):e1014424. doi: 10.1371/journal.pcbi.1014424 (PMC13372245; doi:10.1371/journal.pcbi.1014424)
Supplement: Attachment — Submitted filename: Benthal_etal_review round 2.docx [file pcbi.1014424.s041.docx]

**Original Comment:** Examining co-expression of Dach1 with known ENS transcription factors (for example Sox10, Phox2b, Ret, Ednrb) in the Zhao scRNA seq dataset, for instance via a correlation heatmap across ENCDC subclusters.

**Response:** *We agree that co-expression of Dach1 with Sox10 is an important aspect and we have utilized screening for candidate gene expression concurrently in the ENS as a means to prioritize potential modifier genes. We offer expression feature plots for Dach1, with Sox10, Phox2a, and Hoxb5 in Figure 5, which are relevant transcription factors for ENS development. These plots show that Dach1 is expressed in progenitor populations that are concurrently expressing high levels of Sox10 transcription. However, we are aware that SOX10 protein persists in early developing neurons (Avila et al., 2025 DOI: 10.1016/j.jcmgh.2025.101590 ) therefore strictly relying on RNA levels in the same cells to prioritize candidate modifiers does not take into consideration the possibility that protein interactions may be affecting the severity of aganglionosis as ENS progenitors undergo differentiation. To address this request, we provide a heatmap in our response that shows the overlap with Sox10 since that is the gene mutation for which we are working to map modifier loci. As expected, since our criteria for candidates was that genes be expressed in the ENS and surrounding gut cells there are some cells that exhibit co-expression. Analysis of co-expression for other factors and receptors like Ednrb likely will confuse the reader and distract from the main focus of the article on combinatorial analysis to prioritize biologically relevant candidate modifier genes of Sox10Dom during ENS development*

**New Comment:** I thank the authors for providing the UMAPs and associated co-expression heatmaps. From the provided image (below) it seems hardly any cells co-express Sox10 and Dach1(Yellow cells). This is concerning. Also the authors argument –“ *therefore strictly relying on RNA levels in the same cells to prioritize candidate modifiers does not take into consideration the possibility that protein interactions…”* is not convincing. For protein interactions to happens the RNA has to be present!

This new data actually makes the interpretation harder to substantiate regarding the role of Dach1. The authors should carefully look at their analysis to check if Dach1 has been mapped properly in the single cell datasets they are looking at given the very low proportions of cells (green) where it is detected. Please generate a side-by-side violin plot of Sox10 and Dach1 to demonstrate levels of expression in same cell types.

Given this data it is even more pertinent the authors look at co-expression of other key ENS markers like Phox2b, Ednrb, Ret in Dach1 positive cells. Also the authors seem to have a low opinion about the intellect of the readers they will “get confused” if they see co-expression data with key ENS markers!

**Original Comment:** *In Manhattan plots, label the chromosomal positions of the key genes (Phox2b, Ednrb, Dach1) directly on the plots to guide readers.*

**Response:** *We appreciate the reviewer’s enthusiasm for the genes that we have identified through a combined analysis of multiple data modalities. However, we are concerned that overlaying the genes on the Manhanttan plots could lead to misinterpretation of those GEMMA data, which are strictly association with SNPs across the genome. We detect Dach1 through a combination of genetics and complementary omics analysis. Adding gene labels to chromosomal positions could cause some naïve readers to think that we directly evaluated association with those genes, which was not the case.*

**New Comment:** Again the authors seems to have a very low opinion of the intellect of the readers who will read their papers. It is a very standard practice to mark genes on a Manhattan plot even if the genes are “suggested” or arrived at from multiple lines of evidence. If the reader is “naïve” he or she will look up how this was achieved. A paper should also educated readers. Please include gene names.
