# Supplementary material for: Combinatorial multiomic analysis from a pedigree of Sox10Dom Hirschsprung mice identifies multiple high confidence candidate modifiers of Enteric Nervous System development
Source: PLoS Comput Biol. 2026 Jul 6;22(7):e1014424. doi: 10.1371/journal.pcbi.1014424 (PMC13372245; doi:10.1371/journal.pcbi.1014424)
Supplement: Peer review history

# Peer review: Decision Letter 0

**DOI:** 10.1371/journal.pcbi.1014424.r001

**Authors:** Mark Ziemann, Jean Fan

13 Jan 2026

PCOMPBIOL-D-25-02281

Combinatorial multiomic analysis from a pedigree of Sox10Dom Hirschsprung mice implicates Dach1 as a modifier of Enteric Nervous System development

PLOS Computational Biology

Dear Dr. Southard-Smith,

Thank you for submitting your manuscript to PLOS Computational Biology. After careful consideration, we feel that it has merit but does not fully meet PLOS Computational Biology's publication criteria as it currently stands. Therefore, we invite you to submit a revised version of the manuscript that addresses the points raised during the review process.

Please submit your revised manuscript by Mar 15 2026 11:59PM. If you will need more time than this to complete your revisions, please reply to this message or contact the journal office at ploscompbiol@plos.org. When you're ready to submit your revision, log on to https://www.editorialmanager.com/pcompbiol/ and select the 'Submissions Needing Revision' folder to locate your manuscript file.

Please include the following items when submitting your revised manuscript:

* A letter that responds to each point raised by the editor and reviewer(s). You should upload this letter as a separate file labeled 'Response to Reviewers'. This file does not need to include responses to formatting updates and technical items listed in the 'Journal Requirements' section below.

* A marked-up copy of your manuscript that highlights changes made to the original version. You should upload this as a separate file labeled 'Revised Manuscript with Track Changes'.

* An unmarked version of your revised paper without tracked changes. You should upload this as a separate file labeled 'Manuscript'.

If you would like to make changes to your financial disclosure, competing interests statement, or data availability statement, please make these updates within the submission form at the time of resubmission. Guidelines for resubmitting your figure files are available below the reviewer comments at the end of this letter

We look forward to receiving your revised manuscript.

Kind regards,

Mark Ziemann

Academic Editor

PLOS Computational Biology

Jean Fan

Section Editor

PLOS Computational Biology

**Additional Editor Comments:**

The reviewers generally found the work timely and important but expressed a number of concerns that need to be addressed in a major revision. In addition to their concerns, the authors need to provide more methodological detail on how they quantified the severity of aganglionosis as this is critical for the validity of the rest of the work. There is a mention of acetylcholinesterase staining, but examples as a supplementary figure would be highly useful for readers.

We look forward to receiving your revised manuscript that addresses the reviewers' comments.

**Journal Requirements:**

1) Please ensure that the CRediT author contributions listed for every co-author are completed accurately and in full.

At this stage, the following Authors/Authors require contributions: Joseph T. Benthal, Justin A. Avila, Jeffrey R. Smith, and E Michelle Southard-Smith. Please ensure that the full contributions of each author are acknowledged in the "Add/Edit/Remove Authors" section of our submission form.

The list of CRediT author contributions may be found here: https://journals.plos.org/ploscompbiol/s/authorship#loc-author-contributions

2) Please upload all main figures as separate Figure files in .tif or .eps format. For more information about how to convert and format your figure files please see our guidelines:

https://journals.plos.org/ploscompbiol/s/figures

3) Some material included in your submission may be copyrighted. According to PLOSu2019s copyright policy, authors who use figures or other material (e.g., graphics, clipart, maps) from another author or copyright holder must demonstrate or obtain permission to publish this material under the Creative Commons Attribution 4.0 International (CC BY 4.0) License used by PLOS journals. Please closely review the details of PLOSu2019s copyright requirements here: PLOS Licenses and Copyright. If you need to request permissions from a copyright holder, you may use PLOS's Copyright Content Permission form.

Please respond directly to this email and provide any known details concerning your material's license terms and permissions required for reuse, even if you have not yet obtained copyright permissions or are unsure of your material's copyright compatibility. Once you have responded and addressed all other outstanding technical requirements, you may resubmit your manuscript within Editorial Manager.

Potential Copyright Issues:

- Figure 1. Please confirm whether you drew the images / clip-art within the figure panels by hand. If you did not draw the images, please provide (a) a link to the source of the images or icons and their license / terms of use; or (b) written permission from the copyright holder to publish the images or icons under our CC BY 4.0 license. Alternatively, you may replace the images with open source alternatives. See these open source resources you may use to replace images / clip-art:

- https://commons.wikimedia.org

- https://openclipart.org/.

4) Please amend your detailed Financial Disclosure statement. This is published with the article. It must therefore be completed in full sentences and contain the exact wording you wish to be published.

State what role the funders took in the study. If the funders had no role in your study, please state: "The funders had no role in study design, data collection and analysis, decision to publish, or preparation of the manuscript.".

If you did not receive any funding for this study, please simply state: u201cThe authors received no specific funding for this work.u201d

**Reviewers' comments:**

Reviewer's Responses to Questions

**Comments to the Authors:**

**Please note here if the review is uploaded as an attachment.**

Reviewer #1: This manuscript takes advantage of an unusually large ten generation Sox10Dom pedigree, encompassing 830 heterozygous mutants with quantitative aganglionosis measurements and genome wide SNP genotyping, and uses this resource to refine modifier loci that influence Hirschsprung disease severity (segment length). The authors integrate these genetic signals with multiple publicly available and ENS relevant single cell, bulk RNA seq, snATAC seq and TF motif datasets to prioritize biologically plausible modifiers, with particularly strong convergent support for Phox2b, Ednrb, Nrg1 and Dach1. The conceptual framework is clear, the analytical workflow is rigorous and transparent, and the main conclusions are well supported by the data.

This is an important contribution to the HSCR field because two long standing questions remain unresolved: the genetic basis of segment length variation and the genetic underpinnings of the pronounced sex difference in disease prevalence. Despite decades of work, neither has been fully dissected. This study directly tackles one of these gaps, namely the genetic architecture of segment length defects, by combining classical mouse genetics with multiomic integration to pinpoint candidate modifiers of aganglionosis severity. The approach, and the prioritization framework it introduces, represent a step toward resolving why the extent of aganglionosis varies so widely even among individuals or animals sharing the same primary pathogenic allele.

Conceptually, the work moves the field beyond broad, low resolution modifier loci and anecdotal candidate genes to a systematic and data driven strategy for modifier discovery. The integrative analysis that connects genetic loci to genes expressed in migrating ENCDCs, to chromatin accessibility in ENS progenitors and neuroblasts, and to human motility traits greatly strengthens the biological plausibility of the prioritized genes. Overall, the study provides a useful roadmap for future efforts aimed at delineating the polygenic contributions to HSCR severity.

Below are some suggested clarifications and analytical refinements to further strengthen the manuscript and maximize its impact.

1. The authors correctly note that the aganglionosis proportion is heavily zero inflated. They deal with this by 1) using GEMMA on the quantitative proportion with zeros included, and 2) converting to a pseudo binary affected vs unaffected phenotype.

• Consider including a brief comparison of models that treat the phenotype as

o a rank based transformed quantitative trait,

o an ordered categorical trait (for example none, short, intermediate, long), or

o a true zero inflated or hurdle model if available in a mixed model framework for mouse pedigrees.

• At minimum, provide residual diagnostics or some statement that the GEMMA assumptions are reasonably met for the proportion phenotype, and that the key loci (for example the Phox2b region on chr5 and the Dach1 region on chr14) are consistently detected across plausible transformations.

Even if the authors decide not to add new models, a short section in the Methods or Results that documents sensitivity analyses would strengthen confidence in the mapping.

2. The manuscript relies heavily on loci that are only nominally significant (p Wald less than 0.05) after multiple testing, and then brings in biological replication and multiomic evidence to prioritize these. This is conceptually reasonable for a highly pleiotropic trait with modest sample size, but could be framed more clearly:

• I suggest more explicitly separating

o genome wide significant loci (for example the Phox2b locus in males),

o FDR significant loci, and

o exploratory loci that are only nominally significant but supported by replication or omics integration.

• In Table 1 and the main text, it would help to mark which intervals are formally significant after correction in at least one analysis, and which are purely exploratory.

• The current modifier interval definition uses all contiguous SNPs with nominal p less than 0.05 plus or minus 0.5 Mb. Given the relatively sparse marker panel, this may generate quite wide intervals. The authors might provide summary statistics of interval sizes and, if possible, show that the main conclusions do not change with a slightly more stringent SNP inclusion threshold.

Clarifying this hierarchy will make the story more convincing and avoid overinterpretation of weak statistical signals.

3. Dach1 is a very appealing candidate, and the authors marshal several lines of evidence:

• Upregulation at the ENCDC wavefront,

• Expression in ENCDCs that increases over developmental time,

• Overlap between conserved SOX10 motifs and Dach1 intronic regions,

• snATAC peaks in ENS progenitors overlapping these motifs, and

• Proximity to human stool frequency loci and GI surgery phenotypes.

Given that there is no direct functional validation yet, I would suggest:

• Slightly softening the title and abstract language. For example, instead of “implicates Dach1 as a modifier” one could say “prioritizes Dach1 as a high confidence candidate modifier of ENS development.”

• Adding one or two relatively low effort analyses that leverage existing data, such as:

o Showing the specific alleles at the Dach1 containing SNPs in the pedigree and the effect size on aganglionosis proportion, perhaps stratified by sex.

o Examining coexpression of Dach1 with known ENS transcription factors (for example Sox10, Phox2b, Ret, Ednrb) in the Zhao scRNA seq dataset, for instance via a correlation heatmap across ENCDC subclusters.

4. The dual scoring system is a key contribution of the paper, but is currently somewhat buried in the text and supplementary tables.

• I recommend adding a compact main figure panel that shows, for the top approximately 15 genes, both the modifier interval score and the omics evidence score side by side, perhaps as a bar chart or heatmap.

• In the Methods, please spell out exactly how points were assigned, including whether replicated loci and sex specific analyses were weighted differently, and how genes that fell in multiple intervals were handled.

This will make it easier for readers to appreciate why Dach1, Phox2b, Ednrb, Nrg1 and others rise to the top.

5. The links to human stool frequency GWAS and PheWAS are intriguing, but currently described in a fairly qualitative way.

Within reason, the authors could strengthen this section by:

• Stating clearly which human loci are within the same LD blocks as the mouse orthologs and which are more distant, perhaps in a concise table.

• Clarifying whether effect directions for variants near candidate genes are consistent with increased or decreased motility.

• Describing the strategy for the text based search of ENS related phenotypes in Genebass, and acknowledging that this is exploratory.

This could remain a secondary part of the paper, but a bit more structure would help.

Minor comments and suggestions

1. Methodological clarity

o In the GEMMA methods, please state exactly which covariates were included (for example sex, batch, generation) and whether intestinal length was used as a covariate when modeling aganglionosis proportion.

For the snATAC seq analysis, consider giving a brief description of peak calling thresholds and how differential accessibility was defined (for example log fold change cutoffs, FDR thresholds).

2. Figure clarity

• In Manhattan plots, label the chromosomal positions of the key genes (Phox2b, Ednrb, Dach1) directly on the plots to guide readers.

• In the UMAPs that focus on ENCDCs, it would help to clearly label progenitor vs neuronal clusters and to add a schematic of gut colonization timing for readers less familiar with ENS development.

3. There are a few duplicated words or minor typos (for example “extended pedigree pedigree” in the abstract) that should be corrected.

4. The author should add the reference PMID: 30970187 in the introduction, where they discuss “human studies identified variants or genes that influence the penetrance of HSCR” (Line 117, page 5). This is to date the largest genome screen on HSCR.

Reviewer #2: Combinatorial multiomic analysis from a pedigree of Sox10Dom Hirschsprung mice implicates Dach1 as a modifier of Enteric Nervous System development.

General comments:

In this study Benthal et al set out to uncover the gene interactions that may underlie the variable severity of Hirschsprung’s (HSCR) disease. HSCR is neurocrisptopathy whereby neural crest (NC) derived enteric neurons fail to populate the enteric nervous system causing severe gastrointestinal defects in patients. A number of genes are well established to play a role in HSCR, including SOX10 which is the focal gene of this study. However, to date no study has uncovered the combinational action of genes in HSCR and how these combinations may influence the severity of disease.

For their study Benthal et al selected the Sox10dom mouse line, which is appropriate since this is a well-established Sox10 mutant line which displays HSCR (among other neural crest phenotypes). However, from the outset it is not clear how the authors quantified the severity of aganglionosis, there is mention of acetylcholinesterase staining, but this is not shown or elaborated on. Since this is crucial for the rest of the work presented, I have reviewed the manuscript assuming there has been some robust quantitative assessment of the severity of aganglionosis, if it so transpires that this is lacking, I’m afraid all subsequent analysis is unsubstantiable.

The study frequently refers to data/results from other studies, particularly Owens et al 2005. Arguably this helps to place the data within the broader context of the field, and it is good practice to utilise existing data, however it seems in places this study is quite dependent on previous work to substantiate the new analysis. In places this makes it quite challenging to unravel what is novel and meaningful from the current work.

The manuscript includes a large number of tables (8), if some of the data in these tables could be presented in a more tangible way that would greatly enhance the readability of the paper and better demonstrate the key findings.

The article is not cohesive; each results section presents some data but then this is largely unrelated to the next section. The authors don’t focus enough on any particular finding they just jump to the next analysis, e.g. they explore some gene expression patterns in single-cell RNA-seq data but then use bulk RNA-seq data to look at other genes, both gene sets are not explored in both data sets, which is disappointing and frustrating and indicates potential weaknesses in the current analysis if the findings are not consistent across different analysis pipelines.

Section specific comments:

Genome-wide SNP analysis identifies Sox10Dom aganglionosis modifiers

In this section the authors use Genome-wide Efficient Mixed Model Analysis (GEMMA) to predict causative SNPs in Sox10Dom animals. They use 830 pups, which seems excessive, was there a need for such high numbers of animals?

The authors mention comparing SNPs in animals that died younger, i.e. had more severe phenotype to those that survived to sexual maturity and therefore possibly had protective alleles. This would be a really interesting comparison and arguably essential to the question of which genes are involved in more severe cases of HSCR, but no such comparative data is included.

Did the authors look at any GWAS data from human HSCR patients, and did they observe any shared SNPs with their mouse model?

Did the authors ever cross female Sox10dom/+ with WT males and if so did they observe the same phenotype variability as when they crossed male Sox10dom/+ with WT females?

Figure 1A is not very informative. The text ‘Extent of aganglionosis was assessed by whole-mount acetylcholinesterase staining collected over multiple pedigree generations (Fig 1A; Supp. Table 1)’ suggests the acetylcholinesterase staining is shown here but it is not. Given this staining is the gold standard technique to diagnose HSCR disease and from the outset the authors plan to unravel the combinatorial causes of variable aganglionosis in HSCR it must be shown here.

Figure 1B, why was intestine length measured? This is not a phenotypic factor in HSCR disease, so this seems irrelevant. Furthermore, why were female and male intestine lengths compared? It is well established that this parameter is fairly consistent between sexes, a more relevant comparison would be intestine length in WT versus Sox10dom/+ animals. Although as my first point here, it would not be expected to see a difference in intestine length between WT and Sox10dom/+.

What data is presented in Figure 1D?

Table 1 is quite ‘dry’. Could the authors include some more tangible information, eg what is the nearest gene the SNPs region?

The GEMMA analysis is conducted on several variables, such as removing different chromosomes, male vs female etc. Whilst this broad analysis could be useful, no consensus seems to be made as to what predicted SNPs are most informative. On the Manhattan plots in Figure’s 2 and 3, what is the difference between the blue and black traces? The discovery of a SNP ~48kb upstream from Phoxb2, a known player in ENS development and function is surely of significant interest and yet this is not further explored. It is also notable that this SNP and one near Ednrb were attenuated in females compared to males, are their gender specific differences in HSCR genotype in humans? This is an important point and should be elaborated on.

Later in the paper the authors use different subsets of SNPs for further analysis, again muddying the waters as to what is really meaningful from their analysis.

Prioritization of candidate genes based on expression in the fetal mouse intestine

Following their GEMMA analysis, the authors use the UCSC table browser to predict genes associated with the SNP containing genomic intervals. It is not clear which intervals they annotated in this way, did they use a consensus set from all their GEMMA analyses or just those that met their aforementioned criteria ‘We prioritized consideration of associated loci that A) were significant after multiple testing correction, B) had a logarithm of the odds (LOD) score of ≥3, or C) replicated observations of an independent F1-intercross study (Owens et al. 2005)’ or did they include all ‘nominally significant’ intervals?

Is there any published 3C, or similar chromatin conformation capture data that could be used to determine if the SNPs lie within the topological associated domain of the ‘nearest’ gene?

Were changes in expression levels of these genes assessed in Sox10dom animals, or other HSCR models?

The authors go on to examine the expression of SNP associated genes in a previously published scRNA-seq data set. The the top female associated SNPs (lines 296 onwards) are discussed and their location in the scRNA-seq data is determined. Why is the focus on female SNPs here when in the GEMMA analyses it seemed many SNPs were attenuated in females compared to males. The female SNP associated genes, Mcm3 and Isl1 whilst indeed expressed in ENCDCs’, are also expressed in other populations at higher levels, but this is not discussed. Mcm3 was also detected in the ‘BothSexNo15X5’ GEMMA analysis, so this is not female specific. Isl1 is only expressed in ENCDC at 9.5, 10.5 and 11.5dpc, and only at very low levels. Is this meaningful? Did the authors further validate? Other genes seem more enriched in the ENCDCs, such as Uchl1, Limch1 and Arl8a but these are not discussed.

It would be interesting to see trajectory analysis across the single-cell RNA-seq time course, scVelo or similar, and to use this to infer which future populations might be affected by the SNPs.

There is no demonstration of co-expression of these modifier interval genes with Sox10, since this is the gene used in this particular model of HSCR it seems important to determine if other genes potentially contributing to the HSCR phenotype are expressed in the same cells.

Table 4 could be a venn diagram, assuming there is some overlap of modifier intervals falling into the same category?

The feature plots in Sup Fig 3 don’t show anything more than what is shown in figure 4. It would be better here if the colocalization of candidate genes and Sox10 was shown on the same plot.

CellChat data needs justification and much more explanation. I understand the authors are looking for combinations of factors effecting severity of aganglionosis but what prompted them to examine ligand/receptor interactions over transcription factor co-binding or epistatic relationships, what was the hypothesis here?

Exactly what data is presented in Sup Fig 4A? Many predicted pairs, including Nrg1/Erbb3 have no symbol along the plot so what is their meaning? The excel sheet in supp table 12 is far from self-explanatory. In the text the authors highlight the predicted interaction of Nrg1-Erbb3, however this relationship has been well documented, whilst this provides something of a validation of the approach the fact that even known players are not significant in their analysis is somewhat worrisome.

Differential expression of modifier interval genes in the migrating wavefront of enteric neural crest-derived migrating cells further prioritizes candidate genes.

In this section the authors turn to a bulk RNA-seq data set generated from migrating enteric neural crest cells. I understand this was done to further refine the expression profile of their candidate modifier interval genes from the GEMMA analysis, in neural crest cells. However, they are not showing the expression levels of the genes they previously assessed in the scRNA-seq data e.g. Mcm3, Isl1, why is this? Furthermore, did they look back in the scRNA-seq data for the expression profile of genes that were differentially expressed in the bulk neural crest RNA-seq data?

When the authors say… ‘We therefore prioritized genes within Sox10Dom aganglionosis modifier intervals that were differentially expressed in the ENCDC wavefront versus the lagging cells’ does this mean they restricted the differential gene expression analysis to just a handful of genes, if so what were these genes? Or were DEG detected globally but then they looked for modifier interval genes within the DEGs? Either way, on the volcano plot in figure 5B and as mentioned in the text, the gene Ulch1 was among those downregulated in wavefront NC cells. Since this gene was also enriched in ENCDC population in the scRNA data surely it is worthy of further investigation.

What were the cutoffs for significance of DEGs? What are the genes shown to have greater p-value and logFC scores on the volcano plot in fig 5B.

In the figure 5 legend; ‘D Expression of Sox10, Elavl4, and Phox2a to label progenitor and neuronal cells, respectively.’ It is not clear which gene(s) mark progenitor or neuronal cells. It reads like Sox10 and Elav4 mark progenitors, but Elav4 and Phox2a label the same cells so presumably these are neuronal? The plot from Sup Fig 5A might be more useful in the main figure 5.

‘However, wavefront upregulated genes are expressed in either a specific intermediate population (Col1a1, Tgfbi, Pdgfra)’ please highlight the imtermediate population on the feature plots.

Figure 5E, why are the downregulated genes not show across the time course like the upregulated genes are? Ptprg and dach1 do indeed increase in expression over the time course, but they are not refined to either progenitor or neuronal populations, which is of interest, but this is not elaborated on further. How do the up and downregulated genes look across the whole scRNA-seq data set, a plot of these similar to that in Fig 4C.

Table 5 could be removed, the GEMMA/GWAS categories in which each gene is found is shown by the legend in Fig 5E, for most genes.

Evolutionarily conserved SOX10 binding sites within Sox10Dom modifier intervals overlap or are near genes differentially expressed in the migrating wavefront

The information in table 6 would be more interpretable presented as position weight matrices.

Chromatin accessibility within enteric neuronal progenitors highlights putative regulatory regions within aganglionosis modifier intervals.

Here the authors generate single-nuclei ATAC-seq data to identify putative regulatory regions in which the modifier interval SNPs might be found. This is an appropriate experiment to characterise the genomic regions harbouring SNPs of interest. To this end they use the Phox2b mouse line to extract ENCDC’s, it would be helpful to include a picture of CFP+ tissue.

From how many gut’s did they get 13431 nuclei? Were these from male or female or a mix of both?

Following differential chromatin accessibility analysis it is convention to call these peaks ‘differential accessible regions’ (DARs), not DACs.

The authors use transcriptome data from E15.5 mice (total foetal gut tissue) to annotate their single nuclei ATAC data from E16.5 mice ENCDC’s. This is ok, but the authors should mention the caveat that they may be missing glial markers in this annotation since E15.5-16.5 covers the glial transition phase, glial markers won’t be present the in E15.5 transcriptome data. The authors could use promoter proximal peaks from their snATAC data to infer gene expression.

Figure 6A, the clusters should be annotated. The plots in 6A and 6B should be the same size, given their axis have the same scale.

The scatter plot in 6C is very difficult to read. I would suggest either refining the legend by giving a motif with multiple occurrences a shape, or by showing an inset scatter plot of the motif enrichment from just one modifier interval category.

Line 438, Annotations did not deviate across groups except for Neuroblast2…. For precision, this should say ‘Genomic annotations….’ Since annotation could also refer to the cell type assigned to a given cluster.

Two of the conserved SOX10 binding motifs overlapped with modifier interval DAC. These were intronic to Ranbp17 (close to Tlx3; chr11:33400347-33400362) and Dach1 (chr14:97891443-97891462; Table 6, Supp. Table 17). This should be shown on the scatter plot in 6C.

The complex filtering and comparisons of TF motifs in the snATAC data seems to just yield a list of TFs already known to play a role in ENS development. It would be more interesting/useful to discuss the predicted epistatic relationships between these factors.

Line 462, ‘Transcription factor (TF) binding is important for the transcription of genes…’ this should state ‘….TF binding to CRE’s is important for…..’

Line 466, ‘We focused on progenitors and neuroblasts because these are most similar to migrating ENCDCs’ By what proxy are progenitors and neuroblasts most similar to migrating ENCDC’s? Why is the focus now on migrating ENCDCs when the purpose of using the Phox2b line was to get a complete picture of ENCDCs when the gut was fully propagated with these cells?

Line 467, ‘This approach examined both open chromatin in progenitors and neuroblasts as well as regions that are closed in differentiating enteric neuronal cells’, how were differentiating enteric neuronal cells identified? These are not a separate cluster on the UMAP shown.

ATAC data predicts cis-regulatory elements (CRE). The authors have focused on TF motif enrichment, which is useful, but they could take it further by selecting the TF motif enriched DAC, within modifier intervals, and asking what are the cognate putative target genes of predicted CREs and how might they be relevant to HSCR.

Prioritization of top aganglionosis modifier candidate genes across data modalities

The flowchart in figure 7 presents a nice summary of the analysis pipeline and the text provides some clarity regarding the analysis done. However, it is still somewhat convoluted and difficult to entangle. For example, why were genes with a modifier interval score of 0 even included in the multiomics scoring system? A venn diagram might help demonstrate the findings from the dual scoring approach.

It would be interesting to know something about the shortlisted genes, e.g GO or IPA.

Candidate modifiers of aganglionosis related to human GI phenotypes

It is good that the authors have refined their gene lists by strain/background effects and then compared these to human data. However, how is the mouse genetic background relevant to human disease?

It is only in this final section that Dach1 is discussed, and without any tangible link to HSCR or other ENS conditions it may not be appropriate to have these gene in the title of the paper, since the paper isn’t really focussed on the Dach1finding, in fact this rather discredits all the other work the authors have performed.

The discussion section is somewhat repeative of the results and in other places seems to include some new data, or at least different number of genes in different categories, e.g. lines 606-608. It would greatly improve the readability and justification of approaches if the results and discussion section were combined.

Concluding comments:

The authors have conducted an extensive analysis of transcriptomes and chromatin accessibility data from mice ENS. Whilst vast, and as such applaudable, much of the analysis is not validated. The authors do recognise this, and modestly other limitations of the study, and I appreciate this paper has come to PLOS Computational Biology and therefore functional validation is not required/appropriate. However, in this era of interdisciplinary research it is not unreasonable to expect some in vivo, functional validation of key findings from computational studies e.g. expression of Dach1 in WT versus HSCR model mice gut, especially since this lab does have wet lab expertise as well as computational.

**********

**Have the authors made all data and (if applicable) computational code underlying the findings in their manuscript fully available?**

The PLOS Data policy requires authors to make all data and code underlying the findings described in their manuscript fully available without restriction, with rare exception (please refer to the Data Availability Statement in the manuscript PDF file). The data and code should be provided as part of the manuscript or its supporting information, or deposited to a public repository. For example, in addition to summary statistics, the data points behind means, medians and variance measures should be available. If there are restrictions on publicly sharing data or code —e.g. participant privacy or use of data from a third party—those must be specified.

Reviewer #1: Yes

Reviewer #2: Yes

**********

PLOS authors have the option to publish the peer review history of their article (what does this mean?). If published, this will include your full peer review and any attached files.

If you choose “no”, your identity will remain anonymous but your review may still be made public.

**Do you want your identity to be public for this peer review?**  For information about this choice, including consent withdrawal, please see our Privacy Policy.

Reviewer #1: **Yes:** Sumantra Chatterjee

Reviewer #2: No

[NOTE: If reviewer comments were submitted as an attachment file, they will be attached to this email and accessible via the submission site. Please log into your account, locate the manuscript record, and check for the action link "View Attachments". If this link does not appear, there are no attachment files.]

**Figure resubmission:**

While revising your submission, we strongly recommend that you use PLOS’s NAAS tool (https://ngplosjournals.pagemajik.ai/artanalysis) to test your figure files. NAAS can convert your figure files to the TIFF file type and meet basic requirements (such as print size, resolution), or provide you with a report on issues that do not meet our requirements and that NAAS cannot fix.

After uploading your figures to PLOS’s NAAS tool - https://ngplosjournals.pagemajik.ai/artanalysis, NAAS will process the files provided and display the results in the "Uploaded Files" section of the page as the processing is complete. If the uploaded figures meet our requirements (or NAAS is able to fix the files to meet our requirements), the figure will be marked as "fixed" above. If NAAS is unable to fix the files, a red "failed" label will appear above. When NAAS has confirmed that the figure files meet our requirements, please download the file via the download option, and include these NAAS processed figure files when submitting your revised manuscript.

**Reproducibility:**

To enhance the reproducibility of your results, we recommend that authors of applicable studies deposit laboratory protocols in protocols.io, where a protocol can be assigned its own identifier (DOI) such that it can be cited independently in the future. Additionally, PLOS ONE offers an option to publish peer-reviewed clinical study protocols. Read more information on sharing protocols at https://plos.org/protocols?utm_medium=editorial-email&utm_source=authorletters&utm_campaign=protocols

# Author comment: Author response to Decision Letter 1

**DOI:** 10.1371/journal.pcbi.1014424.r002

8 Apr 2026

Attachment Submitted filename: 2026-04-06_Response to Review PCOMPBIOL-D-25-02281.pdf

# Peer review: Decision Letter 1

**DOI:** 10.1371/journal.pcbi.1014424.r003

**Authors:** Mark Ziemann, Jean Fan, Mark Ziemann, Jean Fan

29 Apr 2026

PCOMPBIOL-D-25-02281R1

Combinatorial multiomic analysis from a pedigree of Sox10Dom Hirschsprung mice identifies multiple high confidence candidate modifiers of Enteric Nervous System development

PLOS Computational Biology

Dear Dr. Southard-Smith,

Thank you for submitting your manuscript to PLOS Computational Biology. After careful consideration, we feel that it has merit but does not fully meet PLOS Computational Biology's publication criteria as it currently stands. Therefore, we invite you to submit a revised version of the manuscript that addresses the points raised during the review process.

Please submit your revised manuscript by Jun 29 2026 11:59PM. If you will need more time than this to complete your revisions, please reply to this message or contact the journal office at ploscompbiol@plos.org. When you're ready to submit your revision, log on to https://www.editorialmanager.com/pcompbiol/ and select the 'Submissions Needing Revision' folder to locate your manuscript file.

Please include the following items when submitting your revised manuscript:

* A letter that responds to each point raised by the editor and reviewer(s). You should upload this letter as a separate file labeled 'Response to Reviewers'. This file does not need to include responses to formatting updates and technical items listed in the 'Journal Requirements' section below.

* A marked-up copy of your manuscript that highlights changes made to the original version. You should upload this as a separate file labeled 'Revised Manuscript with Track Changes'.

* An unmarked version of your revised paper without tracked changes. You should upload this as a separate file labeled 'Manuscript'.

If you would like to make changes to your financial disclosure, competing interests statement, or data availability statement, please make these updates within the submission form at the time of resubmission. Guidelines for resubmitting your figure files are available below the reviewer comments at the end of this letter.

We look forward to receiving your revised manuscript.

Kind regards,

Mark Ziemann

Academic Editor

PLOS Computational Biology

Jean Fan

Section Editor

PLOS Computational Biology

**Additional Editor Comments:**

While Reviewer 2 is satisfied with the changes, Reviewer 1 has two remaining concerns. Please check the attached word document for specific details.

**Journal Requirements:**

We have noticed that you have uploaded Supporting Information files, but you have not included a list of legends. Please add a full list of legends for your Supporting Information files after the references list.

**Reviewers' comments:**

Reviewer's Responses to Questions

**Comments to the Authors:**

**Please note here if the review is uploaded as an attachment.**

Reviewer #1: Review is uploded as attachement

Reviewer #2: The authors have addressed all points raised in the review to the best of their abilities and within the scope of this study. This has enhanced the readability of the manuscript and clarity of the approaches taken and results therefrom.

**********

**Have the authors made all data and (if applicable) computational code underlying the findings in their manuscript fully available?**

The PLOS Data policy requires authors to make all data and code underlying the findings described in their manuscript fully available without restriction, with rare exception (please refer to the Data Availability Statement in the manuscript PDF file). The data and code should be provided as part of the manuscript or its supporting information, or deposited to a public repository. For example, in addition to summary statistics, the data points behind means, medians and variance measures should be available. If there are restrictions on publicly sharing data or code —e.g. participant privacy or use of data from a third party—those must be specified.

Reviewer #1: Yes

Reviewer #2: Yes

**********

PLOS authors have the option to publish the peer review history of their article (what does this mean?). If published, this will include your full peer review and any attached files.

If you choose “no”, your identity will remain anonymous but your review may still be made public.

**Do you want your identity to be public for this peer review?**  For information about this choice, including consent withdrawal, please see our Privacy Policy.

Reviewer #1: No

Reviewer #2: No

[NOTE: If reviewer comments were submitted as an attachment file, they will be attached to this email and accessible via the submission site. Please log into your account, locate the manuscript record, and check for the action link "View Attachments". If this link does not appear, there are no attachment files.]

**Figure resubmission:**

-->While revising your submission, we strongly recommend that you use PLOS’s NAAS tool (https://ngplosjournals.pagemajik.ai/artanalysis) to test your figure files. NAAS can convert your figure files to the TIFF file type and meet basic requirements (such as print size, resolution), or provide you with a report on issues that do not meet our requirements and that NAAS cannot fix.

After uploading your figures to PLOS’s NAAS tool - https://ngplosjournals.pagemajik.ai/artanalysis, NAAS will process the files provided and display the results in the "Uploaded Files" section of the page as the processing is complete. If the uploaded figures meet our requirements (or NAAS is able to fix the files to meet our requirements), the figure will be marked as "fixed" above. If NAAS is unable to fix the files, a red "failed" label will appear above. When NAAS has confirmed that the figure files meet our requirements, please download the file via the download option, and include these NAAS processed figure files when submitting your revised manuscript.

**Reproducibility:**

To enhance the reproducibility of your results, we recommend that authors of applicable studies deposit laboratory protocols in protocols.io, where a protocol can be assigned its own identifier (DOI) such that it can be cited independently in the future. Additionally, PLOS ONE offers an option to publish peer-reviewed clinical study protocols. Read more information on sharing protocols at https://plos.org/protocols?utm_medium=editorial-email&utm_source=authorletters&utm_campaign=protocols

Attachment Submitted filename: Benthal_etal_review round 2.docx

# Author comment: Author response to Decision Letter 2

**DOI:** 10.1371/journal.pcbi.1014424.r004

1 Jun 2026

Attachment Submitted filename: Benthal_etal_review round 2_Response_Upload.pdf

# Peer review: Decision Letter 2

**DOI:** 10.1371/journal.pcbi.1014424.r005

**Authors:** Mark Ziemann, Jean Fan, Mark Ziemann, Jean Fan, Mark Ziemann, Jean Fan

9 Jun 2026

Dear Dr. Southard-Smith,

We are pleased to inform you that your manuscript 'Combinatorial multiomic analysis from a pedigree of Sox10Dom Hirschsprung mice identifies multiple high confidence candidate modifiers of Enteric Nervous System development' has been provisionally accepted for publication in PLOS Computational Biology.

Before your manuscript can be formally accepted you will need to complete some formatting changes, which you will receive in a follow up email. A member of our team will be in touch with a set of requests.

Please note that your manuscript will not be scheduled for publication until you have made the required changes, so a swift response is appreciated.

IMPORTANT: The editorial review process is now complete. PLOS will only permit corrections to spelling, formatting or significant scientific errors from this point onwards. Requests for major changes, or any which affect the scientific understanding of your work, will cause delays to the publication date of your manuscript.

Should you, your institution's press office or the journal office choose to press release your paper, you will automatically be opted out of early publication. We ask that you notify us now if you or your institution is planning to press release the article. All press must be co-ordinated with PLOS.

Thank you again for supporting Open Access publishing; we are looking forward to publishing your work in PLOS Computational Biology.

Best regards,

Mark Ziemann

Academic Editor

PLOS Computational Biology

Jean Fan

Section Editor

PLOS Computational Biology

***********************************************************

Reviewer's Responses to Questions

**Comments to the Authors:**

**Please note here if the review is uploaded as an attachment.**

Reviewer #1: I thank the authors for completing all suggested changes. I have no further comments. this paper is a very useful contribution to the field

**********

**Have the authors made all data and (if applicable) computational code underlying the findings in their manuscript fully available?**

The PLOS Data policy requires authors to make all data and code underlying the findings described in their manuscript fully available without restriction, with rare exception (please refer to the Data Availability Statement in the manuscript PDF file). The data and code should be provided as part of the manuscript or its supporting information, or deposited to a public repository. For example, in addition to summary statistics, the data points behind means, medians and variance measures should be available. If there are restrictions on publicly sharing data or code —e.g. participant privacy or use of data from a third party—those must be specified.

Reviewer #1: Yes

**********

PLOS authors have the option to publish the peer review history of their article (what does this mean?). If published, this will include your full peer review and any attached files.

If you choose “no”, your identity will remain anonymous but your review may still be made public.

**Do you want your identity to be public for this peer review?**  For information about this choice, including consent withdrawal, please see our Privacy Policy.

Reviewer #1: **Yes:** Sumantra Chatterjee

# Editor report: Acceptance letter

**DOI:** 10.1371/journal.pcbi.1014424.r006

**Authors:** Mark Ziemann, Jean Fan, Mark Ziemann, Jean Fan, Mark Ziemann, Jean Fan

PCOMPBIOL-D-25-02281R2

Combinatorial multiomic analysis from a pedigree of Sox10Dom Hirschsprung mice identifies multiple high confidence candidate modifiers of Enteric Nervous System development

Dear Dr Southard-Smith,

I am pleased to inform you that your manuscript has been formally accepted for publication in PLOS Computational Biology. Your manuscript is now with our production department and you will be notified of the publication date in due course.

The corresponding author will soon be receiving a typeset proof for review, to ensure errors have not been introduced during production. Please review the PDF proof of your manuscript carefully, as this is the last chance to correct any errors. Please note that major changes, or those which affect the scientific understanding of the work, will likely cause delays to the publication date of your manuscript.

Soon after your final files are uploaded, unless you have opted out, the early version of your manuscript will be published online. The date of the early version will be your article's publication date. The final article will be published to the same URL, and all versions of the paper will be accessible to readers.

For Research, Software, and Methods articles, you will receive an invoice from PLOS for your publication fee after your manuscript has reached the completed accept phase. If you receive an email requesting payment before acceptance or for any other service, this may be a phishing scheme. Learn how to identify phishing emails and protect your accounts at https://explore.plos.org/phishing.

Thank you again for supporting PLOS Computational Biology and open-access publishing. We are looking forward to publishing your work!

With kind regards,

Anitha Samidurai

PLOS Computational Biology | Carlyle House, Carlyle Road, Cambridge CB4 3DN | United Kingdom ploscompbiol@plos.org | Phone +44 (0) 1223-442824 | ploscompbiol.org | @PLOSCompBiol
